# Human T_SCM_ cell dynamics in vivo are compatible with long-lived immunological memory and stemness

**DOI:** 10.1371/journal.pbio.2005523

**Published:** 2018-06-22

**Authors:** Pedro Costa del Amo, Julio Lahoz-Beneytez, Lies Boelen, Raya Ahmed, Kelly L. Miners, Yan Zhang, Laureline Roger, Rhiannon E. Jones, Silvia A. Fuertes Marraco, Daniel E. Speiser, Duncan M. Baird, David A. Price, Kristin Ladell, Derek Macallan, Becca Asquith

**Affiliations:** 1 Department of Medicine, Imperial College London, London, United Kingdom; 2 Institute for Infection and Immunity, St George’s, University of London, London, United Kingdom; 3 Division of Infection and Immunity, Cardiff University School of Medicine, Cardiff, United Kingdom; 4 Division of Cancer and Genetics, Cardiff University School of Medicine, Cardiff, United Kingdom; 5 Department of Oncology, Lausanne University Hospital, Lausanne, Switzerland; 6 St George’s University Hospitals NHS Foundation Trust, London, United Kingdom; National Cancer Institute, United States of America

## Abstract

Adaptive immunity relies on the generation and maintenance of memory T cells to provide protection against repeated antigen exposure. It has been hypothesised that a self-renewing population of T cells, named stem cell–like memory T (T_SCM_) cells, are responsible for maintaining memory. However, it is not clear if the dynamics of T_SCM_ cells in vivo are compatible with this hypothesis. To address this issue, we investigated the dynamics of T_SCM_ cells under physiological conditions in humans in vivo using a multidisciplinary approach that combines mathematical modelling, stable isotope labelling, telomere length analysis, and cross-sectional data from vaccine recipients. We show that, unexpectedly, the average longevity of a T_SCM_ clone is very short (half-life < 1 year, degree of self-renewal = 430 days): far too short to constitute a stem cell population. However, we also find that the T_SCM_ population is comprised of at least 2 kinetically distinct subpopulations that turn over at different rates. Whilst one subpopulation is rapidly replaced (half-life = 5 months) and explains the rapid average turnover of the bulk T_SCM_ population, the half-life of the other T_SCM_ subpopulation is approximately 9 years, consistent with the longevity of the recall response. We also show that this latter population exhibited a high degree of self-renewal, with a cell residing without dying or differentiating for 15% of our lifetime. Finally, although small, the population was not subject to excessive stochasticity. We conclude that the majority of T_SCM_ cells are not stem cell–like but that there is a subpopulation of T_SCM_ cells whose dynamics are compatible with their putative role in the maintenance of T cell memory.

## Author summary

The human immune system remembers previously encountered pathogens so that, on meeting the same pathogen a second time, the response is quicker and more effective. This immune memory is the basis of all vaccinations. Immune memory persists for decades, but how memory is maintained is unclear. It has been hypothesised that there is a dedicated population of cells called stem cell–like memory T (T_SCM_) cells that have stem cell–like behaviour and are responsible for the persistence of T cell memory. Here, we show that a subset of T_SCM_ cells, in healthy humans in vivo, have the dynamic properties of self-renewal and clonal longevity necessary to maintain long-lived immune memory.

## Introduction

The maintenance of long-lived T cell memory is one of the hallmarks of adaptive immunity [1, 2]. Multiple studies have shown that the recall response to a previously encountered antigen has a half-life of the order of decades [3, 4]. It has been hypothesised that this T cell memory is dynamically maintained by differentiation of a precursor stem cell–like memory population [5]. Alternative, nonexclusive explanations include replacement by proliferation of differentiated memory T cells or the existence of a putative subpopulation of long-lived memory T cells that has not yet been identified, either because such cells are very rare or because they reside primarily outside of the peripheral blood [6–9].

Central memory T (T_CM_) cells (CD45RA^―^CCR7^+^ in humans) were previously thought to constitute the stem cell–like memory precursor population. Evidence supporting the ‘stemness’ of T_CM_ cells includes their capacity to differentiate into effector memory T (T_EM_) cells and T effector (T_EFF_) cells [10, 11]. This hypothesis was further strengthened by cell fate–tracking experiments in mice (using genetic barcoding and single-cell transfer), showing that T_CM_ cells had the capacity to self-renew and that a single T_CM_ cell could reconstitute immune protection against an otherwise lethal pathogen [12, 13].

However, the concept of T_CM_ as the stem cell population has been challenged by the identification of ‘stem cell–like’ memory T (T_SCM_) cells—which have enhanced stem cell–like properties compared to T_CM_ cells—in mice [14], nonhuman primates [15], and humans [16]. In humans, like naïve cells, T_SCM_ cells are CD45RA^+^CD45RO^―^, and they express high levels of CD27, CD28, interleukin 7 receptor alpha (IL-7Rα), CD62L, and C-C chemokine receptor 7 (CCR7). Unlike naïve cells, T_SCM_ cells are clonally expanded and express the memory markers CD95 and CD122 [1, 16]. T_SCM_ cells exhibit enhanced proliferative capacity compared to T_CM_ cells, the potential to differentiate into all other classically defined T cell memory subsets (including T_CM_), and the ability to retain their phenotype following proliferation both in vitro and in mice in vivo [1, 14–16]. In light of these observations, it has been suggested that T_SCM_ cells are the main stem cell memory population and play a key role in maintaining long-term memory in vivo [15–18].

There are 3 basic prerequisites for T cell memory stemness: multipotency, self-renewal, and clonal longevity. In this study, we focus on the related dynamic properties of self-renewal and clonal longevity. Self-renewal of human T_SCM_ cells has been demonstrated in vitro [19], but it remains a concern that the local microenvironment, which may crucially affect the degree of self-renewal, will be different in vivo and in vitro. However, proving self-renewal of human T_SCM_ cells in vivo has so far not been possible because of ethical and technical limitations. The second property we investigate is clonal longevity. Long-lived T cell memory requires that memory T cell clonotypes expressing the same T cell receptor (TCR) persist for several decades in vivo. For example, influenza immunity has been shown to last for several decades [20], and small pox vaccine–induced T cell memory has a half-life of 8–15 years [3, 4]. For T_SCM_ cells to constitute a potential precursor population for T cell memory in vivo, the survival of T_SCM_ clones needs to be consistent with those estimates. A number of studies suggest that T_SCM_ clones can survive for several years. Biasco and colleagues [17] observed that genetically engineered T_SCM_ cells could persist for many years in patients suffering from severe combined immunodeficiency disease. Fuertes Marraco and colleagues [21] identified a yellow fever virus (YFV)-specific T_SCM_ population up to 25 years after vaccination. Finally, in leukaemia patients who had undergone haematopoietic stem cell (HSC) transplantation, Oliveira and colleagues [22] reported that gene-modified T_SCM_ cells could be detected in the circulation up to 14 years after treatment. These studies support the concept of T_SCM_ longevity, albeit in scenarios of lymphocyte depletion or profound CD8^+^ T cell expansion. However, it has been shown that the dynamics of posttransplant haematopoiesis in mice differs significantly from normal, unperturbed haematopoiesis [23–25], and so it cannot be assumed that these transplantation studies in humans necessarily recapitulate the healthy human system. In short, T_SCM_ longevity has not been quantified in normal, unperturbed homeostasis in humans, and the related question of the ability of T_SCM_ to self-renew has not been addressed in any human in vivo system.

In order to investigate human T_SCM_ cells in homeostasis, we previously performed stable isotope labelling of healthy volunteers and analysed label uptake in CD4^+^ and CD8^+^, naïve T and T_SCM_ cells. We found that the T_SCM_ population was rapidly turning over (median 0.02 per day, interquantile range 0.016–0.037 per day, half-life < 1 year) and concluded that the T_SCM_ population is dynamically maintained [26]. However, in this previous study, only labelling data were modelled, and so it was not possible to address the central question of the ‘stemness’ of the T_SCM_ pool. First, to constitute a stem cell population, it is not enough to have a stably maintained population of cells; stemness requires long-term clonal persistence [18]. That is, whilst the size of the T_SCM_ population as a whole may be stably maintained, the lifespan of any given antigen-specific precursor population could be short; such limited lifespans would be difficult to reconcile with the hypothesis that T_SCM_ cells are the repository of T cell memory. Second, the high turnover rates obtained in this labelling study [26] do not necessarily indicate that the majority of the T_SCM_ population is replaced by the self-renewal of the T_SCM_ pool; frequent naïve cell differentiation could also be responsible. Indeed, given the very large size of the naïve pool compared to the T_SCM_ pool [19], a relatively low proportion of proliferating naïve cells would be sufficient to replace lost T_SCM_ cells. In this scenario, T_SCM_ cells would simply represent transit cells on the differentiation pathway from naïve to effector rather than self-renewing stem cells.

Here, we investigate whether the dynamics of T_SCM_ cells in healthy humans are consistent with their putative role as memory stem cells. Specifically, we investigate both the capacity of T_SCM_ cells to self-renew and the longevity of T_SCM_ clones. It is challenging to address these questions in humans, and they cannot be answered using stable isotope labelling alone, since different scenarios (e.g., ‘all new T_SCM_ cells come from naïve cell differentiation’ versus ‘all new T_SCM_ cells come from T_SCM_ proliferation’) can give rise to very similar levels of label in the T_SCM_ population. To enable us to deconvolute these possibilities, we performed telomere length analysis and utilised cross-sectional T_SCM_ cell data from YFV vaccine recipients. Deterministic and stochastic mechanistic mathematical modelling were then used to analyse all 3 datasets. This novel approach allows us to investigate human T_SCM_ cell dynamics in vivo and to address questions previously only investigated in animal models.

## Results

**Fig 1:**
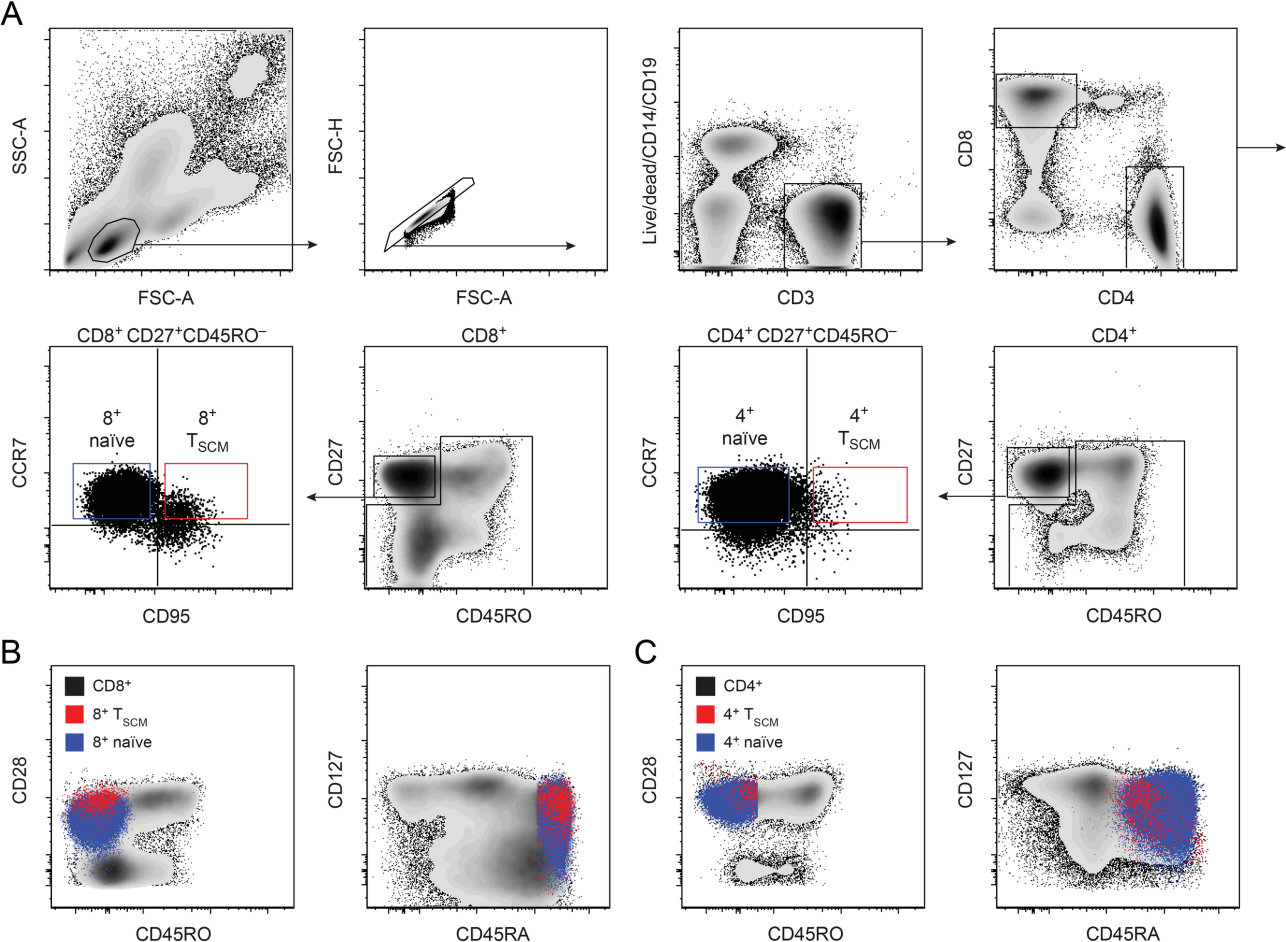
Cell surface phenotype of naïve and T_SCM_ populations. (A) Gating strategy used to sort CD4^+^ and CD8^+^ naïve and T_SCM_ cells for isotope label and telomere length analysis: the top panels show the consecutive gating to detect CD8^+^ or CD4^+^ T cells; the bottom panels show the further gating to detect naïve or T_SCM_ cells within CD8^+^ or CD4^+^ populations. (B) Expression of CD45RO, CD28, CD127, and CD45RA on CD8^+^ CCR7^+^ CD95^−^ naïve cells (blue cloud) and CD8^+^ CCR7^+^ CD95^+^ T_SCM_ cells (red cloud) compared with bulk CD8^+^ T cells (black cloud). (C) as for B but depicting CD4^+^ naïve and CD4^+^ T_SCM_ compared to bulk CD4^+^ T cells. FSC-A, forward scatter area; FSC-H, forward scatter height; SSC-A, side scatter area; T_SCM_, stem cell–like memory T.

**Fig 2:**
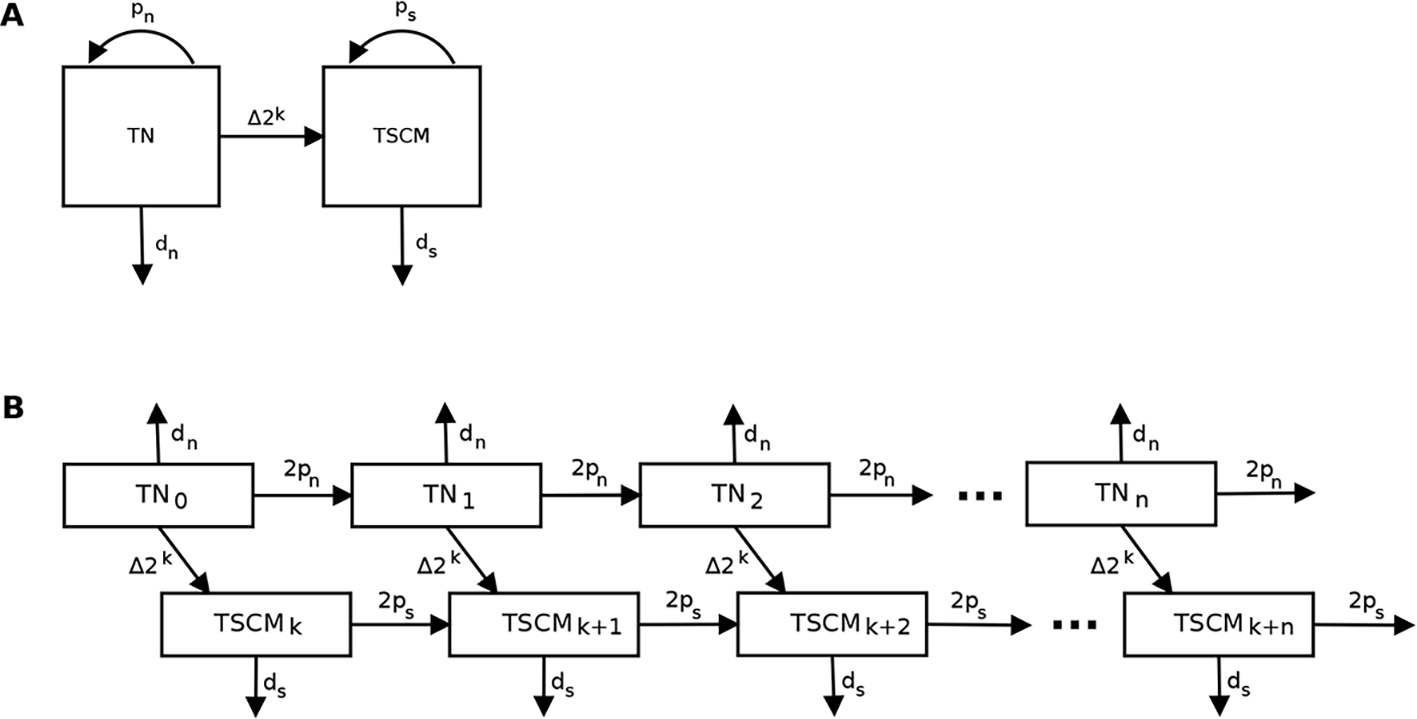
Model to describe the T_N_ and T_SCM_ populations. (A) Schematic representation of the model for T_N_ and T_SCM_ populations. (B) Schematic representation of the model for telomere length data for T_N_ and T_SCM_ populations when *C* = *k* (inactive telomerase). T_Ni_ (or T_SCMi_) represent the number of T_N_ (or T_SCM_) cells which have divided *i* times; *p*_*n*_, *p*_*s*_, *d*_*n*_, and *d*_*s*_ are the proliferation and disappearance rates of T_N_ and T_SCM_ populations; *Δ* is the fraction of T_N_ cells recruited per day, and *k* is the number of divisions that occur during clonal expansion. T_N_, naïve T; T_SCM_, stem cell–like memory T.

To investigate the dynamics of T_SCM_ cells in humans in vivo, we analysed experimental data that we obtained in a 7-week stable isotope–labelling study of 5 healthy individuals [26], in which label incorporation into CD4^+^ and CD8^+^ naïve (CD45RA^bright^CD27^bright^CCR7^+^CD95^−^) and T_SCM_ (CD45RA^bright^CD27^bright^CCR7^+^CD95^+^) cells was measured at multiple time points (Fig 1). In addition, we performed single-telomere length analysis of each cell population in the same individuals. We then constructed an ordinary differential equation (ODE)-based mathematical model to describe both stable isotope (heavy water) labelling and telomere length, in which we assumed a linear differentiation pathway from naïve to T_SCM_ cells (Fig 2, Methods [27]).

### Heterogeneity in the T_SCM_ population

**Fig 3:**
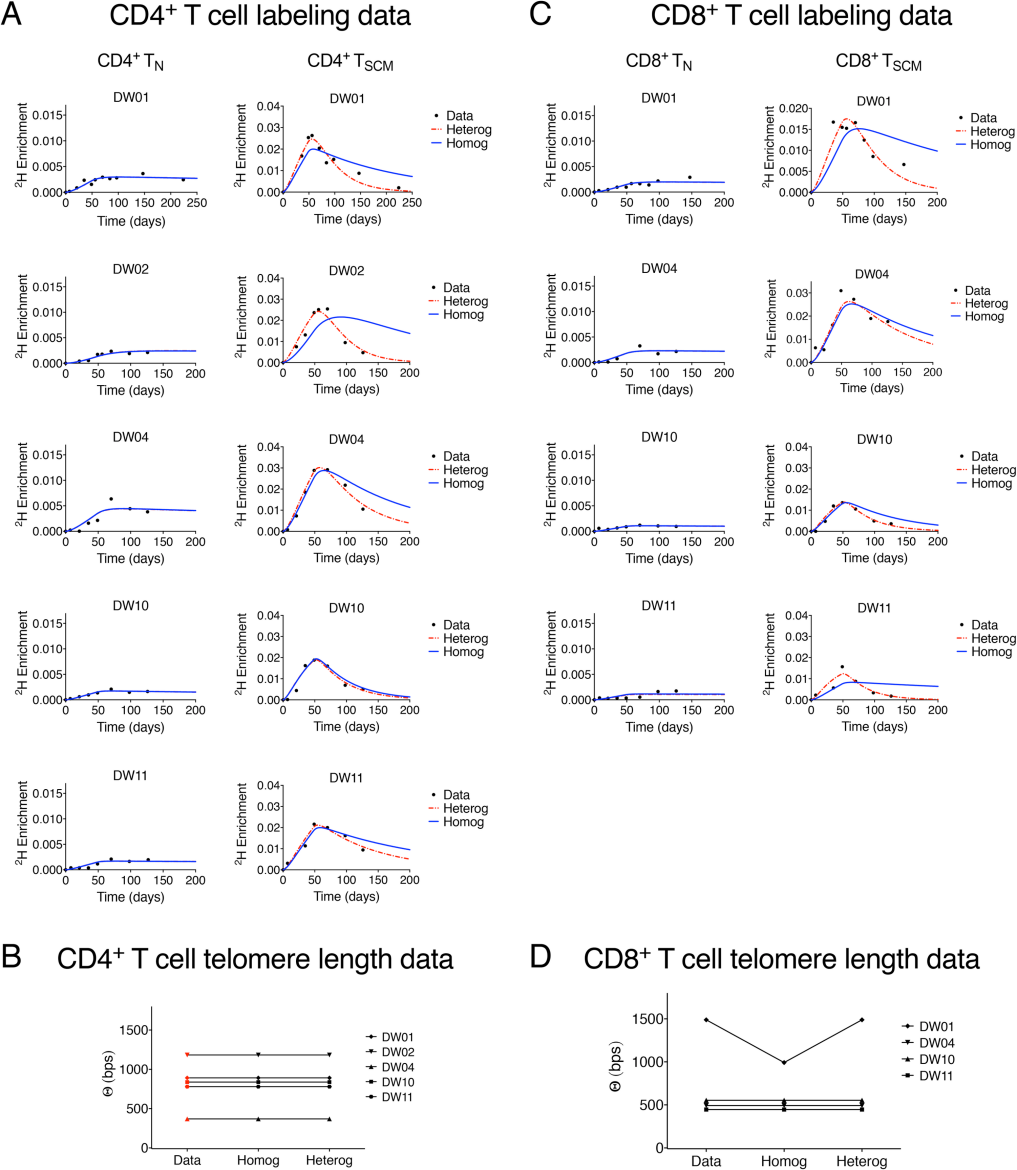
Label incorporation and telomere length in CD4^+^ and CD8^+^ naïve T and T_SCM_ cells. (A) Experimental labelling data (black dots) and best fit of model to the data assuming kinetic homogeneity (blue solid line) and kinetic heterogeneity (red dashed line) of the CD4^+^ T_N_ pool (left column) and the CD4^+^ T_SCM_ pool (right column). (B) Experimental measurement (red symbol) and best fit of the homogeneous (‘homog’) and heterogeneous (‘heterog’) models (black symbols) to the average telomere length differences (*Θ*) between CD4^+^ T_N_ and CD4^+^ T_SCM_ cells. Labelling data and telomere length data were fitted simultaneously. We found strong evidence to reject the null hypothesis of homogeneity in the CD4^+^ T_SCM_ population (median *P* = 5.8 × 10^−7^, pooled *P* = 3.5 × 10^−23^) but not in the CD4^+^ T_N_ population (median *P* = 0.6, pooled *P* = 0.9). (C) and (D) as for A and B but for CD8^+^ cells rather than CD4^+^ cells. Again, for CD8^+^ cells as for CD4^+^, we found strong evidence to reject the null hypothesis of homogeneity in the T_SCM_ population (median *P* = 4.1 × 10^−6^, pooled *P* = 6.1 × 10^−15^) but not in the T_N_ population (median *P* = 0.5, pooled *P* = 0.7). Experimental data depicted in this figure can be found in S1 Data. T_N_, naïve T; T_SCM_, stem cell–like memory T.

Estimates of the rate of T_SCM_ renewal and T_SCM_ clonal lifespan will depend upon the kinetic structure of the T_SCM_ pool. We therefore first asked whether there was evidence for kinetic heterogeneity (i.e., existence of subpopulations with differing kinetics) within the T_SCM_ pool by comparing the quality of fit of a homogenous and heterogeneous version of the mathematical model. In the homogeneous version of the model, we constrain the input rate (proliferation rate + rate of new entrants due to differentiation from naïve cells) of the whole T_SCM_ population to be equal to the disappearance rate of labelled T_SCM_ cells; this condition will be met for a kinetically homogenous population of constant size. In the heterogeneous version of the model, this constraint was relaxed to allow for the possibility of kinetic heterogeneity in the T_SCM_ pool (Methods, [28]). We used this implicit description of kinetic heterogeneity rather than an explicit description of the subpopulations because it requires fewer parameters (2 compared with 3 for the explicit model [28–30]) and furthermore does not suffer from the parameter identifiability issue inherent in the explicit kinetic heterogeneity model, which arises due to the very strong correlation between the proliferation rate and size of a subpopulation [31]. A total of 9 datasets were included in this analysis, representing CD4^+^ and CD8^+^ T cells (naïve and T_SCM_) from 5 individuals (1 CD8^+^ T_SCM_ cell dataset from 1 subject was not available). We found that, in 7 out of the 9 cases, constraining the T_SCM_ population to be homogeneous resulted in a substantially worse description of the data (Fig 3); and there was strong evidence to reject the assumption of homogeneity *P* = 5.8 × 10^−7^, *P* = 4.1 × 10^−6^ (median of *p*-values calculated using Fisher’s F-test for nested models between the homogeneous and heterogeneous models for CD4^+^ and CD8^+^ T_SCM_, respectively), indicating considerable support for the heterogeneous description of the T_SCM_ pool in both CD4^+^ and CD8^+^ T cell populations (S1 Table). In contrast, there was no evidence to reject the null hypothesis of homogeneity in the naïve cell pool (*P* = 0.6, *P* = 0.5, for CD4^+^ and CD8^+^ T_N_, respectively; median of *p*-values calculated using Fisher’s F-test for nested models between the homogeneous and heterogeneous models; S1 Table).

### Magnitude of clonal expansion

**Fig 4:**
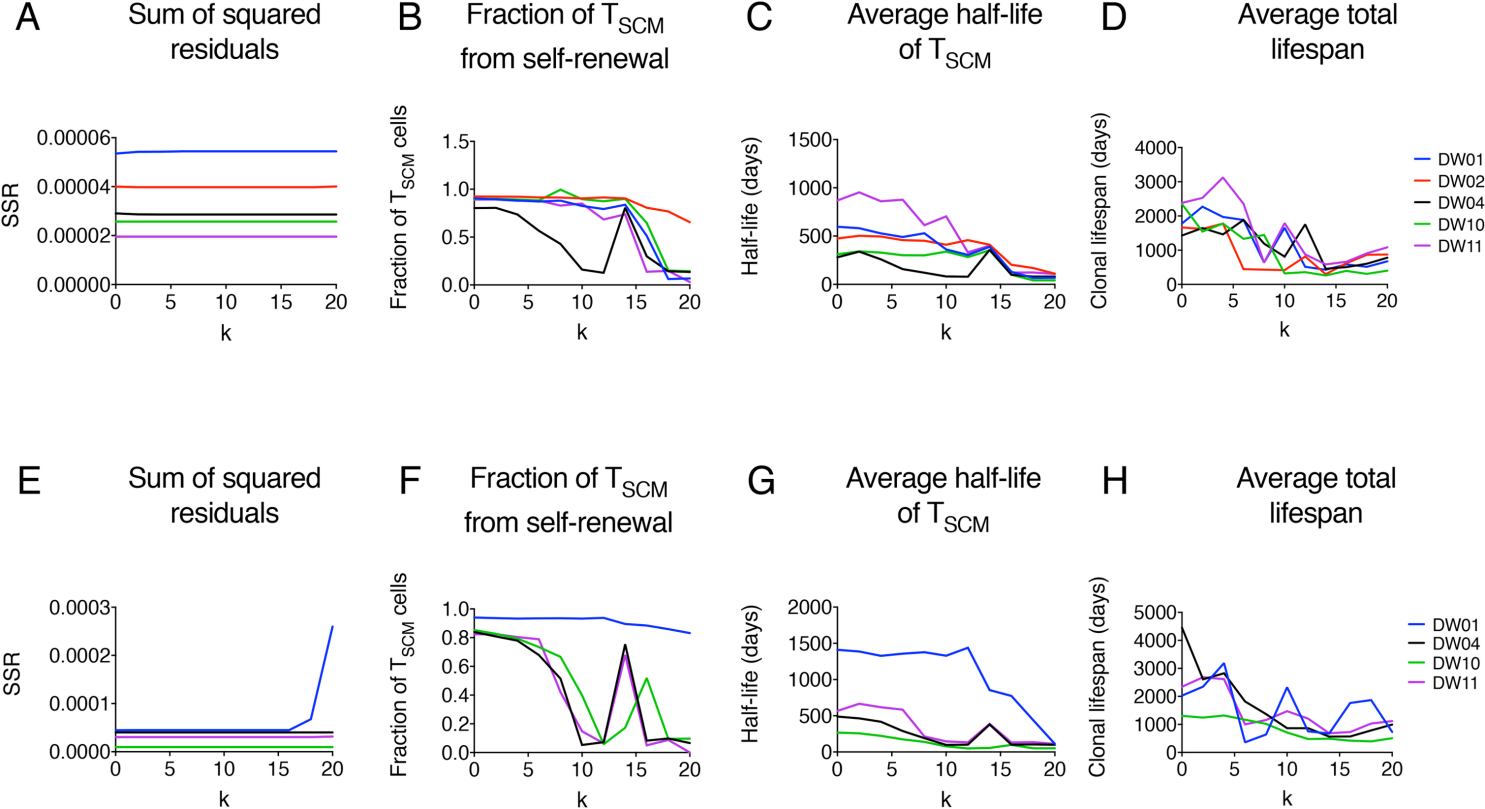
Estimates of T_SCM_ cell parameters from the implicit kinetic heterogeneity model. (A-H) Estimates of T_SCM_ cell parameters as a function of *k* in the CD4^+^ (top row: A—D) and CD8^+^ (bottom row: E—H) lineages. Isotope labelling and telomere length data were fitted simultaneously, fixing the clonal expansion size *k* to values between 0 and 20 and leaving the remaining parameters free. (A) and (E) show the variation in the sum of squared residuals (‘SSR’) with *k* in CD4^+^ and CD8^+^ T cells respectively; (B) and (F) show the variation in the fraction of newly generated T_SCM_ cells originating from self-renewing T_SCM_ proliferation computed as (*p*_*s*_T_SCM_) / (2^k^ΔT_N_ + *p*_*s*_T_SCM_); (C) and (G) show the variation in T_SCM_ half-lives for CD4^+^ and CD8^+^ T cells, respectively, and (D) and (H) the variation in T_SCM_ antigen-specific precursor lifespans (time until the last cell specific for a given antigen dies or differentiates). In all cases, *k* is plotted on the *x* axis. All results depicted are provided in S1 Data. T_SCM_, stem cell–like memory T.

The size of a newly generated T_SCM_ clone will be an important determinant of clonal longevity, as this determines not just the initial magnitude of a new clone but also the rate at which an existing clone is displaced by new entrants bearing different TCRs. Unfortunately, the size of the clonal expansion accompanying the differentiation of naïve to T_SCM_ cells (*k* in the model; Fig 2, Methods) was not identifiable. Different fitting runs to the same dataset (with different initial conditions or different random seeds) gave different estimates of the clonal expansion parameter *k*. Consistent with this, we found that if *k* was fixed to different constant values in the range 0–20, then, with the exception of 1 individual for which the sum of squares increases dramatically for *k* above 15, the sum of squares remained constant in every case for all values of *k* (Fig 4A and Fig 4E). Henceforth, we systematically repeat all analyses for multiple values of *k* in the range 0–20 to ensure that results are robust despite uncertainty in the clonal expansion parameter. Values of *k* above 20 were not considered biologically plausible [13, 32, 33].

In summary, we were not able to estimate the size of the clonal expansion *k* upon differentiation of naïve cells to T_SCM_ cells and instead utilised a strategy to investigate T_SCM_ dynamics despite uncertainty in this parameter.

### T_SCM_ clonal longevity

Next, we quantified T_SCM_ clonal longevity. We fitted the mathematical model (implicit heterogeneous version) simultaneously to the telomere length and isotope labelling data, with *k* fixed sequentially at different values in the range 0–20. We found that, with the exception of 1 dataset (CD8^+^ T cells in DW01), the contribution of naïve cells to T_SCM_ replacement was never less than 20% and could be as much as 90% (Fig 4B and 4F). Correspondingly, the average half-life of a T_SCM_ clone was short: the maximum ever observed (across all values of *k* and across all individuals) was 4 years, but typically, it was much shorter and in the range 0–500 days (Fig 4C and 4G). These conclusions about short clonal longevity were robust to assumptions regarding the activity of telomerase. Specifically, for all values of telomerase compensation considered in the range 0–*k*, the estimated average clonal half-lives were never higher than those estimates reported above (in which compensation was a free parameter). Importantly, this half-life represents the duration of memory to an antigen (Methods), not the conventional population half-life.

It is possible that a very small number of surviving T_SCM_ cells is sufficient to generate a substantial recall response, and so a short clonal half-life is not necessarily incompatible with long-lived recall responses. Moreover, extinction of some clones specific for a given antigen is not necessarily problematic if other clones (bearing different TCRs) specific for the same antigen survive. To assess this possibility, we used the exact Gillespie algorithm (Methods) to quantify the time for the last cell of an antigen-specific precursor population to disappear. This is reported as the precursor lifespan in Fig 4D and 4H. Whilst this did lead to a considerable increase in longevity (stochastic estimates of total antigen-specific precursor lifespan were typically 3 times longer than the deterministic clonal half-life), maximum estimates were still only of the order of 2,000 days (about 5 years) for most individuals.

In summary, although individual parameters were poorly identifiable, all parameter combinations able to describe the experimental data were associated with average clonal half-life estimates, which were much lower than the 8–15 year half-life of the recall response [3, 4]. Even total precursor lifespans (times until the last cell of an antigen-specific precursor population disappears) were lower than those values in most cases. We conclude that the average T_SCM_ population is replaced too rapidly for it to be the stem cell population responsible for maintaining memory.

### Subpopulation kinetics: YFV-specific responses

The model used up to this point allows for heterogeneity but nevertheless reports population averages (i.e., the proliferation rate and clonal half-life averaged across the whole T_SCM_ population). This averaging could be hiding a small, long-lived population within the bulk short-lived population. Expanding our model, which deals with heterogeneity implicitly (and thus averages across the population), to one that deals with heterogeneity explicitly (and thus provides estimates for the half-lives of all subpopulations) is problematic, as even the simplest version of the explicit heterogeneity model suffers from severe identifiability issues [30] and fails to deliver the parameters of interest when fitted to labelling data. We confirmed that, for our more complex system with both naïve and T_SCM_ cells, an explicit description of heterogeneity provided no information. To address this problem, we therefore sought an alternative class of data.

We analysed published data of the vaccine-induced YFV-specific T_SCM_ response in humans from Fuertes Marraco and colleagues [21]. In brief, the magnitude of the CD8^+^ T_SCM_ cell response to the HLA-A*02-restricted YFV nonstructural protein 4b (NS4b^214−222^) epitope was measured by HLA class I tetramer at different time points (range 0.27–35.02 years) postvaccination in a cross-sectional study of 37 recipients of the YF-17D YFV vaccine.

**Fig 5:**
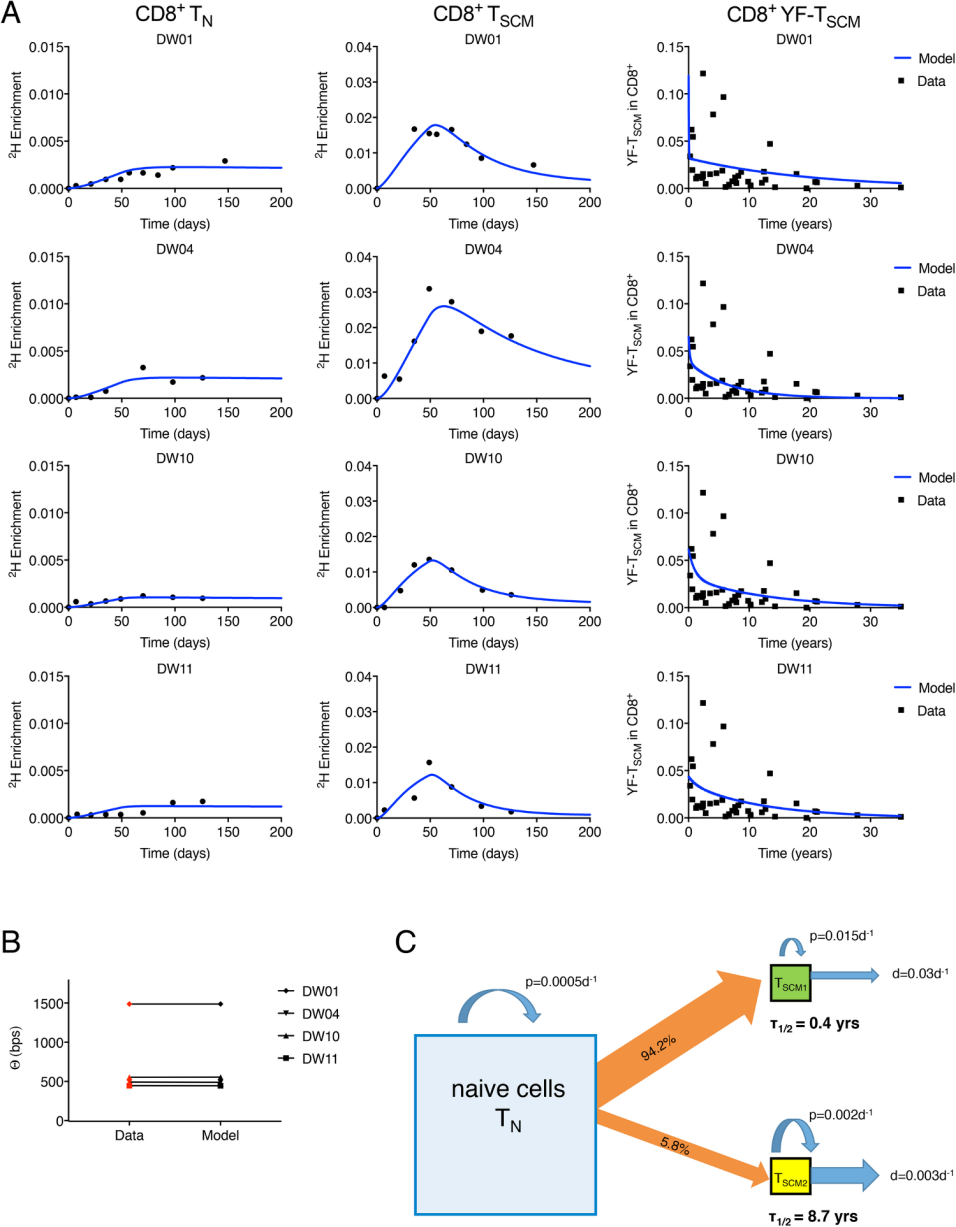
Label incorporation, telomere length, and YFV predictions for CD8^+^ T_N_ and T_SCM_ cells. (A) Fit of the explicit heterogeneity model to isotope labelling data from CD8^+^ T_N_ and T_SCM_ cells and to the YFV-specific T_SCM_ data. Experimental data are represented by solid black symbols. (B) fits to the average telomere length differences (*Θ*) between the CD8^+^ T_N_ and T_SCM_ pools, experimental data shown in red. The number of bps lost per division was taken to be δ = 50 bp/division. Isotope labelling, telomere length, and the YFV data were fitted simultaneously. (C) Schematic summary of estimated T_N_ and T_SCM_ dynamics. Estimates are the medians across subjects obtained from the fits shown in panels A and B. Parameter estimates for each subject are provided in Table 1 and S2 Table. The size of the squares is proportional to the population size. Labelling data and telomere data are provided in S1 Data; the YFV tetramer data were previously published [21]. bp, base pair; T_N_, naïve T; T_SCM_, stem cell–like memory T; YFV, yellow fever virus.

We fitted the explicit heterogeneity version of the naïve T (“T_N_”) and T_SCM_ model to all 3 types of CD8^+^ T cell data (isotope labelling, telomere length, and YFV) simultaneously (Supplementary Methods in S1 Text). The fits are shown in Fig 5. As for the implicit heterogeneity model, the fraction of new T_SCM_ cells originating from naïve cells was high (minimum 10%, median 44%). We found evidence for at least 2 subpopulations of CD8^+^ T_SCM_ cells (designated T_SCM1_ and T_SCM2_ for the purposes of this discussion). The majority of the T_SCM_ cells generated upon clonal expansion of naïve cells differentiated into the T_SCM1_ subpopulation, characterised by a short half-life (≤1 year) and a high replacement rate (median 0.02 per day, interquantile range 0.024–0.045 per day), slightly higher than the average rates estimated by the previous model. The remaining fraction of the generated clone was observed to enter a long-lived subpopulation with a median half-life of 9 years (Table 1, S2 Table). Surprisingly, although the fraction of naïve cells entering the T_SCM2_ pool was low, because of its low death/differentiation rate, the number of long-lived T_SCM2_ cells in the circulation at any given time could be as high as, or even higher than, the number of rapidly proliferating T_SCM1_ cells. Results are summarised schematically in Fig 5C. Four other weighting strategies (of the different types of data) yielded the same conclusions in all cases (S1 Fig, S3 Table).

**Table 1:** Parameter estimates for CD8^+^ T_SCM_ cells from the explicit heterogeneity model. Parameter estimates with 95% CIs (in parentheses) obtained by fitting the explicit heterogeneity model to isotope labelling, telomere length, and YFV datasets simultaneously. Table shows estimated half-life of the 2 subpopulations, the relative size of the long-lived T_SCM2_ subpopulation (T_SCM2_/T_SCM_), and the fraction of cells from each clonal burst that enter the T_SCM2_ subpopulation (*f*). Additional parameters are given in S2 Table.

	half-life T_SCM1_ [years]	half-life T_SCM2_ [years]	T_SCM2_/T_SCM [TOTAL]_	*f*
**DW01**	0.02 (95% CI 0.02–6.74)	13.92 (95% CI 2.26–20.68)	0.25 (95% CI 0.14–0.89)	5.6 × 10^−4^ (95% CI 7.6 × 10^−4^–7.2 × 10^−1^)
**DW04**	0.14 (95% CI 0.01–2.79)	4.59 (95% CI 2.13–20.41)	0.58 (95% CI 0.20–0.98)	4.1 × 10^−2^ (95% CI 2.0 × 10^−2^–7.6 × 10^−1^)
**DW10**	0.69 (95% CI 0.05–0.77)	9.09 (95% CI 2.33–16.50)	0.51 (95% CI 0.37–0.74)	7.4 × 10^−2^ (95% CI 1.9 × 10^−2^–1.5 × 10^−1^)
**DW11**	0.90 (95% CI 0.03–3.98)	8.39 (95% CI 3.75–17.01)	0.82 (95% CI 0.46–0.95)	3.3 × 10^−1^ (95% CI 2.1 × 10^−2^–7.3 × 10^−1^)
**MEDIAN**	0.41 (95% CI 0.02–3.39)	8.74 (95% CI 2.30–18.71)	0.55 (95% CI 0.28–0.92)	5.8 × 10^−2^ (95% CI 1.9 × 10^−2^–7.3 × 10^−1^)

**Abbreviations:** T_SCM_, stem cell–like memory T; YFV, yellow fever virus

### Long-lived CD8^+^ T_SCM_ cells: Degree of self-renewal and clonal stability

The long-lived T_SCM_ subpopulation identified in the previous section (Subpopulation kinetics) is a potential candidate for the stem cell population responsible for the maintenance of immune memory. We therefore investigated the degree of self-renewal and clonal stability within this long-lived T_SCM_ compartment. The degree of self-renewal of a population at steady state, 1 / (death rate + differentiation rate–proliferation rate), quantifies the upstream input necessary to maintain a population. If there is a large upstream contribution, then the degree of self-renewal will be low [34]. A perfectly self-renewing population (e.g., HSCs) will have an infinite degree of self-renewal. We quantified the degree of self-renewal for the long-lived population and found a median of 4,600 days, with the range 2,400–7,300 days (S2 Table). This implies that, on average, a T_SCM_ cell (or its progeny) from the long-lived subpopulation resides in the T_SCM_ compartment without dying or differentiating for 4,600 days.

**Fig 6:**
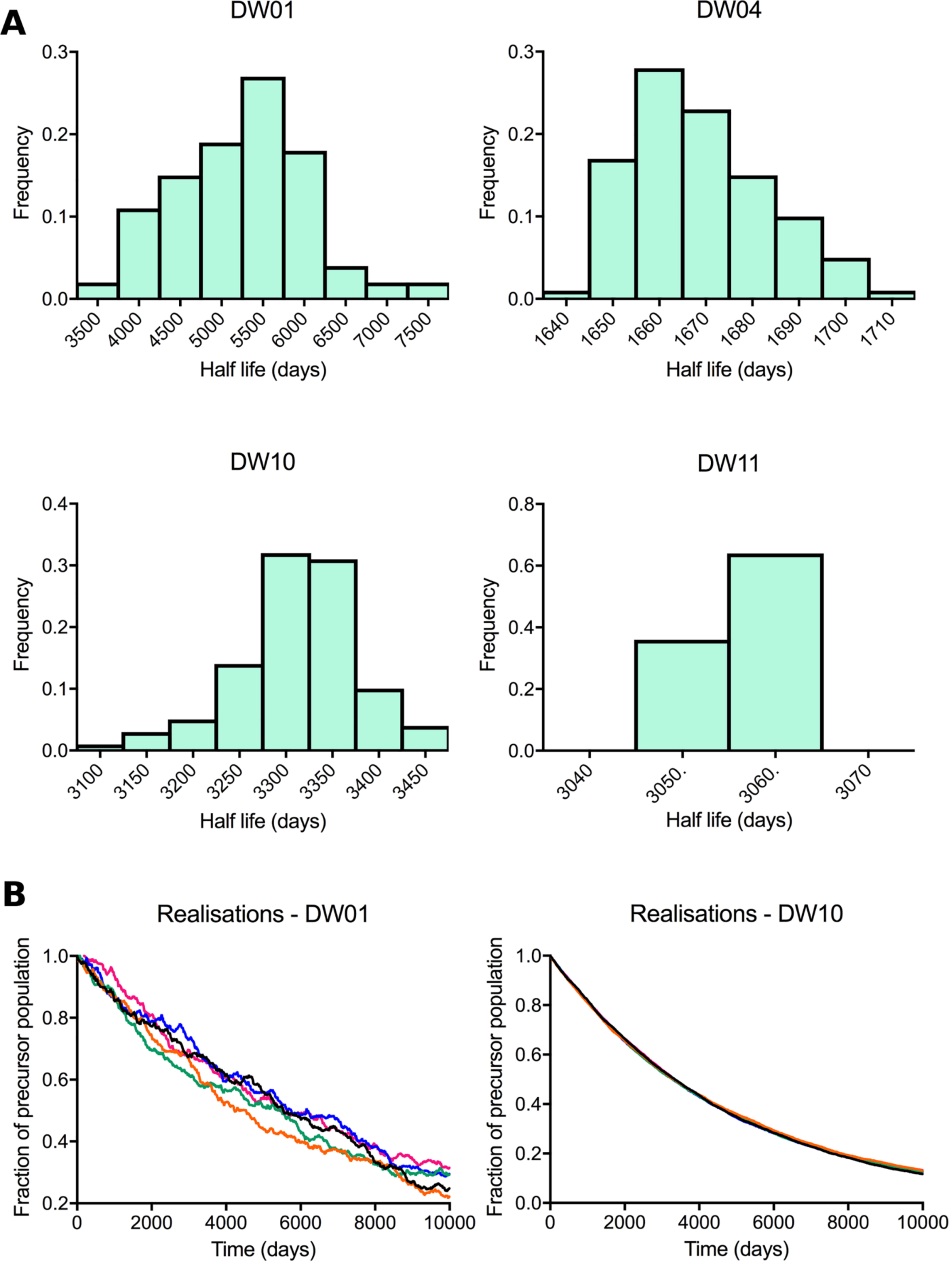
Stochastic dynamics of the long-lived antigen-specific T_SCM_ populations. Gillespie simulations of the change in size of antigen-specific precursor populations within the long-lived T_SCM_ pool were performed for each individual. (A) Distribution of antigen-specific precursor population half-lives. (B) Five randomly chosen Gillespie simulations for the 2 subjects exhibiting the most interrealisation variation (DW01 and DW10). Simulations were performed using the T_SCM_ parameters estimated by fitting the explicit heterogeneity model (Table 1 and S2 Table). All results depicted are provided in S1 Data. T_SCM_, stem cell–like memory T.

The total CD8^+^ T_SCM_ population is small (2%–3% of circulating CD8^+^ lymphocytes [19], 1%–5% of lymph node–resident CD8^+^ T cells [35]). If only a proportion of this already small population is responsible for maintaining memory, then this raises the issue that, although the precursor population specific for a given antigen may have a long half-life, the small size of that population could mean that its dynamics are highly stochastic. That is, there may be wide ranges in the length of memory, and some antigen-specific precursor populations would be predicted to be lost by stochastic extinction soon after generation—i.e., memory would be erratic and fallible. To investigate the stochasticity of the length of memory within the long-lived T_SCM_ pool, we performed Gillespie simulations of the size of the antigen-specific precursor population based on the parameter estimates derived from model fitting for each of the 4 individuals with CD8^+^ T cell data. Although there was stochasticity in the half-lives of antigen-specific precursors across different runs, the variation was not large (Fig 6A), and the different trajectories were tightly clustered (Fig 6B).

### Subpopulation kinetics: Compatibility with long half-lives between 5 and 15 years

Concerned that the YFV vaccine, which is known to generate an exceptional CD8^+^ T cell response, may not be representative of a typical antigen, we also sought to study T_SCM_ dynamics independent of the YFV dataset. Guided by the concept that there may be a long-lived T_SCM_ subpopulation, we fitted the explicit kinetic heterogeneity model to the isotope labelling and telomere length data, ignoring the YFV data but imposing a half-life greater than 5 years on the long-lived T_SCM_ subpopulation. As expected, parameters could no longer be reliably identified, but we were able to conclude that the dynamics of CD8^+^ T_SCM_ cells were compatible with long subpopulation half-lives between 5 and 15 years (S2 Fig). This approach also allowed us to study CD4^+^ T_SCM_ cells (which were not measured in the YFV study). Again, we found that the dynamics of CD4^+^ T_SCM_ cells were compatible with the presence of a similarly long-lived subpopulation with a half-life between 5 and 15 years (S3 Fig).

## Discussion

One leading explanation for the maintenance of long-term immunological memory is the existence of a stem cell–like population of memory T cells, able to both self-renew and to differentiate into all other subsets of the T cell memory pool [12, 17, 21]. It has been suggested that the recently discovered T_SCM_ population is the main stem cell population responsible for maintaining T cell memory [18, 36].

In a previous study, we used stable isotope labelling to investigate T_SCM_ dynamics at equilibrium in healthy subjects [26]. This revealed unexpectedly high rates of turnover in the CD4^+^ and CD8^+^ T_SCM_ compartments. Whilst supporting the concept that the T_SCM_ population as a whole is stable, it did not establish whether new T_SCM_ cells were generated from naïve cells or by T_SCM_ proliferation. Moreover, the key parameters of clonal longevity and self-renewal, which are prerequisites for stemness, could not be deconvoluted. Given that T_SCM_ cells were found to die and be replaced rapidly, the source of the replacing cell becomes critical. Even a small contribution from naïve cells can result in a weakly self-renewing population and loss of memory as existing clones are replaced by cells specific for a different antigen.

In the present study, we overcame these limitations by simultaneously fitting telomere length and tetramer data to constrain the space of possible models and improve parameter identifiability. We report that firstly, the T_SCM_ population is kinetically heterogeneous, with at least 2 kinetically distinct subpopulations turning over at different rates; and secondly, the dynamics of a subpopulation of T_SCM_ cells are compatible with their hypothesised role as the main stem cell–like T cell precursor responsible for the maintenance of T cell memory. The best description of the data is one in which the kinetically heterogeneous T_SCM_ population, despite its high average replenishment rate of approximately 2% per day, contained a fraction of long-lived T_SCM_ cells. The half-life of this slower subpopulation was approximately 9 years, consistent with the 8–15 year half-life estimated for the recall response to a given antigen [3, 4]. Furthermore, although this subpopulation was small, the dynamic behaviour of individual clones was not excessively stochastic, and the half-life of a given antigen-specific precursor population was tightly distributed. Finally, we estimated that the degree of self-renewal of the long-lived T_SCM_ subpopulation was approximately 4,600 days.

The quantification of the dynamics of T_SCM_ cells is not easily addressed in humans, and different studies invariably involve different compromises. An advantage of our study is that it examines the natural dynamics of T_SCM_ cells in healthy, lymphocyte-replete individuals. A disadvantage is that many of the model parameters were poorly identifiable; nevertheless, firm conclusions about clonal longevity and self-renewal can be drawn. A second disadvantage is that it utilises vaccination data generated using a vaccine (the YFV-17D vaccine) known for its ability to generate an exceptional CD8^+^ T cell response and which may not be representative of average immunity. To address this potential caveat, we repeated our analysis without reference to the YFV vaccination data. Whilst this reduced our ability to estimate individual parameters, it did confirm the conclusion that the T_SCM_ population consists of subpopulations with different kinetics and that high turnover and short half-lives of the bulk T_SCM_ population do not rule out the existence of a slowly turning over subpopulation with the dynamic properties required for T_SCM_ cells to maintain both CD4^+^ and CD8^+^ T cell memory. Finally, it should be noted that our study was confined to circulating T_SCM_ cells. In mice, it has been shown that memory T cell dynamics can vary across different anatomical compartments (spleen versus bone marrow) [37]. For human studies, ethical and technical considerations mean that repeated sampling of cells must be limited to the peripheral blood compartment. However, this does enable direct comparison with previously reported data, including the seminal description of human T_SCM_ cells [16], which was based on circulating cells.

Our findings of kinetic heterogeneity in the human T_SCM_ population are reminiscent of the proliferative heterogeneity described for transplanted HSCs in lethally irradiated mice [38], in which levels of Kit receptor distinguished cell subpopulations with different expansion capacities. Similarly, studies of human CD4^+^ and CD8^+^ T_SCM_ cells cultured in the presence of cytokines (either interluekin 15 [IL-15] or interluekin 7 [IL-7] + IL-15) in vitro have also reported that a fraction of cells proliferated rapidly, while the majority remained quiescent [39] [19]. Finally, in rhesus macaques, circulating T_SCM_ cells had a level of Ki-67 expression that the author remarked was ‘unexpectedly large’ (mean > 10%) [36]; this is consistent with our finding that, on average, the T_SCM_ population in peripheral blood turns over rapidly. Our proliferation rate estimates for naïve and T_SCM_ cells can be compared with proliferation rate estimates of other T cell subsets obtained using stable isotope labelling. We found that the proliferation of naïve T cells is slowest, *p* = 0.0005d^−1^, and comparable with previous estimates [40], though in this latter study, the gating strategy would have inadvertently included T_SCM_ cells in the naïve cell gate. Next is the slow T_SCM_ subpopulation with a proliferation rate an order of magnitude faster than naïve cells (*p* = 0.002d^−1^), then the fast T_SCM_ subpopulation with a median proliferation rate of *p* = 0.015d^−1^ comparable with that of memory cells (0.006d^−1^–0.02d^−1^ [29, 41]). Giving the following rank order of proliferation rates: naïve < slow T_SCM_ < fast T_SCM_ ≲ memory. Our estimate of the degree of self-renewal is more difficult to place in context. To the best of our knowledge, this parameter has only been quantified previously for murine HSCs. Our estimate of 4,600 days for the degree of self-renewal of the long-lived T_SCM_ subpopulation is naturally less than the corresponding estimate for murine HSC—which is, by definition, infinite—but is greater than the degree of self-renewal of compartments immediately adjacent to HSCs in the differentiation pathway—namely, short-term HSCs (degree of self-renewal of 90–150 days in mice) and multipotent progenitors (MPPs; degree of self-renewal of 7–28 days in mice) [23, 34]. If we convert to ‘animal lifespans’ (80 years for human, 2 years for mice; a scaling which appears valid for T cell kinetics [41]), then we see that the degree of self-renewal of T_SCM_ cells is 0.15 lives; i.e., a long-lived T_SCM_ cell resides without dying or differentiating for approximately 15% of our lifespan. This is similar to the degree of self-renewal of short-term HSCs (0.12–0.2 lives) and greater than the degree of self-renewal of MPPs (0.01–0.04 lives). It is remarkable that a peripheral cell population that is towards the end of the haematopoietic differentiation pathway should have a degree of self-renewal that is comparable with short-term HSCs.

Unexpectedly, we found strong evidence for continual differentiation of naïve T cells into the T_SCM_ cell pool despite the study volunteers being healthy with no symptomatic infection. For both the implicit and the explicit heterogeneity models, the contribution of naïve cells to T_SCM_ replacement was typically about 50% and never less than 10% (Fig 4B and 4F). This may represent differentiation of naïve cells in response to continual low-level exposure to novel environmental antigen and/or to persistent antigen. Considerable recruitment of naïve cells to the memory pool in the apparent absence of novel antigen has been previously described for mice persistently infected with Polyoma virus [42] or lymphocytic choriomeningitis virus [42] and for healthy mice [43].

The role of the short-lived T_SCM_ subpopulation that we identify is unclear. Potentially, it is activated naïve cells rapidly transitioning to effectors whilst others are retained to form the long-lived T_SCM_ pool that is the basis of memory. This is consistent with recent evidence that T_SCM_ cells may pass through a phase in which they express effector molecules [44].

This work suggests a number of future directions. One important direction is to establish phenotypic markers to distinguish the ‘true’ T_SCM_ subpopulation. A second is to develop a model to predict the TCR repertoire of the true T_SCM_ subpopulation and whether this differs from bulk T_SCM_ cells and the functional consequences of any such difference. Finally, it is important to know whether or not murine T_SCM_ populations are similarly heterogeneous, since this would facilitate a whole range of experiments not possible in humans.

Our results show that substantial kinetic heterogeneity exists within the T_SCM_ pool, encompassing a long-lived subpopulation with the dynamic properties required to maintain both CD4^+^ and CD8^+^ T cell memory. Further characterisation of these bona fide T_SCM_ cells may illuminate the mechanistic basis of durable immune protection and facilitate translational efforts to develop more effective vaccines and immunotherapies.

## Methods

### Experimental data

#### Ethics statement

Approval was granted by the Cardiff University School of Medicine and London-Chelsea Research Ethics Committees (REC: 13/LO/0022. IRAS: 109455). All studies were conducted according to the Principles of the Declaration of Helsinki, and all subjects gave written consent.

#### Study participants

Five healthy adults were studied (DW01, age 32; DW02, age 64; DW04, age 83; DW10, age 34; DW11, age 29). All subjects were CMV-seropositive and HIV-1-seronegative.

#### Stable isotope labelling in vivo

We have previously described the labelling protocol in detail [26]. Briefly, participants were given oral doses of 70% deuterated water (^2^H_2_O) over a 7-week period (50 ml 3 times daily for 1 week, then twice daily thereafter). Saliva samples were collected for evaluation of body water labelling. Venous blood was drawn at successive time points during and after labelling. Peripheral blood mononuclear cells were sorted at high purity, using a custom-modified BD FACSAria II flow cytometer, into CD4^+^ and CD8^+^ naïve and T_SCM_ cells on the basis of cell surface expression (naïve: CD45RO^―^CD27^bright^CCR7^+^CD95^―^ T_SCM_: CD45RO^―^CD27^bright^CCR7^+^CD95^+^). Both subsets were further assessed for expression of other cell surface markers; T_SCM_ cells were found to be CD45RA^+^, CD28^+^, CD127^+^, and CD57^―^ (Fig 1). Deuterium enrichment in the DNA of the sorted T cell subsets was measured by gas chromatography/mass spectrometry [45].

#### Single-chromosome telomere length analysis

DNA from CD4^+^ and CD8^+^ naïve and T_SCM_ cells (sorted as for stable isotope labelling analysis) was extracted, and single-telomere length analysis was carried out at the XpYp telomere as described previously [46].

#### Yellow fever vaccine data

Published data were acquired from a cross-sectional study of 37 healthy adults who received a single dose of the yellow fever vaccine YF-17D [21]. Time since vaccination ranged from 3 months to 35 years. Four subjects who received multiple YFV vaccinations were excluded from the analysis. CD8^+^CD45RA^+^CCR7^INT^ T_SCM_ cells specific for the HLA-A*02-restricted YFV NS4b^214-222^ epitope were quantified by tetramer staining and flow cytometry.

### Mathematical modelling

#### Homogeneous and implicit heterogeneous models

To study the dynamics of T_SCM_ clones, we developed an ODE model of the linear differentiation pathway between naïve and T_SCM_ cells (Fig 2). Nonlinear models of differentiation were not considered [47]. In the classical model of T_SCM_ formation, dedifferentiation of T_CM_, T_EFF_, and T_EM_ cells to T_SCM_ is infrequent [48–50], so we assumed a one-way differentiation pathway. Immunological memory has been shown to be generated by clonal expansion, in which naïve cells encountering antigen in lymphoid tissue undergo several rounds of division [1, 51–53]. We assume that newly generated T_SCM_ cells can arise in 2 ways: either they are the product of self-renewal (proliferation of T_SCM_ cells, *p*_*s*_), or they result from the differentiation of naïve cells following antigen exposure at a rate Δ. We assume that naïve and T_SCM_ cells are in constant recirculation between lymph and blood [15, 19], giving the following equations:

$${\dot{T}}_{N}={(p}_{n}-d_{n}-\Delta )T_{N}$$ (1)

$${\dot{T}}_{SCM}=\Delta 2^{k}T_{N}+{(p}_{s}-d_{s})T_{SCM},$$ (2)

in which *T*_*N*_ and *T*_*SCM*_ are the total number of cells in the naïve and T_SCM_ populations, respectively; *p*_*n*_, *d*_*n*_, *p*_*s*_, and *d*_*s*_ the proliferation and disappearance rates of naïve and T_SCM_ cells, respectively; *Δ* is the fraction of naïve cells activated by antigen exposure per day; and *k* is the number of divisions occurring during clonal expansion in the differentiation from naïve to T_SCM_ cells (resulting in 2^k^ T_SCM_ cells generated from each naïve cell). A value of *k* equal to 1 indicates that naïve cells divide only once after priming, and a value of *k* equal to 0 indicates that no divisions occur after antigen exposure. Deuterium-labelling experiments measure the fraction of deoxyadenosine nucleosides (dAs) with incorporated deuterium, so we construct the model in terms of dAs. The absolute numbers of labelled dAs derived from Eqs 1 and 2 are

$${\dot{T}}_{N}^{*}=p_{n}cU(t)T_{N}-(d_{n}^{*}+\Delta )T_{N}^{*}$$ (3)

$${\dot{T}}_{SCM}^{*}=\Delta (2^{k}-1){cU(t)T}_{N}+\Delta T_{N}^{*}+p_{s}cU(t)T_{SCM}-d_{s}^{*}T_{SCM}^{*},$$ (4)

in which $T_{N}^{*}$ and $T_{SCM}^{*}$ represent the absolute numbers of labelled dAs from naïve and T_SCM_ cells, respectively; $d_{n}^{*}$ and $d_{s}^{*}$ are the disappearance rates of labelled naïve and T_SCM_ cells, respectively; *c* is the amplification factor for enrichment; and *U(t)* is an empirical function describing label availability as measured in saliva [40]:

$$U(t)=f_{r}(1-e^{-\delta t})+\beta e^{-\delta t}$$ (5)

$$U(t)=[f_{r}(1-e^{-\delta \tau })+\beta e^{-\delta \tau }]e^{-\delta (t-\tau )},$$ (6)

in which *τ* represents the time at which the administration of label is stopped, *f*_*r*_ represents the fraction of deuterium in water, *δ* the turnover rate per day of body water, and β the baseline saliva enrichment. Parameters *f*_*r*_, β, and *δ* are known to vary between individuals, and their values were obtained by fitting Eqs 5 and 6 to successive measurements of label enrichment in saliva from each subject. The measured body water enrichments with best fits are shown in S4 Fig; estimates of *f*_*r*_, β, and *δ* are provided in S4 Table. The equations for the fraction of labelled dAs are then

$${\dot{F}}_{TN}=p_{n}cU(t)-(d_{n}^{*}+\Delta )F_{TN}$$ (7)

$${\dot{F}}_{TSCM}=(2^{k}-1)cU(t)\frac{\Delta T_{N}}{T_{SCM}}+\frac{\Delta T_{N}}{T_{SCM}}F_{TN}+p_{s}cU(t)-d_{s}^{*}F_{TSCM}.$$ (8)

While the naïve pool has been observed to be kinetically homogeneous [40], the heterogeneity of the T_SCM_ population has not yet been explored. We have previously argued [28] that if a cell population is kinetically heterogeneous, the rate of label uptake during a labelling experiment will not be equal to the rate at which the label is lost. This scenario arises because the labelled population (and therefore the disappearance rate estimated from it) will not be representative of the whole population, as subpopulations with faster kinetics divide faster and will be overrepresented in the labelled cells. We can therefore impose kinetic homogeneity on the T_SCM_ pool (‘homogenous model’) by constraining $d_{s}^{*}=d_{s}$ where 2^k^ΔT_N_ + p_s_T_SCM_ = d_s_T_SCM_. Removal of this constraint is equivalent to allowing the T_SCM_ pool to be heterogeneous (‘implicit heterogeneity model’). Assuming kinetic homogeneity in the naïve pool, the equations for the fractions of labelled DNA at steady state are therefore

$${\dot{F}}_{TN}=p_{n}(cU(t)-F_{TN})$$ (9)

$${\dot{F}}_{TSCM}=\frac{\Delta T_{N}}{T_{SCM}}((2^{k}-1)cU(t)+F_{TN})+p_{s}cU(t)-d_{s}^{*}F_{TSCM},$$ (10)

in which $d_{s}^{*}=d_{s}$ yields the homogeneous version of the model, and, if $d_{s}^{*}$ is free, then this yields the implicit heterogeneity version of the model. We also tested a version of the model in which the assumption of homogeneity for naïve cells (*d*_n_* = d_*n*_) was relaxed.

#### The explicit heterogeneous model

The implicit heterogeneous model (Eqs (9) and (10)) describes the average population dynamics of a heterogeneous T_SCM_ pool. However, to estimate the sizes, the proliferation, and the disappearance rates of each of the T_SCM_ subpopulations, these subpopulations need to be modelled explicitly. The explicit heterogeneous model describes 2 kinetically distinct subpopulations of T_SCM_ cells. Additional subpopulations could not be resolved with the available data. If there are more than 2 subpopulations with distinct kinetics, then the subpopulation 1 and subpopulation 2 that we measure will have kinetics that represent the average of their smaller constituent subpopulations. The equations for the total numbers of cells in each of the modelled pools are

$${\dot{T}}_{N}={(p}_{n}-d_{n}-\Delta )T_{N}$$ (11)

$${\dot{T}}_{SCM1}=\Delta (1-f)2^{k}T_{N}+{(p}_{s1}-d_{s1})T_{SCM1}$$ (12)

$${\dot{T}}_{SCM2}=\Delta f2^{k}T_{N}+{(p}_{s2}-d_{s2})T_{SCM2},$$ (13)

in which *f* is the proportion of cells from the clonal burst (of size *2*^*k*^) that differentiate into the second T_SCM_ subpopulation; *p*_*s*1_, *d*_*s*1_, *p*_*s*2_ and *d*_*s*2_ are the proliferation and disappearance rates of T_SCM1_ and T_SCM2_ cells, respectively; and the remaining parameters are as described for Eqs (3) and (4). The absolute number of labelled dAs derived from Eqs 11, 12, and 13 are

$${\dot{T}}_{N}^{*}=p_{n}cU(t)T_{N}-(d_{n}+\Delta )T_{N}^{*}$$ (14)

$${\dot{T}}_{SCM1}^{*}=\Delta (1-f)(2^{k}-1)cU(t)T_{N}+\Delta (1-f)T_{N}^{*}+p_{s1}cU(t)T_{SCM1}-d_{s1}T_{SCM1}^{*}$$ (15)

$${\dot{T}}_{SCM1}^{*}=\Delta f(2^{k}-1)cU(t)T_{N}+\Delta fT_{N}^{*}+p_{s2}cU(t)T_{SCM2}-d_{s2}T_{SCM2}^{*},$$ (16)

and the fractions of labelled DNA assuming steady state for cell population sizes are

$${\dot{F}}_{TN}=p_{n}(cU(t)-F_{TN})$$ (17)

$${\dot{F}}_{TSCM1}=\Delta (1-f)\frac{T_{N}}{T_{SCM1}}((2^{k}-1)cU(t)+F_{TN}-2^{k}F_{TSCM1})+p_{s1}(cU(t)-F_{TSCM1})$$ (18)

$${\dot{F}}_{TSCM2}=\Delta f\frac{T_{N}}{T_{SCM2}}\left((2^{k}-1)cU(t)+F_{TN}-2^{k}F_{TSCM2}\right)+p_{s2}(cU(t)-F_{TSCM2}).$$ (19)

#### Telomere length model (for homogeneous and implicit heterogeneity models)

Following de Boer and Noest [54], we derived an ODE model to describe the progressive shortening with division of the average telomere lengths in the naïve and T_SCM_ cell populations (Fig 2B) for the homogenous and implicit heterogeneity models. The equivalent derivation for the explicit heterogeneity model follows the same pattern and is presented in the SI.

T_N_ and T_SCM_ cell populations were modelled as a series of *n* compartments, with T_Ni_ (or T_SCMi_) representing the number of T_N_ (or T_SCM_) cells that have divided *i* times. If *δ* is the number of base pairs lost per cell division, then the cells in T_Ni_ (or T_SCMi_) have decreased their telomere lengths by *δi* base pairs. From Eqs (1) and (2) above, the number of cells in the T_Ni_ and T_SCMi_ compartments are

$${\dot{T}}_{N_{i}}=2p_{n}T_{N_{i-1}}-(p_{n}+\Delta +d_{n})T_{N_{i}}$$ (20)

$${\dot{T}}_{{SCM}_{i}}=2p_{s}T_{{SCM}_{i-1}}-(p_{s}+d_{s})T_{{SCM}_{i}}+{\Delta 2}^{k}T_{N_{i-C}},$$ (21)

in which *p*_*n*_, Δ, *d*_*n*_, *p*_*s*_, *d*_*s*_, and *k* are as in Eqs 1 and 2, and *C* is a parameter to allow for the impact of telomerase. If *C =* 0, then no shortening of telomeres occurs during clonal expansion (total compensation by telomerase); if *C* = *k*, then there is no compensation by telomerase. Experimental evidence suggests that, both for HSCs and peripheral T cells, telomerase attenuates, but does not prevent, telomere loss [55], i.e., 0 < *C* < *k*. The equations for the average number of divisions undergone by the cells in the naïve and the T_SCM_ pools in our model (Eqs 20 and 21) follow trivially from the derivation in [54] and are given by

$${\dot{\mu }}_{TN}=2p_{n}$$ (22)

$${\dot{\mu }}_{TSCM}=2p_{s}-{\Delta 2}^{k}\frac{T_{N}}{T_{SCM}}(\mu_{T_{SCM}}-\mu_{T_{N}}-C).$$ (23)

Average telomere lengths are obtained by multiplying *μ*_*TN*_ and *μ*_*TSCM*_ by δ. We define $\Theta ≔\delta (\mu_{T_{SCM}}-\mu_{T_{N}}),$ the difference between the average telomere lengths in the T_N_ and the T_SCM_ pool (in units of base pairs). The dynamics of Θ are then given by

$$\dot{\Theta }=2(p_{s}-p_{n})\delta -{\Delta 2}^{k}\frac{T_{N}}{T_{SCM}}(\Theta -C\delta ).$$ (24)

Finally, when Eq 24 reaches steady state (shown numerically to have occurred for all subjects), then

$$\Theta =C\delta +\frac{(p_{s}-p_{n}){\delta T}_{SCM}}{2^{\left(k-1\right)}\Delta T_{N}}.$$ (25)

We use an estimate of δ = 50 bp/division, within the reported range of 35–70 bp/division [54]. Including the telomere data helps to constrain the model fit by placing a bound on the maximum number of divisions occurring between an average naïve T cell and an average T_SCM_ cell.

#### Computing clonal lifespans and half-lives

We computed the half-life of a clone deterministically from the T_SCM_ parameters estimated by fitting the ODE models. We are interested in the half-life of the memory (rather than the classically reported population half-life), and so this is defined as

$$\tau_{1/2}=\frac{\ln(2)}{d_{s}-p_{s}}.$$ (26)

Antigen-specific precursor lifespans (time until the last cell of an antigen-specific memory precursor population disappears) were computed stochastically using the exact Gillespie algorithm [56, 57]. At each step of the algorithm, either a division or a disappearance event is chosen, with respective probabilities of *x*(*t*)*p*/*S*(*t*) and *x*(*t*)*d*/*S*(*t*), in which *p* and *d* are the proliferation and disappearance rates of the T_SCM_ population; *x(t)* is population size at time *t*; and *S(t)* is the sum of *x(t)p* and *x(t)d*. At the end of each step, time *t* is incremented by a number of days sampled from an exponential distribution with rate *S(t)*. As the naïve T cell population in an adult human has an approximate size of 10^11^ cells [58], we estimate the initial size of a T_SCM_ clone in the long-lived subpopulation as

$${size}_{0}=10^{11}\times 2^{k}\times \Delta \times f,$$ (27)

in which *k* is the number of divisions that occur during clonal expansion in the differentiation from T_N_ to T_SCM_, Δ is the fraction of naïve cells activated by the same antigen, and *f* is the fraction of a newly generated clone that enters the long-lived pool. Antigen-specific T cell precursor frequency has been estimated in the naïve cell pool at <1 to 352 per 10^5^ naïve CD8^+^ cells [59]. For estimates of precursor lifespan in the implicit heterogeneity model, we fixed Δ to a representative value of 1 × 10^−5^ (and as we are considering average lifespan, *f* = 1). For estimates of precursor lifespan of the long-lived T_SCM_ subpopulation obtained using the explicit heterogeneity model, the values of Δ and *f* estimated by model fitting were used. Calculations of the initial size of a long-lived T_SCM_ clone are provided in S5 Table.

#### Model of the frequency of YFV-specific CD8^+^ T_SCM_ cells

The proportion of tetramer^+^ T_SCM_ cells expressed as a fraction of CD8^+^ CD16^−^ lymphocytes as a function of time since vaccination was modelled, allowing for 2 kinetically distinct subpopulations with exponential decay kinetics:

$$F=re^{-\alpha t}+(1-r)e^{-\beta t}.$$ (28)

#### Degree of self-renewal

The degree of self-renewal of the long-lived T_SCM_ subpopulation (T_SCM2_) is defined as

$$\begin{array}{c} \mathrm{degree}\;\mathrm{of}\;\mathrm{self}‑\mathrm{renewal}=\frac{1}{d_{s2}-p_{s2}}\\ \;=\frac{T_{SCM2}}{\Delta 2^{k}fT_{N}} \end{array}$$ (29)

#### Fitting procedures

Models were fitted to the experimental data by minimizing the sum of squared residuals, using the pseudoOptim algorithm from the FME package in R [60, 61]. Details of the fitting procedure, the different models, data fitted, and rationale are summarised in the Supplementary Methods in S1 Text.

#### Script availability

The scripts used for fitting the homogenous model, the implicit heterogeneous model, and the explicit heterogeneous model, as well as the script for running the Gillespie simulation, are all freely available at Zenodo DOI 10.5281/zenodo.1253178 [27].

### Statistics

The fit of the homogenous and heterogeneous model was compared using Fisher’s F-test for nested models. This test compares the goodness of fit of nested models to data, taking into account the different number of parameters in the models [62].

## Supporting information

S1 Fig (PDF)

S2 Fig (PDF)

S3 Fig (PDF)

S4 Fig (PDF)

S1 Table (PDF)

S2 Table (PDF)

S3 Table (PDF)

S4 Table (PDF)

S1 Text (PDF)

S1 Data (PDF)

## Abbreviations

CCR7: C-C chemokine receptor 7
dA: deoxyadenosine nucleoside
FSC-A: forward scatter area
FSC-H: forward scatter height
HSC: haematopoietic stem cell
IL-7: interleukin 7
IL-7Rα: interleukin 7 receptor alpha
IL-15: interleukin 15
MPP: multipotent progenitor
NS4b: nonstructural protein 4b
ODE: ordinary differential equation
SSC-A: side scatter area
T_CM_: central memory T
TCR: T cell receptor
T_EFF_: T effector
T_EM_: effector memory T
T_SCM_: stem cell–like memory T
YFV: yellow fever virus

## References

[1] Farber DL, Yudanin NA, Restifo NP. Human memory T cells: generation, compartmentalization and homeostasis. Nat Rev Immunol. 2014;14(1):24–35. doi: 10.1038/nri3567 24336101 10.1038/nri3567 PMC4032067

[2] Ahmed R, Gray D. Immunological memory and protective immunity: understanding their relation. Science. 1996;272(5258):54–60. 8600537 10.1126/science.272.5258.54

[3] Crotty S, Felgner P, Davies H, Glidewell J, Villarreal L, Ahmed R. Cutting edge: long-term B cell memory in humans after smallpox vaccination. J Immunol. 2003;171(10):4969–73. 14607890 10.4049/jimmunol.171.10.4969

[4] Hammarlund E, Lewis MW, Hansen SG, Strelow LI, Nelson JA, Sexton GJ, et al Duration of antiviral immunity after smallpox vaccination. Nat Med. 2003;9(9):1131–7. doi: 10.1038/nm917 12925846 10.1038/nm917

[5] Fearon DT, Manders P, Wagner SD. Arrested differentiation, the self-renewing memory lymphocyte, and vaccination. Science. 2001;293(5528):248–50. doi: 10.1126/science.1062589 11452114 10.1126/science.1062589

[6] Macallan DC, Borghans JA, Asquith B. Human T Cell Memory: A Dynamic View. Vaccines. 2017;5(1):E5–57. doi: 10.3390/vaccines5010005 28165397 10.3390/vaccines5010005 PMC5371741

[7] Mackay LK, Stock AT, Ma JZ, Jones CM, Kent SJ, Mueller SN, et al Long-lived epithelial immunity by tissue-resident memory T (TRM) cells in the absence of persisting local antigen presentation. Proceedings of the National Academy of Sciences of the United States of America. 2012;109(18):7037–42. doi: 10.1073/pnas.1202288109 22509047 10.1073/pnas.1202288109 PMC3344960

[8] Jiang X, Clark RA, Liu L, Wagers AJ, Fuhlbrigge RC, Kupper TS. Skin infection generates non-migratory memory CD8+ T(RM) cells providing global skin immunity. Nature. 2012;483(7388):227–31. doi: 10.1038/nature10851 22388819 10.1038/nature10851 PMC3437663

[9] Di Rosa F. Maintenance of memory T cells in the bone marrow: survival or homeostatic proliferation? Nat Rev Immunol. 2016;16(4):271. 10.1038/nri.2016.31 26996200

[10] Sallusto F, Geginat J, Lanzavecchia A. Central memory and effector memory T cell subsets: function, generation, and maintenance. Annu Rev Immunol. 2004;22:745–63. doi: 10.1146/annurev.immunol.22.012703.104702 15032595 10.1146/annurev.immunol.22.012703.104702

[11] Stemberger C, Neuenhahn M, Gebhardt FE, Schiemann M, Buchholz VR, Busch DH. Stem cell-like plasticity of naive and distinct memory CD8+ T cell subsets. Semin Immunol. 2009;21(2):62–8. doi: 10.1016/j.smim.2009.02.004 19269852 10.1016/j.smim.2009.02.004

[12] Graef P, Buchholz VR, Stemberger C, Flossdorf M, Henkel L, Schiemann M, et al Serial transfer of single-cell-derived immunocompetence reveals stemness of CD8(+) central memory T cells. Immunity. 2014;41(1):116–26. doi: 10.1016/j.immuni.2014.05.018 25035956 10.1016/j.immuni.2014.05.018

[13] Gerlach C, Rohr JC, Perie L, van Rooij N, van Heijst JW, Velds A, et al Heterogeneous differentiation patterns of individual CD8+ T cells. Science. 2013;340(6132):635–9. 23493421 10.1126/science.1235487

[14] Zhang Y, Joe G, Hexner E, Zhu J, Emerson SG. Host-reactive CD8+ memory stem cells in graft-versus-host disease. Nat Med. 2005;11(12):1299–305. doi: 10.1038/nm1326 16288282 10.1038/nm1326

[15] Lugli E, Gattinoni L, Roberto A, Mavilio D, Price DA, Restifo NP, et al Identification, isolation and in vitro expansion of human and nonhuman primate T stem cell memory cells. Nature Protocols. 2013;8(1):33–42. doi: 10.1038/nprot.2012.143 23222456 10.1038/nprot.2012.143 PMC6328292

[16] Gattinoni L, Lugli E, Ji Y, Pos Z, Paulos CM, Quigley MF, et al A human memory T cell subset with stem cell-like properties. Nature Medicine. 2011;17(10):1290–7. doi: 10.1038/nm.2446 21926977 10.1038/nm.2446 PMC3192229

[17] Biasco L, Scala S, Basso Ricci L, Dionisio F, Baricordi C, Calabria A, et al In vivo tracking of T cells in humans unveils decade-long survival and activity of genetically modified T memory stem cells. Sci Transl Med. 2015;7(273):273ra13 doi: 10.1126/scitranslmed.3010314 25653219 10.1126/scitranslmed.3010314

[18] Gattinoni L, Speiser DE, Lichterfeld M, Bonini C. T memory stem cells in health and disease. Nat Med. 2017;23(1):18–27. doi: 10.1038/nm.4241 28060797 10.1038/nm.4241 PMC6354775

[19] Gattinoni L, Lugli E, Ji Y, Pos Z, Paulos CM, Quigley MF, et al A human memory T cell subset with stem cell-like properties. Nat Med. 2011;17(10):1290–7. doi: 10.1038/nm.2446 21926977 10.1038/nm.2446 PMC3192229

[20] Fisman DN, Savage R, Gubbay J, Achonu C, Akwar H, Farrell DJ, et al Older age and a reduced likelihood of 2009 H1N1 virus infection. N Engl J Med. 2009;361(20):2000–1. doi: 10.1056/NEJMc0907256 19907052 10.1056/NEJMc0907256

[21] Fuertes Marraco SA, Soneson C, Cagnon L, Gannon PO, Allard M, Abed Maillard S, et al Long-lasting stem cell-like memory CD8+ T cells with a naive-like profile upon yellow fever vaccination. Sci Transl Med. 2015;7(282):282ra48 doi: 10.1126/scitranslmed.aaa3700 25855494 10.1126/scitranslmed.aaa3700

[22] Oliveira G, Ruggiero E, Stanghellini MT, Cieri N, D'Agostino M, Fronza R, et al Tracking genetically engineered lymphocytes long-term reveals the dynamics of T cell immunological memory. Sci Transl Med. 2015;7(317):317ra198 doi: 10.1126/scitranslmed.aac8265 26659572 10.1126/scitranslmed.aac8265

[23] Busch K, Klapproth K, Barile M, Flossdorf M, Holland-Letz T, Schlenner SM, et al Fundamental properties of unperturbed haematopoiesis from stem cells in vivo. Nature. 2015;518(7540):542–6. doi: 10.1038/nature14242 25686605 10.1038/nature14242

[24] Sun J, Ramos A, Chapman B, Johnnidis JB, Le L, Ho Y-J, et al Clonal dynamics of native haematopoiesis. Nature. 2014;514(7522):322–7. doi: 10.1038/nature13824 25296256 10.1038/nature13824 PMC4408613

[25] Sawen P, Lang S, Mandal P, Rossi DJ, Soneji S, Bryder D. Mitotic History Reveals Distinct Stem Cell Populations and Their Contributions to Hematopoiesis. Cell Reports. 2016;14(12):2809–18. doi: 10.1016/j.celrep.2016.02.073 26997272 10.1016/j.celrep.2016.02.073 PMC4819906

[26] Ahmed R, Roger L, Costa Del Amo P, Miners KL, Jones RE, Boelen L, et al Human Stem Cell-like Memory T Cells Are Maintained in a State of Dynamic Flux. Cell Reports. 2016;17(11):2811–8. doi: 10.1016/j.celrep.2016.11.037 27974195 10.1016/j.celrep.2016.11.037 PMC5186732

[27] Costa Del Amo P. Scripts from "Human TSCM cell dynamics in vivo are compatible with long-lived immunological memory and stemness". Openly available from Zenodo DOI 105281/zenodo1253178. 2018. 10.1371/journal.pbio.2005523 PMC6033534 29933397

[28] Asquith B, Debacq C, Macallan DC, Willems L, Bangham CR. Lymphocyte kinetics: the interpretation of labelling data. Trends in Immunology. 2002;23(12):596–601. 12464572 10.1016/s1471-4906(02)02337-2

[29] Macallan DC, Asquith B, Irvine AJ, Wallace DL, Worth A, Ghattas H, et al Measurement and modeling of human T cell kinetics. European Journal of Immunology. 2003;33(8):2316–26. doi: 10.1002/eji.200323763 12884307 10.1002/eji.200323763

[30] Ganusov VV, Borghans JA, De Boer RJ. Explicit kinetic heterogeneity: mathematical models for interpretation of deuterium labeling of heterogeneous cell populations. PLoS Comput Biol. 2010;6(2):e1000666 doi: 10.1371/journal.pcbi.1000666 20140186 10.1371/journal.pcbi.1000666 PMC2816685

[31] De Boer RJ, Perelson AS, Ribeiro RM. Modelling deuterium labelling of lymphocytes with temporal and/or kinetic heterogeneity. Journal of the Royal Society, Interface / the Royal Society. 2012;9(74):2191–200. 10.1098/rsif.2012.0149 PMC3405764 22513720

[32] Buchholz VR, Flossdorf M, Hensel I, Kretschmer L, Weissbrich B, Graf P, et al Disparate individual fates compose robust CD8+ T cell immunity. Science. 2013;340(6132):630–5. 23493420 10.1126/science.1235454

[33] Kaech SM, Ahmed R. Memory CD8+ T cell differentiation: initial antigen encounter triggers a developmental program in naive cells. Nature Immunology. 2001;2(5):415–22. doi: 10.1038/87720 11323695 10.1038/87720 PMC3760150

[34] Hofer T, Busch K, Klapproth K, Rodewald HR. Fate Mapping and Quantitation of Hematopoiesis In Vivo. Annual Review of Immunology. 2016;34:449–78. doi: 10.1146/annurev-immunol-032414-112019 27168243 10.1146/annurev-immunol-032414-112019

[35] Hong H, Gu Y, Sheng SY, Lu CG, Zou JY, Wu CY. The Distribution of Human Stem Cell-like Memory T Cell in Lung Cancer. Journal of Immunotherapy. 2016;39(6):233–40. 27244531 10.1097/CJI.0000000000000128 PMC4902324

[36] Lugli E, Dominguez MH, Gattinoni L, Chattopadhyay PK, Bolton DL, Song K, et al Superior T memory stem cell persistence supports long-lived T cell memory. The Journal of Clinical Investigation. 2013;123(2):594–9. doi: 10.1172/JCI66327 23281401 10.1172/JCI66327 PMC3561805

[37] Siracusa F, Alp OS, Maschmeyer P, McGrath M, Mashreghi MF, Hojyo S, et al Maintenance of CD8(+) memory T lymphocytes in the spleen but not in the bone marrow is dependent on proliferation. European Journal of Immunology. 2017;47(11):1900–5. doi: 10.1002/eji.201747063 28815584 10.1002/eji.201747063 PMC5698754

[38] Grinenko T, Arndt K, Portz M, Mende N, Gunther M, Cosgun KN, et al Clonal expansion capacity defines two consecutive developmental stages of long-term hematopoietic stem cells. The Journal of Experimental Medicine. 2014;211(2):209–15. doi: 10.1084/jem.20131115 24446490 10.1084/jem.20131115 PMC3920556

[39] Abdelsamed HA, Moustaki A, Fan Y, Dogra P, Ghoneim HE, Zebley CC, et al Human memory CD8 T cell effector potential is epigenetically preserved during in vivo homeostasis. The Journal of Experimental Medicine. 2017;214(6):1593–606. doi: 10.1084/jem.20161760 28490440 10.1084/jem.20161760 PMC5461005

[40] Vrisekoop N, den Braber I, de Boer AB, Ruiter AF, Ackermans MT, van der Crabben SN, et al Sparse production but preferential incorporation of recently produced naive T cells in the human peripheral pool. Proc Natl Acad Sci U S A. 2008;105(16):6115–20. doi: 10.1073/pnas.0709713105 18420820 10.1073/pnas.0709713105 PMC2329696

[41] Westera L, Drylewicz J, den Braber I, Mugwagwa T, van der Maas I, Kwast L, et al Closing the gap between T-cell life span estimates from stable isotope-labeling studies in mice and humans. Blood. 2013;122(13):2205–12. doi: 10.1182/blood-2013-03-488411 23945154 10.1182/blood-2013-03-488411

[42] Vezys V, Masopust D, Kemball CC, Barber DL, O'Mara LA, Larsen CP, et al Continuous recruitment of naive T cells contributes to heterogeneity of antiviral CD8 T cells during persistent infection. The Journal of Experimental Medicine. 2006;203(10):2263–9. doi: 10.1084/jem.20060995 16966427 10.1084/jem.20060995 PMC2118117

[43] Gossel G, Hogan T, Cownden D, Seddon B, Yates AJ. Memory CD4 T cell subsets are kinetically heterogeneous and replenished from naive T cells at high levels. eLife. 2017;6. 10.7554/eLife.23013 PMC5426903 28282024

[44] Akondy RS, Fitch M, Edupuganti S, Yang S, Kissick HT, Li KW, et al Origin and differentiation of human memory CD8 T cells after vaccination. Nature. 2017. 10.1038/nature24633 PMC6037316 29236685

[45] Busch R, Neese RA, Awada M, Hayes GM, Hellerstein MK. Measurement of cell proliferation by heavy water labeling. Nature Protocols. 2007;2(12):3045–57. doi: 10.1038/nprot.2007.420 18079703 10.1038/nprot.2007.420

[46] Capper R, Britt-Compton B, Tankimanova M, Rowson J, Letsolo B, Man S, et al The nature of telomere fusion and a definition of the critical telomere length in human cells. Genes Dev. 2007;21(19):2495–508. doi: 10.1101/gad.439107 17908935 10.1101/gad.439107 PMC1993879

[47] Wherry EJ, Teichgraber V, Becker TC, Masopust D, Kaech SM, Antia R, et al Lineage relationship and protective immunity of memory CD8 T cell subsets. Nature Immunology. 2003;4(3):225–34. doi: 10.1038/ni889 12563257 10.1038/ni889

[48] Appay V, van Lier RA, Sallusto F, Roederer M. Phenotype and function of human T lymphocyte subsets: consensus and issues. Cytometry Part A: the Journal of the International Society for Analytical Cytology. 2008;73(11):975–83. 18785267 10.1002/cyto.a.20643

[49] Mahnke YD, Brodie TM, Sallusto F, Roederer M, Lugli E. The who's who of T-cell differentiation: human memory T-cell subsets. Eur J Immunol. 2013;43(11):2797–809. doi: 10.1002/eji.201343751 24258910 10.1002/eji.201343751

[50] Cieri N, Oliveira G, Greco R, Forcato M, Taccioli C, Cianciotti B, et al Generation of human memory stem T cells after haploidentical T-replete hematopoietic stem cell transplantation. Blood. 2015;125(18):2865–74. doi: 10.1182/blood-2014-11-608539 25736310 10.1182/blood-2014-11-608539

[51] Alberts B, Johnson A, Lewis J, Raff M, Roberts K, Walter P. Molecular Biology of the Cell. 4 ed New York: Garland Science; 2002.

[52] Murphy K. Janeway's Immunobiology 8 ed New York: Garland Science; 2012.

[53] von Andrian UH, Mempel TR. Homing and cellular traffic in lymph nodes. Nat Rev Immunol. 2003;3(11):867–78. doi: 10.1038/nri1222 14668803 10.1038/nri1222

[54] De Boer RJ, Noest AJ. T cell renewal rates, telomerase, and telomere length shortening. J Immunol. 1998;160(12):5832–7. 9637494

[55] Rufer N, Brummendorf TH, Kolvraa S, Bischoff C, Christensen K, Wadsworth L, et al Telomere fluorescence measurements in granulocytes and T lymphocyte subsets point to a high turnover of hematopoietic stem cells and memory T cells in early childhood. The Journal of Experimental Medicine. 1999;190(2):157–67. 10432279 10.1084/jem.190.2.157 PMC2195579

[56] Renshaw E. Stochastic population processes: analysis, approximations, simulations Oxford: Oxford University Press; 2011. xii, 652 p. p.

[57] Wilkinson DJ. Stochastic modelling for systems biology Boca Raton, FL; London: Chapman & Hall/CRC; 2006. 254 p p.

[58] Bains I, Antia R, Callard R, Yates AJ. Quantifying the development of the peripheral naive CD4(+) T-cell pool in humans. Blood. 2009;113(22):5480–7. doi: 10.1182/blood-2008-10-184184 19179300 10.1182/blood-2008-10-184184 PMC2689049

[59] Neller MA, Ladell K, McLaren JE, Matthews KK, Gostick E, Pentier JM, et al Naive CD8(+) T-cell precursors display structured TCR repertoires and composite antigen-driven selection dynamics. Immunol Cell Biol. 2015;93(7):625–33. doi: 10.1038/icb.2015.17 25801351 10.1038/icb.2015.17 PMC4533101

[60] R: A language and environment for statistical computing. Available from: http://www.R-project.org/. 2014. [cited 2016]

[61] Soetaert K. R Package FME: Inverse Modelling, Sensitivity,Monte Carlo–Applied to a Dynamic Simulation Model. Available from: https://cran.r-project.org/web/packages/FME/. [cited 2016]

[62] Stauffer HB. Contemporary Bayesian and frequentist statistical research methods for natural resource scientists Hoboken, N.J.: Wiley-Interscience; 2008. xv, 400 p. p.

