# Supplementary material for: Human TSCM cell dynamics in vivo are compatible with long-lived immunological memory and stemness
Source: PLoS Biol. 2018 Jun 22;16(6):e2005523. doi: 10.1371/journal.pbio.2005523 (PMC6033534; doi:10.1371/journal.pbio.2005523)
Supplement: S1 Fig — (PDF) [file pbio.2005523.s001.pdf]

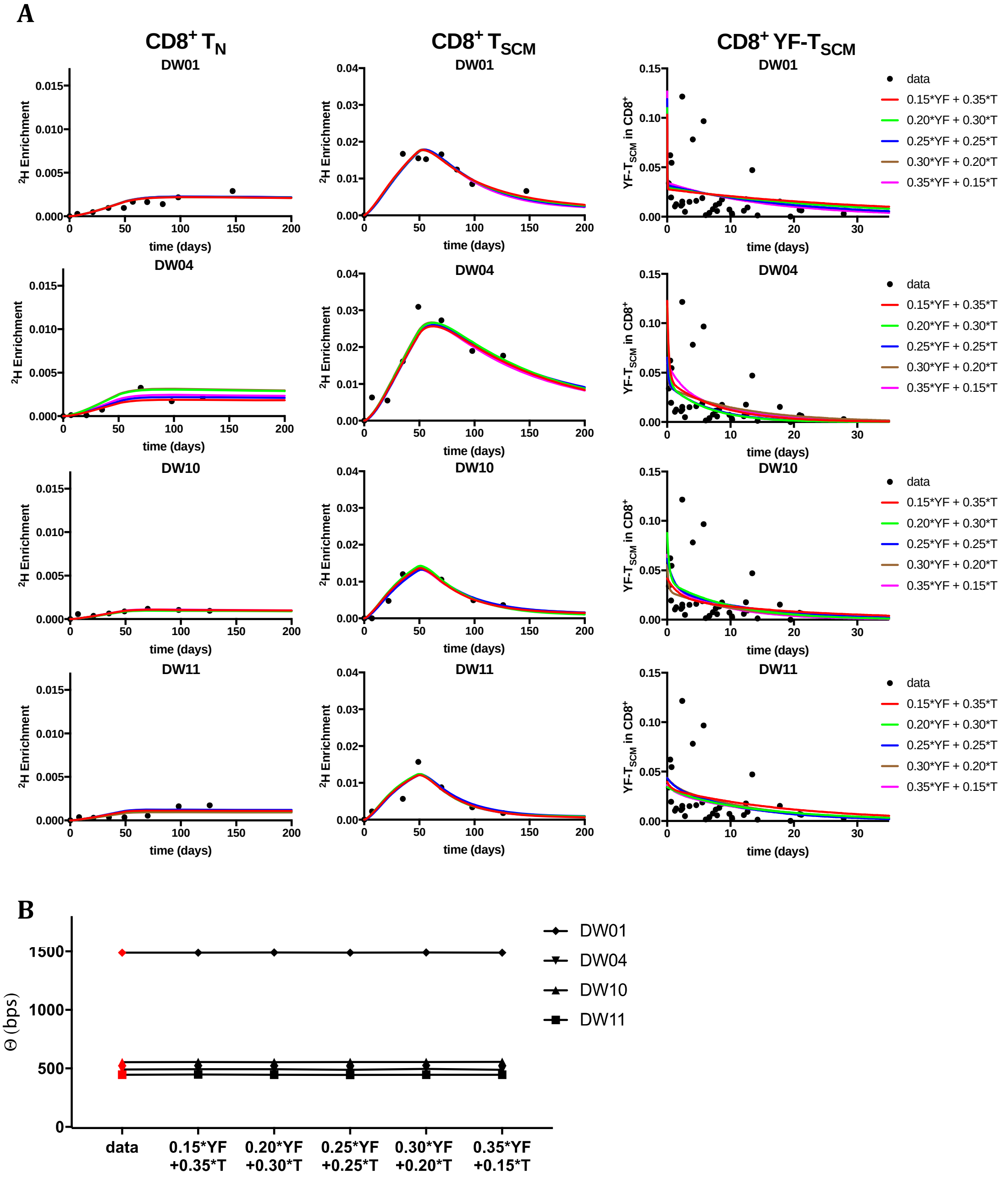

S1 Fig. Impact of different weightings when fitting CD8+ T cell data.

A) Fits to the labelling data for CD8+ naïve and T_{SCM} cells and to the YFV T_{SCM} data, when the labelling, telomere length, and YFV datasets were fitted simultaneously with the explicit heterogenity model. Each fit was generated with a different combination of weights to assess the importance of each dataset in the computation of the sum of squares during the fitting process. The weight corresponding to the labelling dataset was always fixed to 0.5. The experimental data is represented by solid black symbols. **B)** The corresponding fits to the average telomere length differences (Θ) between the T_N and T_{SCM} pools, with experimental data points in red. The number of base pairs lost in each division was taken to be δ =50bp/division. Experimental data depicted in this figure can be found in S1 Data.
