# Supplementary material for: Human TSCM cell dynamics in vivo are compatible with long-lived immunological memory and stemness
Source: PLoS Biol. 2018 Jun 22;16(6):e2005523. doi: 10.1371/journal.pbio.2005523 (PMC6033534; doi:10.1371/journal.pbio.2005523)
Supplement: S2 Fig — (PDF) [file pbio.2005523.s002.pdf]

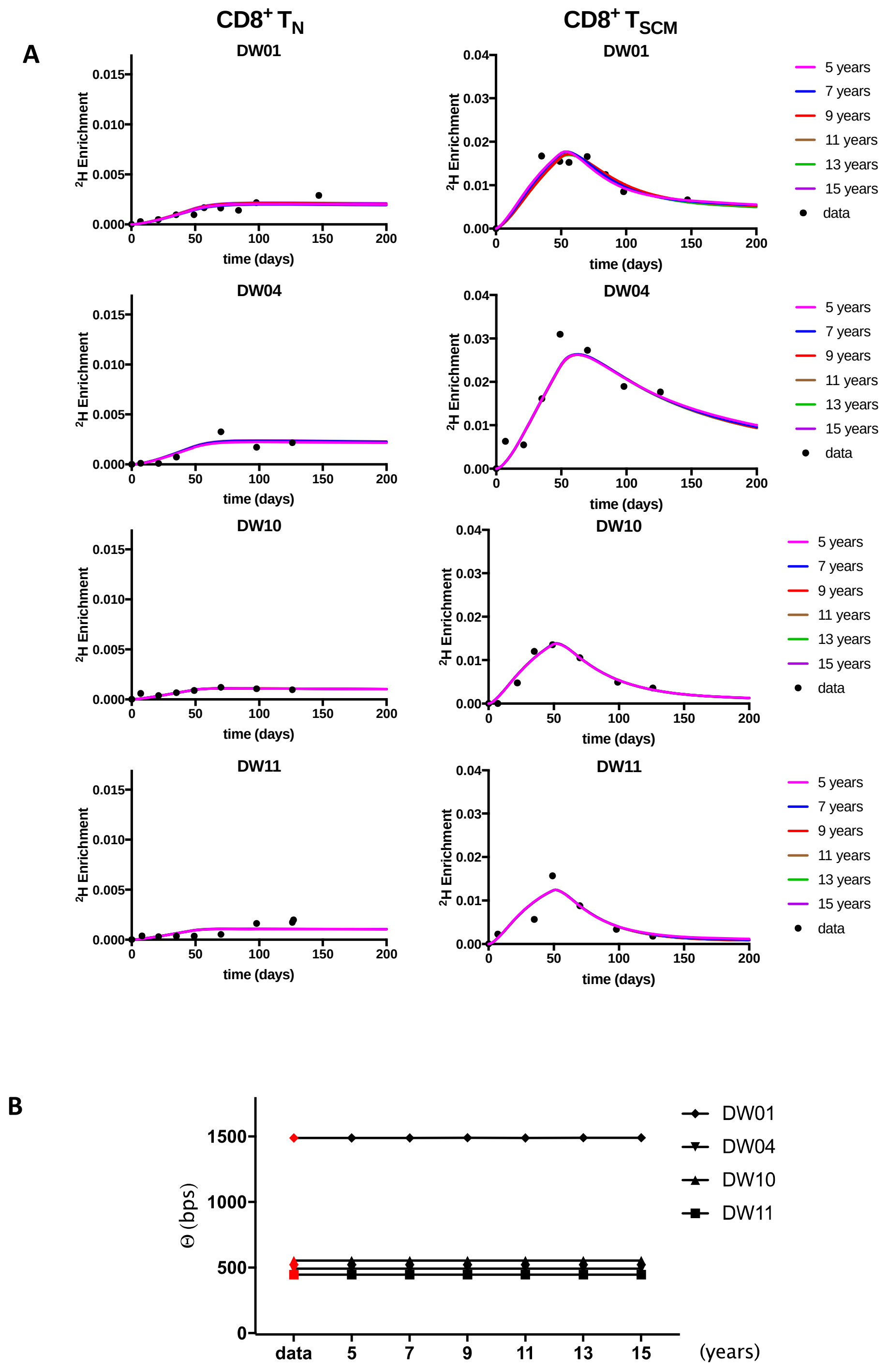

S2 Fig. CD8$^+$ T cell data are compatible with a long subpopulation half-life independent of yellow fever virus vaccine data.

A) Observed and fitted label incorporation in CD8$^+$ naïve and T_{SCM} cells when the explicit heterogeneity model was fitted to isotope labelling and telomere length datasets simultaneously, constraining the half-life of the slower subpopulation to lie between 5-6, 7-8, 9-10, 11-12, 13-14 or 14-15 years. Note that the predictions for many of the different half-lives overlie each other and so cannot be distinguished. **B)** Corresponding fits to the average telomere length differences (Θ) between the T_N and T_{SCM} pools, with experimental data points shown in red. The number of base pairs (bp) lost in each division was taken to be δ =50bp/division. Experimental data depicted in this figure can be found in S1 Data.
