# Supplementary material for: Human TSCM cell dynamics in vivo are compatible with long-lived immunological memory and stemness
Source: PLoS Biol. 2018 Jun 22;16(6):e2005523. doi: 10.1371/journal.pbio.2005523 (PMC6033534; doi:10.1371/journal.pbio.2005523)
Supplement: S4 Fig — (PDF) [file pbio.2005523.s004.pdf]

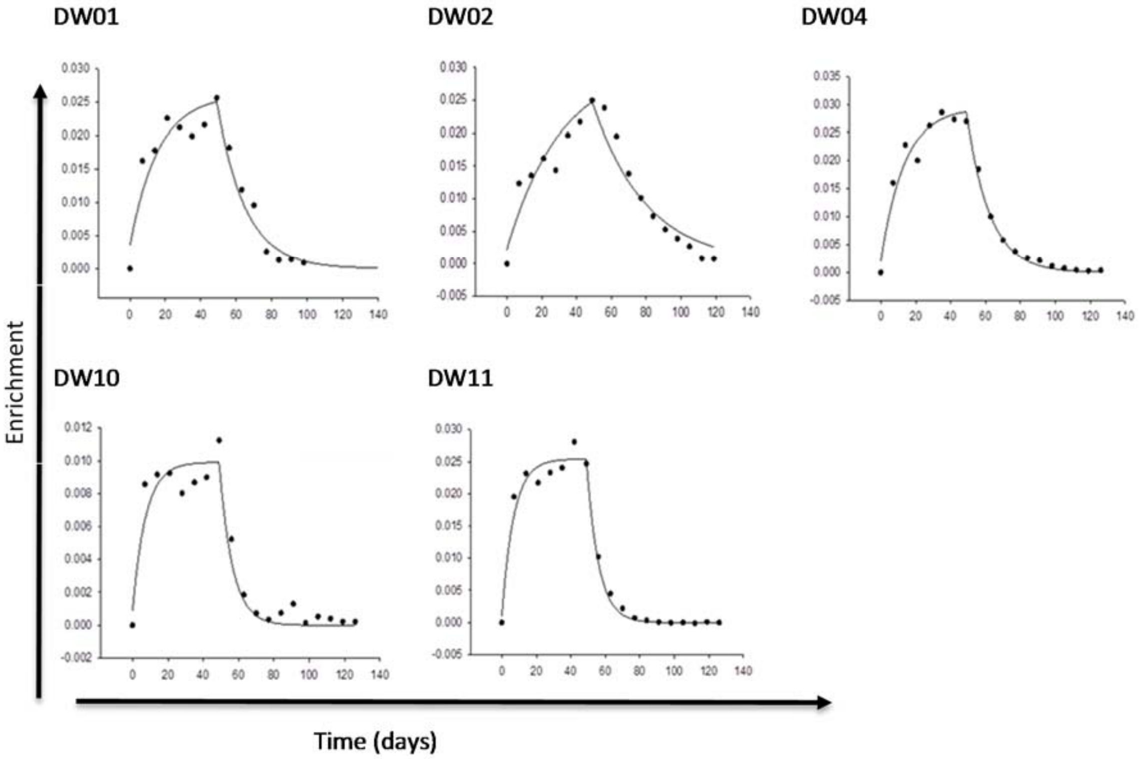


**S4 Fig. Deuterium enrichment in body water.**

Body water enrichment as measured in saliva (dots) with best fit curves (solid line) in all volunteers. Parameter estimates from the model fits are given in S4 Table.
