# Supplementary material for: Human TSCM cell dynamics in vivo are compatible with long-lived immunological memory and stemness
Source: PLoS Biol. 2018 Jun 22;16(6):e2005523. doi: 10.1371/journal.pbio.2005523 (PMC6033534; doi:10.1371/journal.pbio.2005523)
Supplement: S1 Table — (PDF) [file pbio.2005523.s005.pdf]

	T_{SCM} population (one-tailed p-values)		T_N population (one-tailed p-values)	
**id**	**$CD4^+$**	**$CD8^+$**	**$CD4^+$**	**$CD8^+$**
**DW01**	2.75e-08	2.53e-06	0.93	0.36
**DW02**	2.61e-11		0.21	
**DW04**	5.80e-07	6.50e-02	1.00	0.48
**DW10**	1.09e-01	4.86e-07	0.63	0.52
**DW11**	9.55e-04	5.65e-06	0.53	0.66
**median**	**5.80e-07**	**4.09e-06**	**0.63**	**0.50**
**pooled**	**3.50e-23**	**6.10e-15**	**0.86**	**0.68**

S1 Table. Statistical comparison of the quality of fit of the homogeneous and heterogeneous models to labelling and telomere length data for T_N and T_{SCM} cells.

The table shows one-tailed p-values obtained using Fisher's F-test comparison of the homogenous model with the (implicit) heterogeneous model. Fisher's F-test for nested models takes into account the different number of parameters in the models. The null hypothesis, that the population is kinetically homogeneous, was confidently rejected in 7/9 cases for the T_{SCM} population but in 0/9 cases for the T_N population. P values were pooled using Fisher's combined p (bottom row).
