# Supplementary material for: Human TSCM cell dynamics in vivo are compatible with long-lived immunological memory and stemness
Source: PLoS Biol. 2018 Jun 22;16(6):e2005523. doi: 10.1371/journal.pbio.2005523 (PMC6033534; doi:10.1371/journal.pbio.2005523)
Supplement: S2 Table — (PDF) [file pbio.2005523.s006.pdf]

id	P_n [day^{-1}] (95% CI)	p_{s1} [day^{-1}] 95% CI	p_{s2} [day^{-1}] (95% CI)	d_{s1}[day^{-1}] 95% CI	d_{s2} [day^{-1}] 95% CI	**Self-renewal** [d] 95% CI
**DW01**	0.0005	0.005	0.021	0.087	0.0214	7300
	(0.0003,0.0020)	(0,0.2)	(0.0015,0.0661)	(0.003,0.20)	(0.0016,0.0666)	(1800,12500)
**DW04**	0.0004	0	0.003	0.013	0.0033	2400
	(0.0003,0.0008)	(0,0.2)	(0.0001,0.0078)	(0.008,0.35)	(0.0005,0.0085)	(1200,5300)
**DW10**	0.0006	0.025	0.002	0.028	0.0022	4800
	(0.0005,0.0011)	(0,0.1055)	(0,0.0083)	(0.02,0.15)	(0.0003,0.0094)	(1400,8400)
**DW11**	0.0003	0.029	0	0.031	0.0002	4400
	(0.0001,0.0006)	(0.0032,0.183)	(0,0.0081)	(0.02,0.31)	(0.0001,0.0086)	(1700,9500)
**MEDIAN**	**0.0005**	**0.015**	**0.002**	**0.029**	**0.0027**	**4600**
	**(0.0003,0.001)**	**(0,0.19)**	**(0.0001,0.008)**	**(0.01,0.26)**	**(0.0004,0.009)**	**(1500,8900)**

S2 Table. Parameter estimates for $CD8^+$ T_{SCM} cells from the explicit heterogeneity model. Parameter estimates with 95% CI (in parentheses) obtained by fitting the explicit heterogeneity model to the labelling, telomere length and YFV datasets for $CD8^+$ T cells simultaneously. The table shows the fitted parameters p_n (the proliferation rate of the naïve population), p_{s1} and p_{s2} (the proliferation rates of the two T_{SCM} subpopulations) as well as the derived parameters d_{s1} and d_{s2} (the disappearance rates of the two T_{SCM} subpopulations) and the degree of self-renewal of the long-lived subpopulation T_{SCM2}. d_{s1} and d_{s2} were calculated using the steady state constraints $(1-f)2^k\Delta T_N+p_{s1}T_{SCM1}=d_{s1}T_{SCM1}$ and $f2^k\Delta T_N+p_{s2}T_{SCM2}=d_{s2}T_{SCM2}$ and the degree of self-renewal of the T_{SCM2} subpopulation was calculated as $1/(d_{s2}-p_{s2})$. This table is a continuation of the table of parameters in the main text (Table 1).
