# Supplementary material for: Human TSCM cell dynamics in vivo are compatible with long-lived immunological memory and stemness
Source: PLoS Biol. 2018 Jun 22;16(6):e2005523. doi: 10.1371/journal.pbio.2005523 (PMC6033534; doi:10.1371/journal.pbio.2005523)
Supplement: S3 Table — (PDF) [file pbio.2005523.s007.pdf]

id	weight combination	half-live T_{SCM1} [years]	half-live T_{SCM2} [years]	T_{SCM2}/T_{SCM}	f
**DW01**	0.15*YF+0.35*T	0.02	23.87	0.25	0.000
	0.20*YF+0.30*T	0.02	18.67	0.25	0.000
	0.25*YF+0.25*T	0.02	13.92	0.25	0.001
	0.30*YF+0.20*T	0.02	14.15	0.26	0.001
	0.35*YF+0.15*T	0.02	11.20	0.25	0.001
	**median**	**0.02**	**14.15**	**0.25**	**0.001**
**DW04**	0.15*YF+0.35*T	0.18	5.49	0.35	0.017
	0.20*YF+0.30*T	0.18	4.26	0.33	0.021
	0.25*YF+0.25*T	0.14	4.59	0.58	0.041
	0.30*YF+0.20*T	0.14	7.93	0.58	0.023
	0.35*YF+0.15*T	1.84	7.88	0.51	0.196
	**median**	**0.18**	**5.49**	**0.51**	**0.023**
**DW10**	0.15*YF+0.35*T	1.29	14.45	0.52	0.087
	0.20*YF+0.30*T	0.23	6.92	0.44	0.026
	0.25*YF+0.25*T	0.69	9.09	0.51	0.075
	0.30*YF+0.20*T	0.15	7.84	0.52	0.020
	0.35*YF+0.15*T	0.50	6.27	0.53	0.083
	**median**	**0.50**	**7.84**	**0.52**	**0.075**
**DW11**	0.15*YF+0.35*T	0.38	13.43	0.82	0.116
	0.20*YF+0.30*T	2.31	11.20	0.83	0.500
	0.25*YF+0.25*T	0.90	8.39	0.25	0.001
	0.30*YF+0.20*T	0.91	9.36	0.26	0.001
	0.35*YF+0.15*T	0.78	9.87	0.25	0.001
	**median**	**0.90**	**9.87**	**0.82**	**0.307**

S3 Table. Impact of relative weighting of telomere length and YFV data.

Parameter estimates (and medians) estimated using the explicit heterogeneity model for the five weighting strategies in S1 Fig. The table shows half-life estimates for the T_{SCM1} and T_{SCM2} populations respectively, the relative size of the T_{SCM2} subpopulation (T_{SCM2}/T_{SCM}), and the fraction of cells generated from each clonal burst that enter the long-lived T_{SCM2} subpopulation (*f*). Different rows (for a given individual) correspond to different weighting strategies, where the weights applied to the yellow fever virus (YF) and telomere length (T) datasets are indicated in the second column "weight combination", and the weight applied to the labelling dataset was in all cases 0.5.
