# Supplementary material for: Human TSCM cell dynamics in vivo are compatible with long-lived immunological memory and stemness
Source: PLoS Biol. 2018 Jun 22;16(6):e2005523. doi: 10.1371/journal.pbio.2005523 (PMC6033534; doi:10.1371/journal.pbio.2005523)
Supplement: S4 Table — (PDF) [file pbio.2005523.s008.pdf]

ID	*fr*	*δ*	β
**DW01**	0.026	0.063	0.0035
**DW02**	0.031	0.032	0.0022
**DW04**	0.029	0.075	0.0021
**DW10**	0.010	0.135	0.0009
**DW11**	0.026	0.145	0.0011

S4 Table. Estimates of body water parameters.

Parameter estimates obtained by fitting an empirical model (equations 5 & 6 main text) to the saliva enrichment in each individual. *fr* represents the fraction of deuterium in water, δ the turnover rate per day of body water, and β the baseline saliva enrichment. The corresponding fits are provided in S4 Fig.
