# Supplementary material for: Human TSCM cell dynamics in vivo are compatible with long-lived immunological memory and stemness
Source: PLoS Biol. 2018 Jun 22;16(6):e2005523. doi: 10.1371/journal.pbio.2005523 (PMC6033534; doi:10.1371/journal.pbio.2005523)
Supplement: S1 Text — (PDF) [file pbio.2005523.s009.pdf]

Supplementary Methods

The explicit kinetic heterogeneity model for telomere length data

Following de Boer et al. [1], we derived an ODE model to describe the change in the average telomere lengths in the T_N population and in each of the T_{SCM} subpopulations (T_{SCM1} and T_{SCM2}) under the explicit kinetic heterogeneity model. Each of the three populations considered (T_N, T_{SCM1} and T_{SCM2}) was modelled as a series of n compartments. The equations for the number of cells in the T_{Ni}, T_{SCM1i} and T_{SCM2i} compartments are:

$$\dot{T}_{N_i} = 2p_n T_{N_{i-1}} - (p_n + \Delta + d_n)T_{N_i} \quad \text{(S1)}$$

$$\dot{T}_{SCM1_i} = 2p_{s1} T_{SCM1_{i-1}} - (p_{s1} + d_{s1})T_{SCM1_i} + \Delta f 2^k T_{N_{i-C}} \quad \text{(S2)}$$

$$\dot{T}_{SCM2_i} = 2p_{s2} T_{SCM2_{i-1}} - (p_{s2} + d_{s2})T_{SCM2_i} + \Delta(1-f) 2^k T_{N_{i-C}} \quad \text{(S3)}$$

where p_n, f, Δ, d_n, p_s, d_s, k and C are as in Methods. The equations for the average number of divisions undergone by cells in the T_N, T_{SCM1} and the T_{SCM2} pools (following the derivation in [1]) are:

$$\dot{\mu}_{TN} = 2p_n \quad \text{(S4)}$$

$$\dot{\mu}_{TSCM1} = 2p_{s1} - \Delta f 2^k \frac{T_N}{T_{SCM1}} (\mu_{T_{SCM1}} - \mu_{T_N} - C) \quad \text{(S5)}$$

$$\dot{\mu}_{TSCM2} = 2p_{s2} - \Delta(1-f) 2^k \frac{T_N}{T_{SCM2}} (\mu_{T_{SCM2}} - \mu_{T_N} - C) \quad \text{(S6)}$$

If δ is the number of base pairs (bp) lost in each division then, by multiplying μ_{TN}, μ_{TSCM1} and μ_{TSCM2} by δ, the average telomere lengths (in units of bp) are obtained. So the difference between the average telomere lengths of the T_N and the T_{SCM} pool is described by:

$$\dot{\Theta} = \delta\left(\frac{T_{SCM1}}{T_{SCM}}\dot{\mu}_{T_{SCM1}} + \frac{T_{SCM2}}{T_{SCM}}\dot{\mu}_{T_{SCM2}} - \dot{\mu}_{T_N}\right) \quad \text{(S7)}$$

Fitting procedure

In the first part of the study, stable isotope labelling and telomere length data were fitted simultaneously. Isotope labelling residuals were normalized by the sum of the observations to guarantee equal contribution of all data points, and the combined sum of squares score was computed giving the same weight to both isotope labelling and telomere length data. The free parameters in the implicit heterogeneous model (equations 9, 10 and 25) were p_n, Δ, k, p_s, d_s^*, the ratio of T_N to T_{SCM} cells (T_N/ T_{SCM}) and C. During the fitting procedure, the ratio T_N/ T_{SCM} was assumed to lie within the range 6 to 120. These bounds were estimated given that the fractions of peripheral blood $CD4^+$ and $CD8^+$ naïve T cells are typically 35% and 40% respectively [2], the fraction of peripheral blood T_{SCM} cells is typically 2-3% [2, 3], and the ratio

of the total number of T_N to T_{SCM} cells in the lymph nodes can be double the ratio observed in peripheral blood (at least in macaques [2]).

In the second part of the study, isotope labelling, telomere length data and YFV tetramer data were fitted simultaneously. The parameters α, β, and *r* of the two exponentials YFV model in equation 28 were equated to the net disappearance rates d_{s1}-p_{s1}, d_{s2}-p_{s2}, and to the ratio T_{SCM2}/ T_{SCM} in equations 18, 19 and S7. In the first instance the sum of squares for labelling, telomere length and YFV were weighted in the ratio 0.5 : 0.25 : 0.25. This is essentially a subjective choice that was made because we wanted the labelling data to be the most informative with 50% of the weight (the telomere length data represented only a single timepoint, and the YFV data were cross-sectional and obtained from unrelated individuals). Other weighting schemes were also considered and discussed in the text. The free parameters were p_n, Δ, f, k, p_{s1}, p_{s2}, the ratio of T_N to T_{SCM} cells (T_N/ T_{SCM}), the relative size of the two T_{SCM} subpopulations (T_{SCM2}/T_{SCM}) and *C*. d_{s1} and d_{s2} were eliminated using the steady state constraints $(1-f)2^k\Delta T_N+p_{s1}T_{SCM1}=d_{s1}T_{SCM1}$ and $f2^k\Delta T_N+p_{s2}T_{SCM2}=d_{s2}T_{SCM2}$.

The 95% confidence intervals of parameter estimates were calculated by creating 1000 bootstrap datasets (i.e. sampling from the data with replacement), fitting the model of interest to each dataset, estimating the parameters and then taking the upper and lower 2.5% percentiles of the resulting 1000 parameter estimates.

Summary of different modelling approaches

MODEL	DATA FITTED	RATIONALE	RESULTS REPORTED
Implicit heterogeneous	CD4 & CD8 labelling & telomere length	Test whether the T_{SCM} population is homogeneous by comparing fits of homogeneous and heterogeneous versions of the basic model (chosen because models are nested and have similar numbers of free parameters) & estimate average population parameters	Fig 2, Fig 3, S1 Table
Explicit heterogeneous	CD8 labelling, telomere length, YFV	Estimate subpopulation parameters	Fig 4, Fig 5, Table 1, S1 Fig, S2 Table, S3 Table
	CD4 & CD8 labelling, telomere length	Test whether a slow and a rapid T_{SCM} subpopulation are compatible with the data independent of YFV data.	S2 Fig, S3 Fig

Supplementary References

1. De Boer RJ, Noest AJ. T cell renewal rates, telomerase, and telomere length shortening. J Immunol. 1998;160(12):5832-7.

2. Lugli E, Dominguez MH, Gattinoni L, Chattopadhyay PK, Bolton DL, Song K, et al. Superior T memory stem cell persistence supports long-lived T cell memory. The Journal of clinical investigation. 2013;123(2):594-9.

3. Gattinoni L, Lugli E, Ji Y, Pos Z, Paulos CM, Quigley MF, et al. A human memory T cell subset with stem cell-like properties. Nat Med. 2011;17(10):1290-7.

4. Bains I, Antia R, Callard R, Yates AJ. Quantifying the development of the peripheral naive CD4(+) T-cell pool in humans. Blood. 2009;113(22):5480-7.

5. Neller MA, Ladell K, McLaren JE, Matthews KK, Gostick E, Pentier JM, et al. Naive CD8(+) T-cell precursors display structured TCR repertoires and composite antigen-driven selection dynamics. Immunol Cell Biol. 2015;93(7):625-33.
