# Supplementary material for: Human TSCM cell dynamics in vivo are compatible with long-lived immunological memory and stemness
Source: PLoS Biol. 2018 Jun 22;16(6):e2005523. doi: 10.1371/journal.pbio.2005523 (PMC6033534; doi:10.1371/journal.pbio.2005523)
Supplement: S1 Data — (PDF) [file pbio.2005523.s010.pdf]

Supplementary Data

Costa del Amo *et al.* Human T_{SCM} cell dynamics *in vivo* are compatible with long-lived immunological memory and stemness

Contents

CD4$^+$ T cell labelling data 2

CD8$^+$ T cell labelling data 5

CD4$^+$ T cell telomere data 7

CD8$^+$ T cell telomere data 8

Sum of squared residuals as a function of k for CD4$^+$ T cell 9

Sum of squared residuals as a function of k for CD8$^+$ T cell 10

Fraction of CD4$^+$ T_{SCM} cells originating from self-renewal as a function of k 11

Fraction of CD8$^+$ T_{SCM} cells originating from self-renewal as a function of k 12

Half-life of CD4$^+$ T_{SCM} cells as a function of k 13

Half-life of CD8$^+$ T_{SCM} cells as a function of k 14

Precursor lifespan for CD4$^+$ T_{SCM} cells as a function of k 15

Precursor lifespan for CD8$^+$ T_{SCM} cells as a function of k 16

Distribution of antigen-specific precursor population half-lives for DW01 17

Distribution of antigen-specific precursor population half-lives for DW04 18

Distribution of antigen-specific precursor population half-lives for DW10 19

Distribution of antigen-specific precursor population half-lives for DW11 20

Gillespie simulation: 5 realisations for DW01 21

Gillespie simulation: 5 realisations for DW10 40

CD4$^+$ T cell labelling data

The deuterium enrichment in the DNA of sorted CD4+ T cell populations following 7 weeks label in 5 healthy adults (DW01, age 32; DW02, age 64; DW04, age 83; DW10, age 34; DW11, age 29) is shown. Two T cell populations were sorted naïve (TN): CD45RO$^-$CD27brightCCR7$^+$CD95$^-$ and stem cell-like memory T cells (TSCM): CD45RO$^-$CD27brightCCR7$^+$CD95$^+$. These data are depicted in Fig 3A and S3A Fig.

DW01 CD4 TN

Time (days)	M+1 abs
0.	0.00000
7.	0.00017
21.	0.00086
35.	0.00234
49.	0.00153
56.	0.00243
70.	0.00293
84.	0.00266
98.	0.00281
147.	0.00363
224.	0.00245

DW01 CD4 TSCM

Time (days)	M+1 abs
0.	0.00000
35.	0.01678
49.	0.02534
56.	0.02630
70.	0.02037
84.	0.01366
98.	0.01520
147.	0.00876
224.	0.00201

DW02 CD4 TN

Time (days)	M+1 abs
0.	0.00000
21.	0.00042
35.	0.00053
49.	0.00174
56.	0.00178
70.	0.00236
98.	0.00191
126.	0.00212

DW02 CD4 TSCM

Time (days)	M+1 abs
0.	0.00000
21.	0.00750
35.	0.01308
49.	0.02364
56.	0.02507
70.	0.02537
98.	0.00945

126.	0.00467

DW04 CD4 TN

0.	0.00000
7.	0.00024
21.	0.00002
35.	0.00160
49.	0.00215
70.	0.00635
98.	0.00443
126.	0.00382

DW04 CD4 TSCM

Time (days)	M+1 abs
0.	0.00000
7.	0.00077
21.	0.00727
35.	0.01849
49.	0.02886
70.	0.02918
98.	0.02185
126.	0.01050

DW10 CD4 TN

Time (days)	M+1 abs
0.	0.00000
7.	0.00024
21.	0.00060
35.	0.00097
49.	0.00137
70.	0.00209
98.	0.00148
126.	0.00164

DW10 CD4 TSCM

Time (days)	M+1 abs
0.	0.00000
7.	0.00014
21.	0.00443
35.	0.01621
49.	0.01880
70.	0.01603
98.	0.00691
126.	0.00492

DW11 CD4 TN

Time (days)	M+1 abs
0.	0.00000
7.	0.00039
21.	0.00041
35.	0.00038
49.	0.00116
70.	0.00210
98.	0.00165
126.	0.00198

DW11 CD4 TSCM

Time (days)	M+1 abs
0.	0.00000
7.	0.00311
35.	0.01135
49.	0.02160
70.	0.02003
98.	0.01618
126.	0.00943

CD8$^+$ T cell labelling data

The deuterium enrichment in the DNA of sorted CD8$^+$ T cell populations following 7 weeks label in 4 healthy adults (DW01, age 32; DW04, age 83; DW10, age 34; DW11, age 29) is shown. Two T cell populations were sorted naïve (TN): CD45RO$^-$CD27brightCCR7$^+$CD95$^-$ and stem cell-like memory T cells (TSCM): CD45RO$^-$CD27brightCCR7$^+$CD95$^+$. These data are depicted in Fig 3C, 5A S1A and S2A.

DW01 CD8 TN

Time (days)	M+1 abs
0.	0.00000
7.	0.00028
21.	0.00047
35.	0.00096
49.	0.00096
56.	0.00166
70.	0.00164
84.	0.00140
98.	0.00218
147.	0.00290

DW01 CD8 TSCM

Time (days)	M+1 abs
0.	0.00000
35.	0.01673
49.	0.01547
56.	0.01524
70.	0.01659
84.	0.01244
98.	0.00852
147.	0.00662

DW04 CD8 TN

0.	0.00000
7.	0.00012
21.	0.00010
35.	0.00075
70.	0.00326
98.	0.00172
126.	0.00217

DW04 CD8 TSCM

Time (days)	M+1 abs
0.	0.00000
7.	0.00630
21.	0.00548
35.	0.01612
49.	0.03096
70.	0.02727
98.	0.01895
126.	0.01764

DW10 CD8 TN

Time (days)	M+1 abs

0.	0.00000
7.	0.00059
21.	0.00037
35.	0.00066
49.	0.00088
70.	0.00120
98.	0.00106
126.	0.00095

DW10 CD8 TSCM

Time (days)	M+1 abs
0.	0.00000
7.	-0.00051
21.	0.00476
35.	0.01202
49.	0.01354
70.	0.01056
98.	0.00492
126.	0.00360

DW11 CD8 TN

Time (days)	M+1 abs
0.	0.00000
7.	0.00038
21.	0.00030
35.	0.00036
49.	0.00036
70.	0.00053
98.	0.00162
126.	0.00174

DW11 CD8 TSCM

Time (days)	M+1 abs
0.	0.00000
7.	0.00224
35.	0.00566
49.	0.01567
70.	0.00877
98.	0.00335
126.	0.00179

CD4$^+$ T cell telomere data

The difference in mean telomere length between TN CD4$^+$ T cells and TSCM CD4+ T cells in 5 healthy adults is shown. The first row is the experimental measurement, the second row the fit of the homogeneous model and the third row the fit of the heterogeneous model. All units are base pairs (bps). These data are depicted in Fig 3B and S3B.

	DW01	DW02	DW04	DW10	DW11
Data (bps)	891.0	1183.0	370.0	838.00	780.0
Homog (bps)	891.0	1183.0	370.0	838.00	780.0
Heterog (bps)	891.0	1183.0	370.0	838.00	780.0

CD8$^+$ T cell telomere data

The difference in mean telomere length between TN CD8$^+$ T cells and TSCM CD8$^+$ T cells in 4 healthy adults is shown. The first row is the experimental measurement, the second row the fit of the homogeneous model and the third row the fit of the heterogeneous model. All units are base pairs (bps). These data are depicted in Fig 3D, 5B, S1B, S2B.

	DW01	DW04	DW10	DW11
Data (bps)	1489.0	492.0	554.0	446.0
Homog (bps)	991.7	492.0	553.95	446.2
Heterog (bps)	1489.0	492.0	554.0	446.0

Sum of squared residuals as a function of k for CD4$^+$ T cell

Isotope labelling and telomere length data were fitted simultaneously for each of the 5 subjects, fixing the clonal expansion size *k* to values between 0 and 20 and leaving the remaining parameters free. Table shows the variation in the sum of squared residuals (ssr) with *k* in CD4$^+$. These results are depicted in Fig 4A.

	Sum of squared residuals				
k	DW01	DW02	DW04	DW10	DW11
0.	0.0000535	0.0000400	0.0000291	0.0000257	0.0000196
2.	0.0000542	0.0000398	0.0000287	0.0000257	0.0000196
4.	0.0000543	0.0000398	0.0000286	0.0000257	0.0000196
6.	0.0000544	0.0000398	0.0000286	0.0000257	0.0000196
8.	0.0000544	0.0000398	0.0000286	0.0000257	0.0000196
10.	0.0000544	0.0000398	0.0000286	0.0000257	0.0000196
12.	0.0000544	0.0000398	0.0000286	0.0000257	0.0000196
14.	0.0000544	0.0000398	0.0000286	0.0000257	0.0000196
16.	0.0000544	0.0000398	0.0000286	0.0000257	0.0000196
18.	0.0000544	0.0000398	0.0000286	0.0000257	0.0000196
20.	0.0000544	0.0000401	0.0000286	0.0000257	0.0000196

Sum of squared residuals as a function of k for CD8$^+$ T cell

Isotope labelling and telomere length data were fitted simultaneously for each of the 4 subjects, fixing the clonal expansion size *k* to values between 0 and 20 and leaving the remaining parameters free. Table shows the variation in the sum of squared residuals (ssr) with *k* in CD8$^+$ T cells. These results are depicted in Fig 4E.

	Sum of squared residuals			
k	DW01	DW04	DW10	DW11
0.	0.0000447	0.0000400	0.00000955	0.0000305
2.	0.0000448	0.0000401	0.00000957	0.0000305
4.	0.0000448	0.0000401	0.00000958	0.0000305
6.	0.0000448	0.0000401	0.00000958	0.0000305
8.	0.0000448	0.0000401	0.00000958	0.0000305
10.	0.0000448	0.0000401	0.00000958	0.0000305
12.	0.0000448	0.0000401	0.00000958	0.0000305
14.	0.0000448	0.0000401	0.00000958	0.0000305
16.	0.0000448	0.0000401	0.00000958	0.0000305
18.	0.0000677	0.0000401	0.00000958	0.0000305
20.	0.0002600	0.0000401	0.00000958	0.0000316

Fraction of CD4^{+} T_{SCM} cells originating from self-renewal as a function of k

Isotope labelling and telomere length data were fitted simultaneously for each of the 5 subjects, fixing the clonal expansion size *k* to values between 0 and 20 and leaving the remaining parameters free. Table shows the fraction of newly generated CD4^{+} T_{SCM} cells originating from self-renewing T_{SCM} proliferation computed as $(p_s T_{SCM})/(2^k \Delta T_N + p_s T_{SCM})$. These results are depicted in Fig 4B.

	Fraction of T_{SCM} originating from self-renewal				
k	DW01	DW02	DW04	DW10	DW11
0.	0.9046900	0.9239780	0.8027610	0.8985290	0.892434
2.	0.8944610	0.9237320	0.8057390	0.8990040	0.892844
4.	0.8812560	0.9215070	0.7373510	0.8933040	0.879097
6.	0.8713350	0.9146750	0.5653920	0.8829660	0.880648
8.	0.8806200	0.9135120	0.4282020	0.9951010	0.828965
10.	0.8250870	0.9047280	0.1604620	0.8956830	0.851048
12.	0.7932920	0.9146400	0.1268730	0.8747040	0.684472
14.	0.8378610	0.9048010	0.8064910	0.8992020	0.738447
16.	0.5136760	0.8071470	0.2987880	0.6481770	0.137627
18.	0.0617380	0.7685790	0.1432610	0.1505990	0.146310
20.	0.0680200	0.6555580	0.1357670	0.1437830	0.031008

Fraction of CD8$^+$ T_{SCM} cells originating from self-renewal as a function of k

Isotope labelling and telomere length data were fitted simultaneously for each of the 4 subjects, fixing the clonal expansion size *k* to values between 0 and 20 and leaving the remaining parameters free. Table shows the fraction of newly generated CD4$^+$ T_{SCM} cells originating from self-renewing T_{SCM} proliferation computed as $(p_s T_{SCM})/(2^k \Delta T_N + p_s T_{SCM})$. These results are depicted in Fig 4F.

	Fraction of T_{SCM} originating from self-renewal			
k	DW01	DW04	DW10	DW11
0.	0.9404500	0.8399800	0.85279000	0.8234300
2.	0.9366100	0.8089200	0.82705000	0.8245200
4.	0.9329700	0.7799100	0.79373000	0.8042800
6.	0.9342600	0.6788700	0.73452000	0.7884200
8.	0.9352000	0.5141800	0.66607000	0.4208200
10.	0.9328300	0.0525200	0.39897000	0.1481100
12.	0.9379000	0.0722200	0.05806000	0.0649700
14.	0.8954300	0.7544000	0.17276000	0.6807200
16.	0.8846000	0.0827200	0.51991000	0.0501600
18.	0.8597100	0.0982800	0.09566000	0.0877400
20.	0.8317500	0.0659000	0.09696000	0.0000000

Half-life of CD4$^+$ T_{SCM} cells as a function of k

Isotope labelling and telomere length data were fitted simultaneously for each of the 5 subjects, fixing the clonal expansion size *k* to values between 0 and 20 and leaving the remaining parameters free. Table shows the half-life of CD4$^+$ T_{SCM} cells, units are days. These results are depicted in Fig 4C.

	Half-life (days)				
k	DW01	DW02	DW04	DW10	DW11
0	596.57	475.84	281.21	312.07	869.78
2	582.26	503.21	339.36	341.75	952.51
4	527.89	495.79	259.6	329.64	860.9
6	490.81	457.76	158.32	302.11	877.04
8	529.38	451.91	120.9	301	612.76
10	361.28	410.6	82.46	339.58	704.21
12	305.55	458.37	79.33	282.71	332.25
14	389.93	410.6	358.05	352.55	400.99
16	130	202.71	98.81	100.69	121.62
18	67.38	168.96	80.87	41.7	122.86
20	67.84	110.17	80.17	41.37	108.24

Half-life of CD8$^+$ T_{SCM} cells as a function of k

Isotope labelling and telomere length data were fitted simultaneously for each of the 4 subjects, fixing the clonal expansion size *k* to values between 0 and 20 and leaving the remaining parameters free. Table shows the half-life of CD8$^+$ T_{SCM} cells, units are days. These results are depicted in Fig 4G.

	Half-life (days)			
k	DW01	DW04	DW10	DW11
0	1411.29	488.81	268.14	567.43
2	1387.09	464.53	257.23	665.89
4	1327.09	417.49	222.36	617.11
6	1357.53	288.81	174.58	583.59
8	1378.22	191.53	139.15	213.48
10	1329.27	98.31	77.36	145.33
12	1438.4	100.47	49.38	132.48
14	854.24	379.6	56.24	388.04
16	774.07	101.64	96.91	130.45
18	440.69	103.39	51.45	135.83
20	110.17	99.81	51.52	110.17

Precursor lifespan for CD4$^+$ T_{SCM} cells as a function of k

Isotope labelling and telomere length data were fitted simultaneously for each of the 5 subjects, fixing the clonal expansion size *k* to values between 0 and 20 and leaving the remaining parameters free. Table shows the antigen-specific precursor lifespans (time until the last cell specific for a given antigen dies or differentiates) for CD4$^+$ T_{SCM} cells, units are days. These results are depicted in Fig 4D.

	Precursor lifespan (days)				
k	DW01	DW02	DW04	DW10	DW11
0	1778.9	1666.3	1428.9	2355.1	2380.5
2	2271.6	1622.1	1649.4	1546.4	2527
4	1971.8	1776.8	1461.6	1777.7	3126.3
6	1886.6	443.7	1886.3	1331.8	2354.8
8	639.4	431.5	1191.7	1445.5	639.8
10	1655.2	416.9	812.9	321.4	1792.1
12	515.5	814.8	1754	358.2	878.1
14	427.2	299.4	457.8	261.7	585
16	584.5	616.8	506.2	396.4	660.9
18	515.7	866.3	604.9	308.5	908.7
20	673.4	876.4	781.1	401.5	1085.2

Precursor lifespan for CD8$^+$ T_{SCM} cells as a function of k

Isotope labelling and telomere length data were fitted simultaneously for each of the 4 subjects, fixing the clonal expansion size *k* to values between 0 and 20 and leaving the remaining parameters free. Table shows the antigen-specific precursor lifespans (time until the last cell specific for a given antigen dies or differentiates) for CD8$^+$ T_{SCM} cells, units are days. These results are depicted in Fig 4H.

	Precursor lifespan (days)			
k	DW01	DW04	DW10	DW11
0	2027.5	4455.7	1306.5	2343.7
2	2348.7	2610.9	1242.9	2687
4	3187.7	2825.1	1319.4	2615.2
6	362.2	1818.2	1168.5	1004.1
8	637.8	1368.8	1019.8	1158.2
10	2328.7	863.6	712.1	1474.2
12	751.7	866.3	477.4	1204.3
14	674.8	564.5	491.2	684
16	1765.5	569.4	418.4	727.7
18	1867.5	785.3	393.5	1030.9
20	733	995	506.7	1120.4

Distribution of antigen-specific precursor population half-lives for DW01

Gillespie simulations of the change in size of antigen-specific precursor populations within the long-lived CD8+ T_{SCM} pool were performed for each individual. Table shows the distribution of antigen-specific precursor population half-lives for DW01 .These results are depicted in Fig 6A.

Half-life (days) Bin Center	Frequency
3500.	0.020
4000.	0.110
4500.	0.150
5000.	0.190
5500.	0.270
6000.	0.180
6500.	0.040
7000.	0.020
7500.	0.020

Distribution of antigen-specific precursor population half-lives for DW04

Gillespie simulations of the change in size of antigen-specific precursor populations within the long-lived CD8+ T_{SCM} pool were performed for each individual. Table shows the distribution of half-lives for DW04 .These results are depicted in Fig 6A.

Half-life (days) Bin Center	Frequency
1640.	0.010
1650.	0.170
1660.	0.280
1670.	0.230
1680.	0.150
1690.	0.100
1700.	0.050
1710.	0.010

Distribution of antigen-specific precursor population half-lives for DW10

Gillespie simulations of the change in size of antigen-specific precursor populations within the long-lived CD8+ T_{SCM} pool were performed for each individual. Table shows the distribution of half-lives for DW10 .These results are depicted in Fig 6A.

Half-life (days) Bin Center	Frequency
3100.	0.010
3150.	0.030
3200.	0.050
3250.	0.140
3300.	0.320
3350.	0.310
3400.	0.100
3450.	0.040

Distribution of antigen-specific precursor population half-lives for DW11

Gillespie simulations of the change in size of antigen-specific precursor populations within the long-lived CD8+ T_{SCM} pool were performed for each individual. Table shows the distribution of half-lives for DW11 .These results are depicted in Fig 6A.

Half-life (days) Bin Center	Frequency
3050.	0.360
3055.	0.000
3060.	0.640

Gillespie simulation: 5 realisations for DW01

Gillespie simulations of the change in size of antigen-specific precursor populations within the long-lived T_{SCM} pool were performed for each individual. Five randomly chosen Gillespie simulations for DW01 are shown. These results are depicted in Fig 6B.

Time (days)	R1	R2	R3	R4	R5
0.	1.000000	1.000000	1.000000	1.000000	1.000000
10.	0.9992306	1.002588	1.004547	0.9956978	0.9939843
20.	0.9977266	1.004022	1.003288	0.9931444	0.9948937
30.	0.9947887	1.003813	1.000315	0.9867086	0.9890178
40.	1.002343	0.9980763	0.993075	0.9800978	0.9835967
50.	1.001364	1.000769	1.006785	0.9826512	0.9880735
60.	1.000070	1.003603	1.008709	0.9821615	0.9834568
70.	0.9971671	1.000525	1.003253	0.9800628	0.9814283
80.	1.007135	0.9933543	0.9987059	0.9814619	0.983212
90.	1.007485	0.992200	0.9905567	0.986079	0.9812534
100.	1.008674	0.9927946	0.9881434	0.9813221	0.9769864
110.	1.006016	0.9904512	0.9882134	0.9740117	0.9765317
120.	1.003882	0.9923749	0.9900321	0.9747113	0.9752377
130.	1.005491	0.9905211	0.9848208	0.976635	0.9741884
140.	1.006296	0.9856592	0.984681	0.9819167	0.9822677
150.	1.005071	0.9856592	0.9787002	0.9809023	0.9814633
160.	1.012801	0.9834207	0.9757623	0.9768449	0.9766367
170.	1.015004	0.9774045	0.9671235	0.9760754	0.9764268
180.	1.010632	0.976740	0.9667388	0.9715983	0.9770914
190.	1.010737	0.9789086	0.973314	0.9635535	0.9795746
200.	1.007205	0.9791535	0.9736988	0.9592862	0.9810435
210.	1.003463	0.9771248	0.9763219	0.9589365	0.9752027
220.	0.9986359	0.9822314	0.9698516	0.9570127	0.976217
230.	0.9965374	0.9852745	0.9635561	0.9533051	0.9785253
240.	0.9951735	0.989122	0.9675082	0.951836	0.974818
250.	0.9904868	0.9928296	0.9611777	0.9478836	0.9678929
260.	0.9877588	0.9928296	0.9596388	0.9465194	0.9628915
270.	0.9845061	0.9897516	0.9586245	0.9435464	0.9662491
280.	0.9876189	0.9823713	0.9535881	0.947149	0.9632063
290.	0.9919907	0.9781041	0.9558266	0.9445957	0.9600585
300.	0.9855553	0.9722629	0.9503355	0.9490728	0.9576452
310.	0.9829671	0.9690799	0.952329	0.9445957	0.953868
320.	0.9771963	0.9727176	0.9535532	0.9459248	0.9545325
330.	0.9839464	0.9760404	0.9520143	0.9479535	0.9505453
340.	0.9848558	0.9677508	0.952399	0.9470091	0.9553369
350.	0.9865696	0.9748162	0.9465582	0.9498423	0.9485867
360.	0.9902769	0.9725777	0.9409622	0.9466594	0.9458587
370.	0.9882134	0.9724028	0.9349465	0.9473589	0.9459636
380.	0.9836667	0.9714934	0.9348765	0.9512764	0.9391785
390.	0.9821977	0.9717382	0.9344568	0.9519759	0.9402627
400.	0.9860449	0.9624342	0.9277416	0.9527104	0.9352612
410.	0.9889829	0.958132	0.9228101	0.9538648	0.9414868
420.	0.9892976	0.9587616	0.9204319	0.9567329	0.9444947
430.	0.9853805	0.9530253	0.9142413	0.9573625	0.9400528
440.	0.9828972	0.9531302	0.9149058	0.948758	0.9335125
450.	0.9828272	0.9498423	0.9106388	0.9460648	0.9279165
460.	0.9812534	0.9584468	0.9097295	0.9450854	0.9273569

470.	0.9809386	0.9622244	0.9081206	0.9424971	0.9315189
480.	0.9823376	0.9598458	0.9059522	0.9377401	0.931414
490.	0.9880735	0.9563481	0.9071763	0.9384397	0.9220057
500.	0.9840864	0.9557185	0.9035039	0.9359912	0.9173191
510.	0.9807987	0.9597409	0.9093447	0.9346271	0.9210614
520.	0.9802391	0.9613849	0.9073861	0.9314792	0.9250835
530.	0.9801692	0.964253	0.9054276	0.9298002	0.9242091
540.	0.9788401	0.9599858	0.9069664	0.9306047	0.9251185
550.	0.9764968	0.9599508	0.9069315	0.9314792	0.9173191
560.	0.9746081	0.9497374	0.9063369	0.9212307	0.9128073
570.	0.9753426	0.9471141	0.9081556	0.9210559	0.9088202
580.	0.9772313	0.9467993	0.9078758	0.9170684	0.9091699
590.	0.9750628	0.9462047	0.9082255	0.9157043	0.9097995
600.	0.9751678	0.9464145	0.9068965	0.9179779	0.9027694
610.	0.9721249	0.947254	0.9121428	0.9184675	0.9056724
620.	0.9749929	0.9402585	0.913192	0.9135357	0.9011606
630.	0.9740486	0.9368307	0.9214461	0.9163339	0.9004611
640.	0.9731392	0.9314442	0.9191727	0.9179779	0.9005311
650.	0.9755874	0.9281912	0.9140314	0.9116819	0.899202
660.	0.9763919	0.9283661	0.9094846	0.9103178	0.9010557
670.	0.9703412	0.9220002	0.9066867	0.9040918	0.8997266
680.	0.9728244	0.9135707	0.9101841	0.9047214	0.8993069
690.	0.9695018	0.9110523	0.9091699	0.8992299	0.8959493
700.	0.9595339	0.9076245	0.9079807	0.8979008	0.9018251
710.	0.956561	0.9008389	0.9012655	0.8909053	0.900636
720.	0.9549872	0.9012586	0.9013355	0.8905905	0.8986773
730.	0.9572955	0.8896461	0.9024897	0.8909752	0.9007409
740.	0.9562463	0.8900309	0.9002163	0.8899959	0.893536
750.	0.9567709	0.8899959	0.9027694	0.888282	0.8966838
760.	0.9546024	0.8930039	0.8980128	0.8888416	0.8932213
770.	0.9480621	0.8938434	0.8993419	0.8841196	0.8952498
780.	0.9458587	0.8948578	0.8954597	0.8846793	0.8915425
790.	0.9413469	0.8841546	0.8954946	0.8861483	0.8876952
800.	0.9404376	0.8797125	0.8942356	0.886638	0.8833233
810.	0.9408222	0.8807268	0.8994818	0.8901358	0.8840228
820.	0.944005	0.8754802	0.9010557	0.8863582	0.8813997
830.	0.9430956	0.8806569	0.9065468	0.8891914	0.8861213
840.	0.9462084	0.8795726	0.9002862	0.8841546	0.885247
850.	0.949636	0.8776138	0.8983626	0.8846093	0.8831135
860.	0.9488316	0.8795027	0.8928715	0.8767394	0.8818194
870.	0.9522241	0.8810416	0.8962291	0.8780686	0.8865411
880.	0.9522241	0.8728219	0.8986074	0.877404	0.8875553
890.	0.9556866	0.8724722	0.8971735	0.8843295	0.8889194
900.	0.9619472	0.877439	0.8959143	0.8769842	0.8942006
910.	0.9628915	0.8708982	0.893571	0.8707233	0.8873455
920.	0.962192	0.8677852	0.8973833	0.8746058	0.882449
930.	0.9566659	0.8679251	0.8909829	0.8707932	0.8832884
940.	0.9560714	0.8739063	0.889444	0.8707233	0.8813997
950.	0.949531	0.8759699	0.8934311	0.874186	0.886576
960.	0.9467331	0.8747807	0.8896888	0.867995	0.8783919
970.	0.948132	0.8735215	0.8875203	0.8626785	0.8755239
980.	0.9472227	0.8770892	0.8827987	0.8652318	0.8680742
990.	0.9354711	0.8732067	0.8812598	0.8597404	0.8577916
1000.	0.9378494	0.8756551	0.8797559	0.861839	0.8577566
1010.	0.9347716	0.8654067	0.8760136	0.8663161	0.8556231
1020.	0.9312041	0.8639377	0.8738101	0.8711081	0.8506917

1030.	0.9249786	0.8588309	0.8684939	0.8637278	0.8571271
1040.	0.9252934	0.861839	0.8660457	0.8644623	0.8574768
1050.	0.9199771	0.8578166	0.8630728	0.8646022	0.8576517
1060.	0.9261327	0.858796	0.8553783	0.8601601	0.8589108
1070.	0.9249436	0.8591458	0.853070	0.8588659	0.8566723
1080.	0.9210614	0.8612444	0.847334	0.8565574	0.8607994
1090.	0.9185781	0.8640776	0.8469493	0.8551933	0.8600649
1100.	0.9083654	0.8632731	0.8407587	0.854214	0.8532099
1110.	0.8995517	0.8567673	0.8391499	0.849422	0.8541542
1120.	0.9049029	0.8542839	0.8324347	0.8517655	0.8566723
1130.	0.9080157	0.8573969	0.8310357	0.8488623	0.8574069
1140.	0.9043433	0.8549135	0.8328894	0.8504713	0.8540143
1150.	0.9035039	0.8559628	0.8289722	0.8517655	0.851636
1160.	0.9044133	0.8579565	0.8283426	0.8505413	0.8476139
1170.	0.8997266	0.8546336	0.8303012	0.8520453	0.8409686
1180.	0.9003912	0.8488973	0.8257545	0.8505763	0.8357573
1190.	0.8925567	0.8469736	0.8245303	0.8502265	0.8314554
1200.	0.8901784	0.838649	0.8239707	0.8555431	0.8283076
1210.	0.8889543	0.8366902	0.8187594	0.852500	0.8261042
1220.	0.8922769	0.8326678	0.8239008	0.8543538	0.8302662
1230.	0.8901435	0.8288903	0.8202984	0.8505063	0.822257
1240.	0.882379	0.8318633	0.818025	0.8521852	0.8219072
1250.	0.8889893	0.8353261	0.8188294	0.8570122	0.8234111
1260.	0.8890592	0.832458	0.8148073	0.8555781	0.8240407
1270.	0.8877652	0.8275961	0.8138979	0.8523251	0.8232363
1280.	0.8895489	0.8280858	0.8121142	0.8495969	0.8228166
1290.	0.8840578	0.8275611	0.8141778	0.8487574	0.8237959
1300.	0.8765731	0.8351512	0.8106103	0.8424965	0.8247402
1310.	0.874090	0.8295548	0.8040699	0.8477781	0.8233062
1320.	0.8695782	0.8213351	0.798439	0.8458893	0.8212077
1330.	0.8701028	0.820006	0.791409	0.8403628	0.8173605
1340.	0.8716067	0.8217199	0.7894853	0.8386839	0.8162763
1350.	0.8667802	0.8185019	0.7937523	0.8344517	0.8127437
1360.	0.8673397	0.8204257	0.7895553	0.8372848	0.8115546
1370.	0.8628979	0.8220347	0.7902898	0.8395584	0.8139329
1380.	0.8653112	0.8257772	0.7858829	0.8321431	0.8139329
1390.	0.868354	0.8271064	0.7826653	0.8278759	0.8126738
1400.	0.8668151	0.8243082	0.7868273	0.8280858	0.8133383
1410.	0.8627231	0.821440	0.7828401	0.824553	0.8101556
1420.	0.8631777	0.8241683	0.7813362	0.8206705	0.8091413
1430.	0.8659407	0.8176975	0.7824554	0.8257422	0.8119743
1440.	0.8597851	0.8125208	0.7820706	0.8238535	0.8062734
1450.	0.8624433	0.8115065	0.7817909	0.8190266	0.8058537
1460.	0.8628629	0.8094777	0.7781535	0.8160185	0.8076374
1470.	0.861499	0.812171	0.7765446	0.8148293	0.8099108
1480.	0.8658358	0.8069244	0.7776638	0.8087432	0.8140728
1490.	0.868424	0.8040562	0.7764397	0.8083585	0.8100157
1500.	0.8694732	0.8074141	0.7783284	0.809163	0.8116245
1510.	0.868354	0.8040212	0.7750057	0.8067145	0.8102605
1520.	0.8693333	0.8042661	0.7768943	0.801398	0.8168708
1530.	0.8705574	0.8025172	0.7827702	0.7995442	0.8176053
1540.	0.8643319	0.8017128	0.7776638	0.7980401	0.8150171
1550.	0.8651713	0.8037764	0.7778037	0.7990894	0.8116595
1560.	0.8625132	0.8079038	0.7714382	0.7996491	0.808197
1570.	0.8617088	0.8083934	0.7686403	0.8034266	0.8083719
1580.	0.8619536	0.8113666	0.7623797	0.8008383	0.805434

1590.	0.8660806	0.8145495	0.7595817	0.7992643	0.7960957
1600.	0.8597502	0.8159486	0.7546152	0.7995791	0.7916888
1610.	0.8593305	0.8209853	0.7567837	0.7924437	0.788646
1620.	0.857302	0.8194813	0.756364	0.7906598	0.7820706
1630.	0.850167	0.8224544	0.7524118	0.7931083	0.7852534
1640.	0.8453405	0.8195862	0.7473055	0.7909397	0.7867224
1650.	0.850237	0.8193064	0.7483197	0.7893657	0.7845889
1660.	0.8449907	0.8210203	0.746606	0.7933881	0.784414
1670.	0.8478587	0.8241683	0.7485996	0.7981451	0.7799721
1680.	0.8484882	0.8236436	0.7511528	0.7959065	0.7802869
1690.	0.843137	0.8178374	0.7502434	0.7943675	0.7807066
1700.	0.8419129	0.8164033	0.7431434	0.7991594	0.7808465
1710.	0.8406888	0.8129755	0.7405204	0.7974805	0.7879465
1720.	0.8464597	0.8088132	0.7381071	0.8002787	0.7889608
1730.	0.845935	0.8063648	0.7398558	0.8016078	0.7909893
1740.	0.8452355	0.8054903	0.7400656	0.7941926	0.7922834
1750.	0.8463897	0.8030769	0.7399957	0.7946473	0.7957459
1760.	0.8493976	0.8037415	0.7400656	0.7936679	0.7937173
1770.	0.8424725	0.8021325	0.7356238	0.788876	0.7937873
1780.	0.8405489	0.8052105	0.7343298	0.7896456	0.7947316
1790.	0.8314554	0.8069594	0.7239072	0.7876168	0.7914789
1800.	0.8239357	0.8047208	0.7294332	0.787407	0.784449
1810.	0.8239707	0.8022724	0.7309722	0.7853433	0.7846588
1820.	0.8209629	0.809023	0.7243619	0.7802716	0.7870721
1830.	0.8162413	0.8108419	0.7233826	0.7801316	0.785813
1840.	0.8151571	0.8131154	0.7134147	0.7754097	0.7817909
1850.	0.8171506	0.8086383	0.7121556	0.7696734	0.7867923
1860.	0.8177102	0.8103172	0.7110714	0.7700232	0.7894853
1870.	0.8163112	0.8038813	0.7124004	0.7638322	0.7818608
1880.	0.8147023	0.8034966	0.7068394	0.759425	0.7845889
1890.	0.8148422	0.8006284	0.7039015	0.757886	0.7832248
1900.	0.8124639	0.7968858	0.7035168	0.7600896	0.778818
1910.	0.812394	0.7968159	0.7019778	0.7546681	0.7813712
1920.	0.8150171	0.7907648	0.7021177	0.7543882	0.780252
1930.	0.8058537	0.7899953	0.706035	0.7512752	0.781616
1940.	0.8035453	0.7875119	0.6987602	0.7492465	0.7794476
1950.	0.8059236	0.7845738	0.694738	0.7474977	0.7796924
1960.	0.8112748	0.7851684	0.6958572	0.7447695	0.7757052
1970.	0.8074275	0.785763	0.6994247	0.7442448	0.774551
1980.	0.8094911	0.7870222	0.6978858	0.7482672	0.7714033
1990.	0.8065182	0.7883863	0.696137	0.7456089	0.7751806
2000.	0.8092462	0.7876518	0.6951228	0.7412717	0.7730121
2010.	0.8117295	0.7875819	0.6943883	0.7373192	0.7697245
2020.	0.8113098	0.7902052	0.6915553	0.7316529	0.7736417
2030.	0.8090714	0.7906598	0.6946681	0.728295	0.7742013
2040.	0.8071128	0.7887361	0.6945981	0.7316878	0.7747609
2050.	0.7995932	0.7865675	0.6964868	0.7302887	0.7798672
2060.	0.797040	0.7850984	0.6979207	0.7311982	0.7807066
2070.	0.7985089	0.7853783	0.7014183	0.7282251	0.7822455
2080.	0.7996281	0.7867424	0.6987602	0.7292744	0.7839243
2090.	0.7960257	0.7884912	0.6959272	0.7258117	0.7798672
2100.	0.7918287	0.7906948	0.6956474	0.7239578	0.7730821
2110.	0.7864775	0.7959765	0.6896317	0.7195507	0.7681856
2120.	0.778713	0.7945074	0.6921149	0.7247273	0.7669964
2130.	0.7781185	0.7921639	0.6922199	0.7258466	0.767661
2140.	0.7730121	0.793528	0.6882327	0.7257417	0.7679408

2150.	0.771788	0.7940177	0.6858894	0.7225937	0.7636039
2160.	0.7710885	0.7976553	0.684980	0.7185014	0.7679408
2170.	0.7704939	0.7956966	0.6888623	0.7155982	0.7714382
2180.	0.7715082	0.7977953	0.6881278	0.7137444	0.7705988
2190.	0.7719279	0.8006984	0.6802234	0.7137095	0.7644083
2200.	0.7677309	0.7981101	0.6840357	0.7119256	0.7658423
2210.	0.7663669	0.7961513	0.6852598	0.7134996	0.7643033
2220.	0.7630442	0.7909747	0.6851898	0.7139543	0.7625896
2230.	0.7582877	0.7942276	0.6882676	0.7131498	0.7600014
2240.	0.7556645	0.7921639	0.6861691	0.7089176	0.757763
2250.	0.7541606	0.8016428	0.6830214	0.711331	0.7601063
2260.	0.7526217	0.8040562	0.6831963	0.708218	0.7581128
2270.	0.7501734	0.8110517	0.6864489	0.7067839	0.7579029
2280.	0.748040	0.8050006	0.6852598	0.7059794	0.7624497
2290.	0.7474454	0.798180	0.6864839	0.6974099	0.7597566
2300.	0.7497538	0.8004535	0.6881278	0.6974099	0.7573084
2310.	0.749404	0.7914643	0.6783698	0.6978996	0.7555596
2320.	0.749404	0.7875469	0.6800835	0.6962207	0.754965
2330.	0.7467109	0.7864975	0.6717594	0.6966054	0.7556995
2340.	0.7421292	0.7861478	0.6704304	0.6945418	0.7597566
2350.	0.7432484	0.7824402	0.6706053	0.6925831	0.7607009
2360.	0.7447174	0.7819855	0.6666181	0.6886656	0.760561
2370.	0.7411849	0.7755146	0.6636802	0.6930028	0.7568537
2380.	0.7443326	0.7745003	0.6677373	0.6934925	0.7609457
2390.	0.7345396	0.7755496	0.6691363	0.692793	0.7610507
2400.	0.7305175	0.7767038	0.6720742	0.6897849	0.7660871
2410.	0.7335253	0.7757595	0.6702555	0.6881759	0.7649329
2420.	0.7307273	0.7741155	0.6704654	0.6933526	0.7627645
2430.	0.7285589	0.7653362	0.6684718	0.691114	0.7582177
2440.	0.7284539	0.7673299	0.6686816	0.6935624	0.7551049
2450.	0.7189757	0.7711424	0.6658837	0.6907642	0.7495439
2460.	0.7141142	0.7679595	0.6635054	0.6916386	0.7497887
2470.	0.7094625	0.7748151	0.6600778	0.6899948	0.7503133
2480.	0.7065596	0.7743254	0.6610221	0.6918835	0.7520971
2490.	0.7041463	0.7732411	0.6582941	0.6939822	0.7485296
2500.	0.7034818	0.7722967	0.6580143	0.692653	0.7444375
2510.	0.7043911	0.7650214	0.6559158	0.6956261	0.7404154
2520.	0.7067695	0.762538	0.6599728	0.6930028	0.7338051
2530.	0.7010335	0.7621882	0.6568251	0.6937023	0.7350992
2540.	0.7069793	0.7615936	0.6532577	0.6875463	0.7284539
2550.	0.7070842	0.7601595	0.654272	0.6936674	0.7248865
2560.	0.7119108	0.7606842	0.6494454	0.6903095	0.7250264
2570.	0.7008237	0.7618384	0.6463676	0.6905894	0.7245368
2580.	0.7006488	0.7571515	0.6462277	0.6939472	0.7282441
2590.	0.694808	0.7565918	0.647242	0.6925481	0.7332805
2600.	0.6961021	0.7529891	0.6440243	0.691184	0.7307273
2610.	0.6931992	0.7542483	0.645773	0.6889804	0.7271599
2620.	0.6926745	0.7554376	0.6421006	0.6860073	0.726985
2630.	0.6973262	0.7570115	0.6427302	0.688036	0.7279993
2640.	0.6921149	0.7584456	0.6388829	0.6883508	0.7276845
2650.	0.6954376	0.7603344	0.6409115	0.6948216	0.7294332
2660.	0.6927095	0.7633425	0.6434297	0.6949965	0.7328258
2670.	0.6940385	0.765616	0.6440592	0.6937723	0.7299929
2680.	0.6939686	0.7638322	0.6426253	0.691044	0.7271599
2690.	0.6916952	0.7628178	0.6404918	0.6874064	0.718556
2700.	0.6959622	0.7690088	0.6375539	0.6860772	0.7215989

2710.	0.6929543	0.7676097	0.6354904	0.6860772	0.719990
2720.	0.6931642	0.7710375	0.6366795	0.6848181	0.7207595
2730.	0.6893169	0.7731012	0.6366795	0.6825445	0.7211442
2740.	0.6853647	0.7747451	0.6366445	0.6831391	0.7223683
2750.	0.6802234	0.7769137	0.6381834	0.6822298	0.7194304
2760.	0.6817973	0.7818806	0.6378337	0.6803409	0.717227
2770.	0.6853647	0.7773684	0.637449	0.6774378	0.7209343
2780.	0.6872184	0.7787325	0.6360849	0.6741849	0.7165624
2790.	0.6874982	0.7766339	0.6338465	0.6780325	0.7153733
2800.	0.6782648	0.7697084	0.6314332	0.6795365	0.712960
2810.	0.6786845	0.7693236	0.6284953	0.6839786	0.7110714
2820.	0.6810978	0.7678546	0.6273412	0.6904144	0.7070842
2830.	0.6796638	0.7689039	0.6283554	0.6906593	0.7025375
2840.	0.6791042	0.7651613	0.6325524	0.6902745	0.6990749
2850.	0.6829515	0.7602295	0.6307337	0.6875463	0.6940735
2860.	0.6829165	0.7551927	0.6278308	0.6839086	0.6914853
2870.	0.6818323	0.7552277	0.6312584	0.6860422	0.6913455
2880.	0.686309	0.7550878	0.6308737	0.6881409	0.6888623
2890.	0.6842105	0.7503659	0.624823	0.6860073	0.6875682
2900.	0.6789293	0.7484421	0.6246481	0.6869167	0.6890022
2910.	0.6788244	0.7464134	0.6254175	0.6866019	0.6818323
2920.	0.679419	0.7399775	0.6232491	0.6872665	0.6851898
2930.	0.6856096	0.7367246	0.6189821	0.6848181	0.6842105
2940.	0.6859943	0.7370394	0.6231791	0.6810755	0.6807131
2950.	0.687848	0.7357802	0.6211856	0.675584	0.6769707
2960.	0.6908208	0.7308834	0.6186324	0.6739051	0.6746274
2970.	0.6936538	0.7328421	0.6131064	0.6712118	0.6756766
2980.	0.696172	0.7305686	0.6152748	0.6715966	0.6705353
2990.	0.6966267	0.7263363	0.6172334	0.6687284	0.6749072
3000.	0.6982006	0.7318977	0.6162541	0.6661401	0.6809928
3010.	0.6943184	0.7316878	0.6133862	0.661593	0.6780899
3020.	0.6937937	0.7296242	0.6090143	0.6597742	0.6795239
3030.	0.6900514	0.733017	0.6111128	0.6573258	0.6839308
3040.	0.6855046	0.7352555	0.6114975	0.655367	0.6775303
3050.	0.6810278	0.7365147	0.608070	0.6535832	0.6775653
3060.	0.6827766	0.7355004	0.606636	0.6458881	0.6739279
3070.	0.6825318	0.7320726	0.6126866	0.6424953	0.6726688
3080.	0.679349	0.7278404	0.6158693	0.6420406	0.6755018
3090.	0.6744875	0.7270009	0.6139807	0.6466576	0.6814825
3100.	0.6758515	0.7264763	0.6082798	0.6450487	0.6845253
3110.	0.6763412	0.7254968	0.6098537	0.6433698	0.6843854
3120.	0.6718644	0.722139	0.610798	0.6408514	0.6828815
3130.	0.6730535	0.7214745	0.6055517	0.6404666	0.6860992
3140.	0.6734383	0.7170323	0.599676	0.6408164	0.6859593
3150.	0.6702206	0.7086727	0.5988365	0.6378433	0.6866938
3160.	0.6665831	0.7071337	0.5977523	0.6365842	0.6826367
3170.	0.6655689	0.7008027	0.5947794	0.632072	0.6847702
3180.	0.6702905	0.7013974	0.5969828	0.6329465	0.6841406
3190.	0.6665482	0.7075185	0.5948494	0.629064	0.6833362
3200.	0.6622812	0.7045104	0.5920864	0.6318622	0.685050
3210.	0.665394	0.6975499	0.5922962	0.6313375	0.6842455
3220.	0.6666881	0.6982844	0.5928208	0.6252514	0.6760264
3230.	0.6663383	0.6987041	0.5870849	0.6219985	0.6753969
3240.	0.664030	0.6961858	0.5860357	0.6208443	0.6766559
3250.	0.6665132	0.692758	0.5840071	0.6240273	0.6789293
3260.	0.6612669	0.684853	0.585616	0.6220335	0.675187

3270.	0.6651142	0.6851329	0.5811741	0.6129394	0.6775303
3280.	0.6580492	0.6831391	0.5811741	0.6099663	0.6810278
3290.	0.6595882	0.6805158	0.5816638	0.6019215	0.6800835
3300.	0.6620364	0.6774728	0.5823983	0.598039	0.6747673
3310.	0.6593084	0.678697	0.5796003	0.5939466	0.673718
3320.	0.6615817	0.675549	0.5756131	0.5928973	0.675152
3330.	0.6606374	0.6764584	0.5805446	0.5894695	0.673718
3340.	0.6638901	0.6799212	0.5783062	0.5904489	0.6743127
3350.	0.661092	0.6764235	0.5808944	0.5895395	0.6767259
3360.	0.6607773	0.6761436	0.5811741	0.5915681	0.6833012
3370.	0.6598679	0.6664899	0.5811741	0.5912883	0.6774954
3380.	0.661232	0.6671194	0.5766624	0.5892946	0.6770757
3390.	0.6662683	0.6672943	0.5784811	0.5853772	0.6726688
3400.	0.6647645	0.6665598	0.5814889	0.5872309	0.6732984
3410.	0.6675275	0.6665948	0.5752284	0.5881404	0.6727038
3420.	0.6632605	0.6660702	0.5706816	0.5864614	0.6724939
3430.	0.664030	0.6661401	0.5783062	0.5850273	0.6693112
3440.	0.659763	0.6587598	0.5743191	0.5845727	0.6700107
3450.	0.6595882	0.6644962	0.5801599	0.5818444	0.6722841
3460.	0.6606724	0.6624675	0.5814889	0.5850273	0.6742777
3470.	0.6543769	0.6591446	0.5810692	0.5884202	0.6720742
3480.	0.6539222	0.6604738	0.5792505	0.5903789	0.6654639
3490.	0.6559158	0.6587598	0.5773969	0.5881753	0.6675624
3500.	0.6581542	0.6573258	0.5796703	0.5896444	0.6650792
3510.	0.657035	0.6563464	0.5774668	0.5890148	0.6664782
3520.	0.6578394	0.6528137	0.5774319	0.585517	0.6599379
3530.	0.6518237	0.6535133	0.5797752	0.5836982	0.6616517
3540.	0.6538522	0.6517993	0.5851263	0.5873009	0.6606724
3550.	0.6501449	0.6527438	0.5877844	0.5863215	0.6537473
3560.	0.6466824	0.6486514	0.5886588	0.5857619	0.6511942
3570.	0.6439893	0.6451536	0.588414	0.5833834	0.6521735
3580.	0.6390578	0.6446989	0.5896381	0.582509	0.6539572
3590.	0.6381834	0.6431949	0.5898829	0.5794309	0.6530129
3600.	0.6394076	0.6423205	0.5886938	0.5805852	0.6544818
3610.	0.6356652	0.6416909	0.5857559	0.577787	0.6523833
3620.	0.6347209	0.6410263	0.5871199	0.5693924	0.6532927
3630.	0.6325524	0.6443841	0.5839722	0.5657548	0.6516838
3640.	0.6292998	0.6428801	0.5876095	0.5604382	0.6526631
3650.	0.6255575	0.6427052	0.5880643	0.5627817	0.6503198
3660.	0.6247181	0.6364442	0.5843219	0.5619772	0.6492705
3670.	0.6216053	0.6392074	0.5829229	0.5610328	0.6480814
3680.	0.6228644	0.6378433	0.5836574	0.5628167	0.6439543
3690.	0.623424	0.6435097	0.5817338	0.5629916	0.6384283
3700.	0.6214304	0.6457483	0.5849165	0.5569754	0.6417859
3710.	0.6194368	0.6481966	0.5809293	0.5537925	0.6369943
3720.	0.623319	0.6452585	0.5776417	0.5523234	0.637449
3730.	0.6215703	0.6455034	0.577222	0.5519736	0.6297545
3740.	0.6212205	0.6412011	0.5792155	0.5504346	0.630384
3750.	0.6229693	0.6405016	0.5797052	0.5490006	0.6323076
3760.	0.6202063	0.6351151	0.5753333	0.5441387	0.631748
3770.	0.6202412	0.6279447	0.5769072	0.5422149	0.6322727
3780.	0.6181078	0.6266505	0.5789357	0.5401163	0.6302091
3790.	0.617793	0.6323869	0.5788658	0.5337853	0.6289151
3800.	0.6210107	0.629099	0.5818387	0.5320365	0.6316781
3810.	0.6193319	0.6318622	0.5759279	0.5348697	0.6335667
3820.	0.6159743	0.6292039	0.5758229	0.5325612	0.6336017

3830.	0.610763	0.6270003	0.5787259	0.5377378	0.6296145
3840.	0.6075453	0.6265106	0.5798101	0.5415154	0.6239836
3850.	0.6104133	0.6279097	0.5821534	0.5412356	0.6282156
3860.	0.6079301	0.6252864	0.5815239	0.5427746	0.6270614
3870.	0.6118472	0.6194801	0.5784111	0.5376329	0.6302441
3880.	0.6053768	0.6194102	0.5709615	0.5344149	0.6302441
3890.	0.6045724	0.6125896	0.5652255	0.5287836	0.6277259
3900.	0.6036631	0.6054542	0.5675339	0.5290984	0.6244382
3910.	0.6054118	0.6036704	0.5636167	0.5243765	0.6266767
3920.	0.6082098	0.5950309	0.559000	0.5278742	0.6232491
3930.	0.606531	0.5934919	0.5588251	0.5244114	0.6235289
3940.	0.6039429	0.5954157	0.5592098	0.5185702	0.6221649
3950.	0.6092241	0.5967098	0.5655753	0.5186052	0.6192969
3960.	0.6055517	0.5946112	0.5669393	0.5232571	0.6153448
3970.	0.6069857	0.5955555	0.5699472	0.5257756	0.6099936
3980.	0.6078251	0.5935618	0.567359	0.5251809	0.6129664
3990.	0.6078951	0.5926524	0.5657852	0.5291684	0.6153098
4000.	0.6099586	0.5941914	0.567394	0.5268248	0.6152398
4010.	0.606566	0.6008022	0.5636867	0.525111	0.6200314
4020.	0.6043976	0.5986336	0.5656103	0.5269997	0.6161492
4030.	0.6039079	0.6001376	0.5619029	0.529728	0.6103083
4040.	0.5984169	0.5988435	0.5623576	0.5275943	0.610728
4050.	0.5983119	0.5987735	0.5664147	0.5259504	0.6096088
4060.	0.6033483	0.6019564	0.5643162	0.5261253	0.6073354
4070.	0.6041527	0.6076928	0.5638266	0.5252509	0.605132
4080.	0.6039779	0.6105259	0.564561	0.5205989	0.5962834
4090.	0.6062862	0.6141986	0.5617281	0.5175908	0.5977873
4100.	0.6101335	0.6138138	0.5642462	0.5158419	0.5955139
4110.	0.6113576	0.6096165	0.5605039	0.5138832	0.5950942
4120.	0.6088394	0.6094416	0.5642462	0.5129738	0.5951642
4130.	0.6086995	0.6111555	0.5636517	0.5153522	0.592576
4140.	0.612197	0.6115403	0.566030	0.5151774	0.5938701
4150.	0.6105881	0.6096865	0.5662748	0.5165065	0.5921563
4160.	0.6090493	0.6059788	0.5668344	0.5132886	0.5908273
4170.	0.6070557	0.6086372	0.5679536	0.5149325	0.5879943
4180.	0.608035	0.608882	0.5690029	0.5164365	0.5908273
4190.	0.6041178	0.6164371	0.5708215	0.5134634	0.5927159
4200.	0.6007602	0.6176963	0.573025	0.5112249	0.5874696
4210.	0.6045724	0.6189555	0.5694925	0.506328	0.591247
4220.	0.6022641	0.6189905	0.5743541	0.5012214	0.5862805
4230.	0.598172	0.6211941	0.5761378	0.4982832	0.5869451
4240.	0.5962134	0.616542	0.5738294	0.4940859	0.5864554
4250.	0.595374	0.6168219	0.5742841	0.4990527	0.5953041
4260.	0.5923312	0.618221	0.5736545	0.4990527	0.5993962
4270.	0.5978572	0.6189905	0.5674989	0.4991577	0.5952341
4280.	0.5976474	0.6207044	0.5657152	0.501711	0.5938701
4290.	0.595409	0.6245519	0.5684782	0.5036348	0.5895681
4300.	0.5913869	0.6239923	0.5697723	0.5111899	0.5919465
4310.	0.5903726	0.6203546	0.5664147	0.508042	0.5941149
4320.	0.5848815	0.6120999	0.5679886	0.5046142	0.591282
4330.	0.5824682	0.6107358	0.5661699	0.5032151	0.5906873
4340.	0.5872248	0.6123797	0.5647709	0.5012563	0.589708
4350.	0.5871199	0.615003	0.5629871	0.5018859	0.5913169
4360.	0.5833426	0.6102111	0.5642812	0.5005568	0.5878894
4370.	0.5860357	0.6108057	0.5640014	0.4999622	0.5874696
4380.	0.5851263	0.6109107	0.5610635	0.5006967	0.5801249

4390.	0.588379	0.6140587	0.5581256	0.501711	0.5849165
4400.	0.5822933	0.6078677	0.5550478	0.5015361	0.5905824
4410.	0.5827131	0.6031457	0.5516202	0.5004519	0.5911071
4420.	0.5791806	0.6032506	0.5513754	0.500172	0.5921913
4430.	0.5789008	0.6029009	0.550571	0.4991927	0.5952341
4440.	0.5680586	0.6052094	0.5495567	0.5024806	0.595444
4450.	0.5700521	0.602551	0.5486124	0.5015711	0.5964233
4460.	0.5667645	0.6003475	0.547808	0.5035998	0.6005503
4470.	0.5663447	0.5992982	0.5434711	0.5015011	0.5990813
4480.	0.567324	0.5981789	0.5387145	0.5003119	0.6083847
4490.	0.5683383	0.5992982	0.5369307	0.5020958	0.6085946
4500.	0.5707866	0.5943314	0.5370357	0.5012913	0.6085246
4510.	0.5699822	0.5941215	0.5388194	0.4982832	0.6074404
4520.	0.5742141	0.5916381	0.5383997	0.503320	0.6047823
4530.	0.5664147	0.588700	0.5393091	0.500277	0.6102034
4540.	0.5651206	0.5866013	0.5347973	0.4962196	0.6095389
4550.	0.5598044	0.5820193	0.5327687	0.4928967	0.6046424
4560.	0.5595596	0.5839081	0.5347623	0.4895389	0.6058316
4570.	0.5584754	0.5831036	0.5333284	0.487755	0.6058316
4580.	0.5558522	0.5898542	0.5332584	0.4917774	0.6043976
4590.	0.5563769	0.5942264	0.5307402	0.4931416	0.6102034
4600.	0.5588601	0.5904489	0.5283968	0.489329	0.6116374
4610.	0.5587552	0.5842578	0.5225211	0.4890142	0.6154147
4620.	0.5590349	0.5816345	0.5196881	0.4881398	0.6104482
4630.	0.5567966	0.5806202	0.5180092	0.4859362	0.6114625
4640.	0.5566916	0.5817045	0.5181492	0.4840474	0.608035
4650.	0.5514804	0.5773323	0.5175896	0.4804098	0.606566
4660.	0.5506409	0.5702319	0.5201777	0.4778914	0.6092591
4670.	0.5479479	0.5691126	0.5174147	0.4774367	0.609399
4680.	0.549172	0.5665942	0.5163305	0.4756878	0.6088744
4690.	0.5447302	0.5663144	0.5185689	0.4727847	0.6098537
4700.	0.5425967	0.5681682	0.5167851	0.470651	0.6036281
4710.	0.5401834	0.5679584	0.5146866	0.4742537	0.6021242
4720.	0.5381898	0.5662445	0.515631	0.4730645	0.5977873
4730.	0.5374554	0.5645306	0.514232	0.4727847	0.5880992
4740.	0.5333633	0.5604382	0.5160507	0.4763524	0.5865253
4750.	0.5342027	0.5587243	0.512763	0.4747434	0.585616
4760.	0.537910	0.5579198	0.514197	0.4719802	0.5850214
4770.	0.5347273	0.5554014	0.5150014	0.4706161	0.5842869
4780.	0.5298308	0.5586544	0.5196881	0.469112	0.5840771
4790.	0.5297958	0.5604382	0.5240949	0.4661739	0.5846717
4800.	0.5273826	0.5601934	0.526823	0.4610672	0.5816289
4810.	0.5261934	0.5581297	0.5248994	0.4597731	0.582818
4820.	0.524025	0.5548768	0.5254589	0.4571847	0.5853711
4830.	0.5288166	0.5579898	0.5233954	0.4589336	0.5859308
4840.	0.5275924	0.5589342	0.5218915	0.4580242	0.5860007
4850.	0.5265781	0.5590391	0.5175546	0.4618017	0.5847066
4860.	0.5291314	0.5616624	0.5212969	0.4593883	0.5840071
4870.	0.5315096	0.5579198	0.5186388	0.4549462	0.585511
4880.	0.5301806	0.5569404	0.5216467	0.4509937	0.5811392
4890.	0.5328387	0.5554714	0.5257388	0.4479856	0.5760328
4900.	0.5286766	0.557570	0.5287466	0.4461668	0.5776417
4910.	0.5288865	0.5618722	0.5289914	0.447461	0.5796353
4920.	0.5307751	0.559179	0.5262284	0.4456072	0.5777816
4930.	0.5367559	0.5595288	0.5259836	0.4447677	0.5705417
4940.	0.5422819	0.5588292	0.5231506	0.444383	0.567394

4950.	0.5401484	0.559179	0.5210171	0.4436484	0.5641763
4960.	0.5427366	0.5586544	0.5220314	0.4446278	0.5621127
4970.	0.5401135	0.5598436	0.5152463	0.4470762	0.5626374
4980.	0.5356717	0.557605	0.5115739	0.4422493	0.5616581
4990.	0.5381199	0.559179	0.5120285	0.441235	0.5598744
5000.	0.5396239	0.559144	0.5094404	0.4395211	0.5628473
5010.	0.5450799	0.5600185	0.5090907	0.4398009	0.5593847
5020.	0.5452898	0.5580947	0.5110143	0.4364081	0.558965
5030.	0.5400085	0.5578848	0.5129029	0.4398359	0.5555725
5040.	0.538015	0.5583395	0.5117137	0.4391014	0.5532991
5050.	0.5390992	0.5572902	0.5088808	0.4369328	0.5488572
5060.	0.5438558	0.5565557	0.5067823	0.4354637	0.5517252
5070.	0.5356017	0.5572902	0.5092655	0.4323857	0.5532291
5080.	0.5382948	0.5572553	0.5045089	0.4324557	0.5488572
5090.	0.5370007	0.557605	0.5096152	0.4282933	0.5509208
5100.	0.537980	0.5575351	0.509930	0.4297624	0.5494168
5110.	0.5441356	0.5613826	0.5164704	0.4327705	0.5524247
5120.	0.5451499	0.5594938	0.5162255	0.4343444	0.5521099
5130.	0.5427716	0.559214	0.5154561	0.4316512	0.5526345
5140.	0.5389593	0.5630265	0.5135325	0.4321758	0.5511656
5150.	0.5381549	0.5624319	0.5087759	0.4347292	0.5530193
5160.	0.5417923	0.563866	0.5046838	0.4359884	0.5504311
5170.	0.5382598	0.5619772	0.505838	0.438192	0.5532991
5180.	0.5345175	0.5619422	0.5075867	0.4407103	0.5522498
5190.	0.5354618	0.5660695	0.5074818	0.4407103	0.5470386
5200.	0.5303904	0.5676086	0.5125881	0.4424242	0.544975
5210.	0.5316845	0.5661745	0.5125881	0.4436135	0.5417224
5220.	0.5248294	0.5641108	0.5164004	0.4420395	0.5376303
5230.	0.5228708	0.5639709	0.5186738	0.4380171	0.5373854
5240.	0.5269279	0.5672588	0.5186738	0.4393812	0.5382948
5250.	0.528257	0.5657198	0.5204225	0.4418296	0.5356017
5260.	0.5269979	0.5606131	0.5173098	0.434974	0.5314047
5270.	0.5260186	0.5584445	0.5173797	0.4313714	0.5334682
5280.	0.5280122	0.5590391	0.5150014	0.4274189	0.5256338
5290.	0.5264732	0.5565207	0.5134276	0.4264745	0.5235353
5300.	0.525319	0.5553665	0.510035	0.4285032	0.523955
5310.	0.5246895	0.5535476	0.5066424	0.424096	0.5215767
5320.	0.5218216	0.5449432	0.5031099	0.4254252	0.5205275
5330.	0.5220664	0.5433692	0.5050685	0.4262297	0.5149664
5340.	0.5232205	0.5458526	0.500137	0.4251454	0.5160856
5350.	0.5202127	0.5402212	0.5036345	0.4252853	0.5205974
5360.	0.5149314	0.5396616	0.5022005	0.4262646	0.5194433
5370.	0.5147915	0.5429145	0.5005218	0.4244108	0.5122034
5380.	0.5130078	0.5419701	0.501536	0.4268593	0.5092655
5390.	0.5109093	0.5325612	0.5005218	0.4218925	0.5116438
5400.	0.5069572	0.5338203	0.4992976	0.4252853	0.514127
5410.	0.504299	0.5320365	0.5002419	0.4295525	0.5125532
5420.	0.5023055	0.5292033	0.5027252	0.4283283	0.5121335
5430.	0.5005917	0.5322114	0.4994026	0.4263696	0.5129729
5440.	0.4994026	0.5286787	0.5040892	0.428888	0.5096152
5450.	0.5009764	0.5282939	0.5103498	0.4279785	0.5145118
5460.	0.5002419	0.5253208	0.5074818	0.4315812	0.5109093
5470.	0.4962198	0.5240266	0.5054182	0.4325606	0.5124483
5480.	0.4970592	0.5241666	0.5022355	0.4312664	0.5143019
5490.	0.4995075	0.5178356	0.4989479	0.4280835	0.5152113
5500.	0.503005	0.5151774	0.4948208	0.4297974	0.5123084

5510.	0.5051035	0.5176258	0.4902041	0.4267543	0.5087059
5520.	0.5080414	0.5214034	0.4905539	0.4260198	0.5083562
5530.	0.5076217	0.5171711	0.4895396	0.4281884	0.5005568
5540.	0.5025853	0.5181155	0.4860421	0.4309166	0.4974789
5550.	0.4981085	0.517276	0.4879657	0.4290978	0.4920578
5560.	0.4976888	0.5158419	0.4868115	0.4264046	0.4905888
5570.	0.4950307	0.5187801	0.4866367	0.4204584	0.4908337
5580.	0.4940514	0.5183253	0.4843283	0.4189194	0.4935617
5590.	0.4924076	0.5113998	0.4796767	0.4249355	0.4919179
5600.	0.4929322	0.5111899	0.4756895	0.422592	0.4893647
5610.	0.4910085	0.5075873	0.4724718	0.4181499	0.4913933
5620.	0.4905189	0.503390	0.4733112	0.4170306	0.4920228
5630.	0.4898544	0.5011514	0.4769836	0.417905	0.4937016
5640.	0.4921277	0.5025505	0.4804461	0.414862	0.4964647
5650.	0.4941213	0.4991577	0.4793619	0.4140925	0.4963247
5660.	0.4927223	0.5011514	0.4818451	0.4150019	0.4937016
5670.	0.4927573	0.5024806	0.4776831	0.4170306	0.4909386
5680.	0.4865667	0.5055935	0.4756545	0.4187795	0.4883854
5690.	0.4845732	0.5059433	0.4772634	0.4170306	0.4852377
5700.	0.4842584	0.5039146	0.4759343	0.4140925	0.4881406
5710.	0.4831042	0.5023406	0.472227	0.4164709	0.4916381
5720.	0.4836288	0.5054536	0.4702334	0.4154566	0.4908687
5730.	0.4838737	0.5023406	0.4676103	0.4174503	0.4885253
5740.	0.4859372	0.5015711	0.4677502	0.4180799	0.4887352
5750.	0.4879307	0.498668	0.4683098	0.4175552	0.487546
5760.	0.4905189	0.493946	0.4692541	0.4145472	0.4888751
5770.	0.4909386	0.4921972	0.4689743	0.414827	0.490379
5780.	0.4897844	0.4894689	0.4650221	0.414862	0.4881056
5790.	0.4887002	0.4907281	0.4668758	0.4100701	0.4860421
5800.	0.4857273	0.4913227	0.4646024	0.4142324	0.4873012
5810.	0.486252	0.4860061	0.4645325	0.4122037	0.4831741
5820.	0.4864618	0.4863209	0.4654418	0.414897	0.4854475
5830.	0.4836988	0.4859362	0.4641477	0.4151068	0.4845382
5840.	0.4880707	0.4931066	0.4653019	0.4127633	0.4827195
5850.	0.4869165	0.4942958	0.4642876	0.4116091	0.4854475
5860.	0.4878958	0.4888043	0.4627837	0.4090208	0.4880707
5870.	0.487546	0.4882797	0.4629586	0.4033194	0.4897145
5880.	0.4891899	0.4888043	0.458132	0.4006961	0.4836638
5890.	0.4877209	0.4896788	0.4579572	0.3998916	0.4809008
5900.	0.4844333	0.4913927	0.4550193	0.399262	0.4845732
5910.	0.4912184	0.4915326	0.4513469	0.3983176	0.4812855
5920.	0.4930371	0.4918474	0.4484439	0.3997517	0.4824047
5930.	0.4937716	0.4922671	0.4463454	0.4020952	0.480621
5940.	0.4920928	0.4926868	0.4422884	0.4048235	0.4807959
5950.	0.4903091	0.4891541	0.4369721	0.4061176	0.4813205
5960.	0.4884204	0.4896438	0.435783	0.4025849	0.4796767
5970.	0.4881056	0.4855864	0.4352933	0.3987024	0.4781727
5980.	0.4857973	0.4871604	0.4344889	0.3951347	0.4770535
5990.	0.4878608	0.4852366	0.4320407	0.3961141	0.4776131
6000.	0.4908337	0.4832779	0.4319358	0.3955194	0.4753048
6010.	0.4947159	0.4875452	0.4278087	0.3935257	0.4710728
6020.	0.4928273	0.4884895	0.4244161	0.4007311	0.4705482
6030.	0.4899243	0.4859362	0.4276338	0.3996118	0.4745004
6040.	0.4910785	0.4859712	0.4292077	0.3990522	0.4770885
6050.	0.4877559	0.487860	0.428753	0.3985275	0.4742555
6060.	0.4866717	0.4869156	0.4296974	0.3992271	0.4764589

6070.	0.4887702	0.4896088	0.4250107	0.3968836	0.4755496
6080.	0.4913933	0.4883496	0.4177709	0.396289	0.4744304
6090.	0.4919179	0.489329	0.4187852	0.3973383	0.4761792
6100.	0.4973041	0.4874052	0.4178758	0.3988073	0.4762841
6110.	0.4966395	0.4856914	0.4129793	0.3985625	0.472192
6120.	0.494541	0.487825	0.4073483	0.3974432	0.4737659
6130.	0.4954154	0.4871954	0.4073483	0.3920567	0.4694639
6140.	0.4951356	0.4916375	0.412035	0.3910074	0.4757944
6150.	0.4935617	0.4982832	0.4136088	0.3974782	0.4766338
6160.	0.4920578	0.5069227	0.4132241	0.394680	0.4742555
6170.	0.4925475	0.5029702	0.4111256	0.3971984	0.4716324
6180.	0.4965696	0.4988079	0.4071734	0.3952746	0.469394
6190.	0.4972341	0.4993326	0.4097966	0.3929311	0.4704782
6200.	0.4951006	0.4997523	0.4097616	0.3873347	0.4694989
6210.	0.4971642	0.4970241	0.4088173	0.3820181	0.470758
6220.	0.493212	0.4918124	0.4067537	0.3812137	0.4715974
6230.	0.4900642	0.4921272	0.4033262	0.3813536	0.4655118
6240.	0.4899943	0.4884546	0.4015425	0.3790101	0.4655118
6250.	0.4862869	0.4836277	0.4009479	0.3792199	0.458237
6260.	0.4873012	0.479990	0.405040	0.3814235	0.4562084
6270.	0.4870214	0.4805847	0.4060892	0.3810388	0.4546695
6280.	0.4823348	0.4756878	0.4033961	0.3830325	0.4537252
6290.	0.483384	0.4740788	0.4088173	0.3809339	0.4535853
6300.	0.4794668	0.4778214	0.4058444	0.3824728	0.4488637
6310.	0.4793968	0.4766322	0.407768	0.382228	0.4472898
6320.	0.4784525	0.4773667	0.4051099	0.377506	0.4478844
6330.	0.4795717	0.4767371	0.4075582	0.3768415	0.4445617
6340.	0.473556	0.4762474	0.4079429	0.3777509	0.4447716
6350.	0.4711777	0.4795353	0.4053897	0.3790451	0.4424632
6360.	0.4692541	0.4828582	0.4047252	0.3861455	0.4491784
6370.	0.4712477	0.4807595	0.4075931	0.3865303	0.4490035
6380.	0.4711078	0.4825784	0.4115803	0.3845015	0.4442469
6390.	0.4733812	0.4806196	0.4146931	0.3863903	0.4460657
6400.	0.4729265	0.477017	0.414798	0.3876845	0.4458208
6410.	0.4699186	0.4792555	0.4123847	0.383837	0.4453661
6420.	0.4704432	0.4821586	0.4143083	0.3827177	0.445611
6430.	0.4712827	0.4839775	0.4143083	0.3770863	0.4429179
6440.	0.470863	0.4856914	0.4156024	0.3803392	0.4457159
6450.	0.4731713	0.4807245	0.4124897	0.3812836	0.4424982
6460.	0.4775782	0.4788707	0.4171413	0.3823679	0.4438622
6470.	0.480516	0.4801649	0.4171063	0.3862154	0.4449465
6480.	0.4826145	0.481634	0.4214433	0.3850612	0.4438972
6490.	0.4836288	0.4832779	0.4206038	0.382228	0.4387209
6500.	0.4852726	0.484747	0.4140635	0.3817383	0.4394903
6510.	0.4855874	0.4805497	0.416162	0.3846764	0.4421484
6520.	0.4816702	0.4805147	0.4150428	0.3834872	0.4457859
6530.	0.4786974	0.4803048	0.4137138	0.3771563	0.4397352
6540.	0.4748851	0.4820537	0.411965	0.3768765	0.4394903
6550.	0.475060	0.4848169	0.4145532	0.3710702	0.4406795
6560.	0.4755496	0.4860411	0.4112655	0.371315	0.4363776
6570.	0.4717023	0.4843272	0.4155675	0.3699159	0.4360628
6580.	0.4658615	0.4820187	0.4122798	0.3684469	0.4346638
6590.	0.4597409	0.4808645	0.4104261	0.3650191	0.4323205
6600.	0.4599158	0.4804797	0.4089222	0.3672577	0.4344889
6610.	0.4600557	0.483173	0.4057744	0.3659985	0.4373569
6620.	0.4596709	0.4781712	0.4047602	0.3689716	0.4366574

6630.	0.4556488	0.4788707	0.3997238	0.3661034	0.435783
6640.	0.4536552	0.4777865	0.402172	0.3607868	0.4363776
6650.	0.451242	0.4768071	0.4046902	0.3606819	0.437217
6660.	0.4495282	0.4748483	0.4058444	0.3614864	0.4393854
6670.	0.4480243	0.4760026	0.4015075	0.362046	0.4388958
6680.	0.4499129	0.4747784	0.4044104	0.3645294	0.4393505
6690.	0.4458908	0.4757228	0.4031163	0.3694962	0.4397002
6700.	0.4443169	0.4740788	0.4013326	0.371385	0.4402248
6710.	0.4422534	0.4767371	0.3976952	0.3676074	0.438511
6720.	0.4406795	0.4742188	0.3968208	0.3694612	0.4369372
6730.	0.4419036	0.4794304	0.3915746	0.3707904	0.4389657
6740.	0.4391756	0.4807945	0.3929036	0.3703007	0.4348387
6750.	0.4398751	0.4820887	0.3868879	0.3704756	0.4371121
6760.	0.4339993	0.4864609	0.3850692	0.371420	0.4383712
6770.	0.4360278	0.4851317	0.3865732	0.3727841	0.4421484
6780.	0.4387909	0.4796053	0.3810821	0.3723993	0.4433026
6790.	0.4392106	0.4781013	0.3808722	0.3732388	0.4455061
6800.	0.4433376	0.4839425	0.3795781	0.3732388	0.4449465
6810.	0.4487238	0.4828582	0.378424	0.3718397	0.4461706
6820.	0.4480592	0.480060	0.3803476	0.3726092	0.4481292
6830.	0.4448065	0.4822286	0.3796831	0.3697061	0.4457859
6840.	0.4461706	0.4809694	0.3772348	0.3724693	0.4423933
6850.	0.4440721	0.4817739	0.3796131	0.3740083	0.4459607
6860.	0.4474297	0.4836977	0.3800678	0.3749177	0.4481642
6870.	0.4463804	0.4818788	0.3763604	0.3764917	0.4488637
6880.	0.4457509	0.4850618	0.3786688	0.3756872	0.4467302
6890.	0.4464854	0.4806546	0.3783191	0.3754424	0.4466602
6900.	0.4442819	0.4783111	0.3791584	0.3748128	0.4495632
6910.	0.4424982	0.4778214	0.3799279	0.3742881	0.4502976
6920.	0.4435125	0.4733093	0.3794033	0.3751975	0.4463454
6930.	0.4397702	0.4725748	0.3751363	0.3749177	0.4438273
6940.	0.4444218	0.4735542	0.3761156	0.3728541	0.4435125
6950.	0.4440021	0.4759676	0.3762555	0.3747778	0.4400499
6960.	0.4430928	0.4774017	0.3752063	0.3742532	0.4363076
6970.	0.439980	0.4747784	0.3730728	0.3716298	0.4321456
6980.	0.4359929	0.4731694	0.3729329	0.3699859	0.4365525
6990.	0.4350485	0.4699515	0.3731078	0.3689366	0.4342091
7000.	0.4345239	0.4733443	0.3704497	0.3703007	0.4335096
7010.	0.4350835	0.4709658	0.3693305	0.3694612	0.4367623
7020.	0.4347687	0.4664538	0.3733176	0.368307	0.4349086
7030.	0.434314	0.4646349	0.3709044	0.3712451	0.4338244
7040.	0.4326702	0.4635156	0.3704147	0.3696711	0.4332648
7050.	0.4321456	0.4614869	0.3707295	0.3657187	0.4318308
7060.	0.432985	0.4642502	0.3738772	0.3641796	0.4311663
7070.	0.4302919	0.4628161	0.3721984	0.3677473	0.4305367
7080.	0.4317609	0.4565201	0.3749265	0.3697411	0.4291028
7090.	0.4326352	0.4502942	0.3734225	0.3701608	0.4283683
7100.	0.4308515	0.4471462	0.3684561	0.3712101	0.4284732
7110.	0.4337894	0.4466915	0.3633497	0.3716998	0.4256752
7120.	0.4324604	0.447356	0.3620207	0.3664532	0.4284382
7130.	0.4276688	0.4447328	0.3619857	0.365229	0.4291028
7140.	0.4256403	0.4421794	0.3636295	0.365159	0.4256403
7150.	0.4241363	0.4437534	0.3645389	0.3633052	0.424556
7160.	0.421863	0.4446628	0.3660078	0.3688316	0.4262348
7170.	0.4209886	0.4468314	0.3638394	0.3685168	0.4248708
7180.	0.4248008	0.4491749	0.3620906	0.371420	0.4269693

7190.	0.424661	0.4482655	0.3621956	0.3693213	0.4252905
7200.	0.4248358	0.4455023	0.3625453	0.3670128	0.423227
7210.	0.4251156	0.4479856	0.3642941	0.3649142	0.4211285
7220.	0.4238215	0.444383	0.3638394	0.3601922	0.418960
7230.	0.4224575	0.4459919	0.3652034	0.3599823	0.4190649
7240.	0.4213033	0.4446628	0.3648187	0.3564496	0.4197295
7250.	0.4277038	0.4407803	0.3677216	0.3532317	0.4149029
7260.	0.4264447	0.4401157	0.3677916	0.3525671	0.4142384
7270.	0.4264097	0.4375624	0.3690856	0.3528819	0.4125946
7280.	0.4305018	0.438157	0.3675118	0.3506783	0.4122098
7290.	0.4271442	0.4415148	0.3684911	0.3498739	0.4159172
7300.	0.4269343	0.4408153	0.3646438	0.3482649	0.4123148
7310.	0.4266196	0.4405704	0.3622305	0.3470407	0.4137837
7320.	0.4252205	0.4368628	0.3606916	0.341864	0.416232
7330.	0.4222826	0.4357435	0.3583483	0.340325	0.4125946
7340.	0.4170714	0.4343095	0.3560749	0.335673	0.4123847
7350.	0.4139586	0.4327005	0.3571592	0.3379815	0.4082927
7360.	0.4149729	0.431861	0.3565296	0.3386111	0.4042705
7370.	0.4157073	0.4251104	0.3538365	0.341934	0.4006331
7380.	0.4145182	0.4236413	0.3513883	0.3435429	0.4011577
7390.	0.407768	0.4232916	0.3510036	0.3447672	0.4025567
7400.	0.4059843	0.4244458	0.3495346	0.3455017	0.399444
7410.	0.4080828	0.4221373	0.3500942	0.3475654	0.3991642
7420.	0.4090971	0.4228019	0.3477159	0.343438	0.3992341
7430.	0.4095517	0.4254602	0.3496395	0.3454667	0.3957716
7440.	0.4130842	0.4249355	0.3530671	0.3478802	0.3960164
7450.	0.4102862	0.4246907	0.3524375	0.349594	0.3977652
7460.	0.4122798	0.4173104	0.3495346	0.3471806	0.3955268
7470.	0.4123497	0.4174503	0.3466667	0.343438	0.390980
7480.	0.413399	0.4198288	0.3528922	0.3439977	0.3892662
7490.	0.406334	0.4161561	0.3547109	0.3429133	0.3874125
7500.	0.4042355	0.4094055	0.3549208	0.3421438	0.3876224
7510.	0.4018223	0.4073418	0.3571592	0.3411645	0.3893012
7520.	0.4034661	0.4027947	0.3586281	0.3407447	0.3875874
7530.	0.4006681	0.4015006	0.3558301	0.3429133	0.3841249
7540.	0.4035361	0.4015356	0.3597123	0.3424586	0.3847544
7550.	0.4014026	0.4054531	0.3568444	0.3455716	0.3838451
7560.	0.3992691	0.4029347	0.3564946	0.3457815	0.3792284
7570.	0.3976602	0.4018504	0.355970	0.3458515	0.3829008
7580.	0.3951071	0.4000665	0.3561449	0.3428434	0.3830057
7590.	0.3943376	0.4025149	0.3554454	0.3402201	0.3851742
7600.	0.3903505	0.4044037	0.3541513	0.3395905	0.3863983
7610.	0.3913997	0.4023051	0.3577188	0.3406748	0.3846146
7620.	0.3892313	0.4004163	0.3582434	0.3414443	0.3855939
7630.	0.3884268	0.405488	0.3539065	0.3425986	0.3875874
7640.	0.3915046	0.4079365	0.3529272	0.3422138	0.3870978
7650.	0.3914347	0.4075517	0.357404	0.3405699	0.3888116
7660.	0.3928337	0.4017454	0.3556552	0.3408847	0.3928687
7670.	0.3925539	0.4034943	0.3546759	0.3393456	0.391015
7680.	0.3916095	0.4002764	0.3529971	0.3415492	0.3898958
7690.	0.3939529	0.4011858	0.3528223	0.343438	0.3888815
7700.	0.3900357	0.4010809	0.3497445	0.3443824	0.3913997
7710.	0.3922041	0.3984925	0.3470864	0.3424236	0.3888465
7720.	0.3901406	0.402480	0.3474361	0.341899	0.3891263
7730.	0.3879022	0.4015706	0.3456874	0.3408497	0.3829358
7740.	0.388217	0.4027598	0.3429943	0.3394156	0.3837751

7750.	0.3934632	0.400836	0.3408258	0.3400802	0.3813969
7760.	0.3880071	0.3968136	0.3400913	0.3425286	0.3787038
7770.	0.3866781	0.3953446	0.3401963	0.345047	0.3775846
7780.	0.3868879	0.3943302	0.3412805	0.344942	0.3744018
7790.	0.3814318	0.3902378	0.3417002	0.3426335	0.3716738
7800.	0.3815717	0.391532	0.3429593	0.3402551	0.3674768
7810.	0.3842998	0.3933159	0.3428194	0.3391358	0.3620207
7820.	0.3827259	0.3927213	0.3396017	0.3416891	0.3646438
7830.	0.3809072	0.3906576	0.3374682	0.3426685	0.3655881
7840.	0.3762905	0.3849562	0.3381677	0.3408847	0.3649936
7850.	0.3753112	0.3872997	0.3394268	0.3384013	0.364434
7860.	0.3776545	0.3878594	0.3390421	0.3342389	0.3570892
7870.	0.3768851	0.3845715	0.3375382	0.3328398	0.3542213
7880.	0.379858	0.3867751	0.3375032	0.3314407	0.3522976
7890.	0.3808373	0.3834872	0.3359293	0.3324551	0.3524725
7900.	0.3794732	0.3834522	0.3371185	0.3359878	0.3519129
7910.	0.379858	0.3827177	0.3372934	0.3367923	0.3543262
7920.	0.3809422	0.3766316	0.334950	0.3385412	0.351773
7930.	0.3842998	0.3811787	0.3305432	0.3374219	0.3497445
7940.	0.3881121	0.380689	0.3312077	0.3396254	0.3467716
7950.	0.3894411	0.3833473	0.3301584	0.3384362	0.3444632
7960.	0.3913647	0.3841518	0.3270457	0.3358129	0.3394268
7970.	0.3927287	0.3813536	0.3269058	0.3396954	0.334915
7980.	0.392449	0.3798146	0.3286895	0.3359878	0.3350899
7990.	0.3877273	0.3833823	0.3257166	0.335638	0.3320821
8000.	0.3874475	0.3858307	0.3247373	0.3367223	0.3340757
8010.	0.3874825	0.3850262	0.3231984	0.3370721	0.3332362
8020.	0.3873426	0.3834522	0.3268358	0.3320354	0.3309979
8030.	0.3888116	0.3836621	0.3252969	0.331091	0.3280599
8040.	0.3863283	0.3849213	0.3251221	0.3326649	0.329389
8050.	0.382551	0.3825428	0.3266609	0.331021	0.329354
8060.	0.3826559	0.3815635	0.3240728	0.3319304	0.3308929
8070.	0.3757659	0.3825078	0.3228836	0.3296219	0.3319072
8080.	0.3753811	0.3784155	0.3241777	0.3348335	0.3292491
8090.	0.3760806	0.376072	0.3240378	0.3352183	0.3299836
8100.	0.3726881	0.3750576	0.3216595	0.3350084	0.330858
8110.	0.3747166	0.3710352	0.3202955	0.3348685	0.3297038
8120.	0.3726181	0.3730289	0.3236531	0.3353582	0.330823
8130.	0.3673019	0.3701258	0.3230236	0.3361277	0.3255767
8140.	0.3625104	0.3689016	0.3216245	0.3337842	0.3198408
8150.	0.3589429	0.368272	0.3217994	0.3326999	0.3178822
8160.	0.3570542	0.3711751	0.3219393	0.3293071	0.3180571
8170.	0.3610414	0.372959	0.3218694	0.3305663	0.3201556
8180.	0.3667074	0.3703007	0.318197	0.3304264	0.3192113
8190.	0.3645739	0.3679222	0.3158187	0.3269636	0.318197
8200.	0.3619507	0.3677473	0.3147345	0.3245851	0.3156088
8210.	0.3596424	0.3672227	0.3095931	0.3236757	0.318232
8220.	0.3619158	0.3662083	0.3096981	0.3256694	0.3202955
8230.	0.3571941	0.3679222	0.3119714	0.3240255	0.3206452
8240.	0.3534518	0.3715249	0.3147694	0.3236408	0.3165532
8250.	0.3553055	0.3681671	0.3137202	0.3224515	0.3101178
8260.	0.3530671	0.3666281	0.3108522	0.3223466	0.3115867
8270.	0.3536966	0.3683769	0.3117266	0.3162605	0.3107473
8280.	0.357369	0.3613465	0.3116916	0.3193035	0.3113069
8290.	0.3592227	0.3595626	0.3152241	0.3211224	0.3130207
8300.	0.3607266	0.3577438	0.3146645	0.3233959	0.3167281

8310.	0.3600271	0.3586182	0.3135453	0.3222067	0.3152241
8320.	0.3580335	0.357394	0.3119015	0.3272434	0.3152241
8330.	0.3548858	0.355785	0.315329	0.3259842	0.3170078
8340.	0.354606	0.352777	0.3117266	0.3243053	0.3165532
8350.	0.3490449	0.3489645	0.3076695	0.3205278	0.3145946
8360.	0.3501292	0.3454317	0.3081941	0.3236408	0.3163083
8370.	0.3494996	0.3443474	0.3055011	0.3229762	0.3186866
8380.	0.3467716	0.3438927	0.3049065	0.3206677	0.3187566
8390.	0.3456174	0.3374219	0.3044168	0.3207376	0.3236881
8400.	0.3440435	0.3329448	0.3021085	0.320178	0.3235482
8410.	0.3460371	0.3317555	0.2977716	0.3228713	0.3235482
8420.	0.3446031	0.332560	0.2995903	0.3218219	0.3221492
8430.	0.3439736	0.329412	0.2996602	0.3209475	0.3208551
8440.	0.3400913	0.3242354	0.2957081	0.3197932	0.3174975
8450.	0.3371884	0.3247251	0.2950785	0.3191287	0.3206802
8460.	0.3357545	0.3256345	0.2939943	0.3204928	0.3201206
8470.	0.3382027	0.3280129	0.2942741	0.3227313	0.3177423
8480.	0.3442534	0.3269986	0.2965824	0.3190937	0.3149793
8490.	0.3446731	0.3251798	0.2964775	0.315421	0.3158886
8500.	0.3457923	0.3267188	0.2928751	0.3136722	0.3160985
8510.	0.3482405	0.324760	0.2914061	0.3129027	0.3176024
8520.	0.3465617	0.3223816	0.2898323	0.3098596	0.3190014
8530.	0.346177	0.3226264	0.2913012	0.3074112	0.3180221
8540.	0.3441135	0.3242354	0.2885731	0.3058372	0.3158886
8550.	0.3469115	0.3218569	0.2884333	0.3052426	0.3154689
8560.	0.3462819	0.3182892	0.2914061	0.3028991	0.3133355
8570.	0.3482056	0.317100	0.2922105	0.3018148	0.3134404
8580.	0.3502341	0.3158758	0.2926652	0.3003108	0.3108173
8590.	0.3507238	0.3152812	0.2907766	0.2989117	0.3122512
8600.	0.3520178	0.3128327	0.2938544	0.2967781	0.3115867
8610.	0.3540464	0.3103843	0.2922805	0.2946444	0.3141749
8620.	0.3578936	0.3130426	0.2924204	0.2941897	0.3137901
8630.	0.3603419	0.312413	0.2936096	0.2919512	0.3102577
8640.	0.3577537	0.3145816	0.2928751	0.2939099	0.309733
8650.	0.358873	0.3161206	0.2938194	0.2859001	0.3113069
8660.	0.3594675	0.3176946	0.2941342	0.2858651	0.3128808
8670.	0.3605517	0.3188139	0.2932948	0.2852705	0.3132305
8680.	0.3577537	0.317030	0.2966524	0.2846059	0.3144896
8690.	0.3589079	0.3175197	0.2960928	0.2864247	0.311132
8700.	0.3593626	0.3165053	0.292945	0.2841862	0.3095232
8710.	0.3589079	0.3167152	0.2927002	0.2812831	0.3068651
8720.	0.3569493	0.3172049	0.2938194	0.2771207	0.3074247
8730.	0.3554454	0.320143	0.2935396	0.2768759	0.3035774
8740.	0.3526824	0.3220668	0.2946238	0.2739378	0.3044168
8750.	0.3515981	0.3194785	0.2984011	0.2727486	0.300010
8760.	0.3536966	0.3194085	0.2949736	0.2737979	0.2994154
8770.	0.3533819	0.3209125	0.2966874	0.2771907	0.2975967
8780.	0.3515632	0.320143	0.2948686	0.2762463	0.2970721
8790.	0.3510036	0.3211923	0.2954632	0.2746024	0.2960228
8800.	0.3494647	0.3173448	0.2978415	0.2762813	0.2940642
8810.	0.3520528	0.3183941	0.2965824	0.2749871	0.2914411
8820.	0.3541164	0.3194785	0.2970021	0.2749871	0.2862648
8830.	0.3586631	0.3193385	0.2991706	0.272119	0.2829072
8840.	0.3549907	0.320108	0.3040671	0.2686212	0.2820328
8850.	0.355900	0.3207026	0.306935	0.2697755	0.2778009
8860.	0.3547109	0.3163305	0.3090685	0.2672221	0.2749329

8870.	0.3526474	0.3183592	0.3066552	0.2647037	0.273429
8880.	0.3548858	0.3223466	0.3045218	0.2633746	0.2707709
8890.	0.3557951	0.3190587	0.3029829	0.2608212	0.2709457
8900.	0.3571592	0.3196533	0.3016188	0.2609611	0.2726595
8910.	0.3543262	0.3204928	0.2988208	0.2600867	0.2709457
8920.	0.3487302	0.3212623	0.2987159	0.2620804	0.2696167
8930.	0.350444	0.3211224	0.3010243	0.264354	0.2680078
8940.	0.348940	0.3167502	0.2945889	0.2645288	0.267798
8950.	0.3464918	0.3164004	0.2936795	0.2644939	0.2680428
8960.	0.3460021	0.3175897	0.294414	0.2622903	0.2670285
8970.	0.3491149	0.315456	0.2970721	0.2603665	0.2665039
8980.	0.3494996	0.3127278	0.2988908	0.2582329	0.2669586
8990.	0.3456174	0.313987	0.2973169	0.2623602	0.269232
9000.	0.341980	0.3148614	0.2964775	0.262675	0.2670985
9010.	0.3415953	0.3143717	0.2958829	0.2643889	0.2711556
9020.	0.341945	0.3184991	0.2961977	0.2636194	0.271995
9030.	0.3408258	0.3183941	0.2971071	0.2637244	0.2711206
9040.	0.3396017	0.3166453	0.2967573	0.2623602	0.2711206
9050.	0.3383776	0.315561	0.2982263	0.2616257	0.2696517
9060.	0.3351949	0.3137072	0.2956731	0.2604365	0.2672384
9070.	0.3340757	0.3141619	0.2968273	0.2545603	0.263601
9080.	0.3352998	0.3120282	0.2940293	0.2524616	0.2618173
9090.	0.3305432	0.3158408	0.2898672	0.250293	0.2585296
9100.	0.3311028	0.3176246	0.2949736	0.2488939	0.2590193
9110.	0.329354	0.3177296	0.2971071	0.250293	0.2541577
9120.	0.3304383	0.3148614	0.2987159	0.250328	0.256501
9130.	0.3283398	0.3172399	0.297177	0.2538257	0.2571656
9140.	0.3271506	0.3195484	0.2967223	0.2513773	0.2577951
9150.	0.3220792	0.3157708	0.2981213	0.2535809	0.2576902
9160.	0.3234432	0.3144417	0.3014789	0.2501531	0.2577602
9170.	0.3235482	0.3119933	0.3001499	0.2475298	0.2533533
9180.	0.3247024	0.3097897	0.2982962	0.245606	0.2537031
9190.	0.3214847	0.3106291	0.3025632	0.2435074	0.2536681
9200.	0.3241777	0.3122731	0.3042769	0.2420034	0.2553119
9210.	0.3249821	0.3088103	0.3017237	0.240954	0.2556966
9220.	0.3257516	0.3055924	0.2992405	0.2393101	0.2525139
9230.	0.3281998	0.3038085	0.2994504	0.2395549	0.2477223
9240.	0.3295289	0.3072713	0.300045	0.239415	0.2486317
9250.	0.3278851	0.3083906	0.3015489	0.2413388	0.2476174
9260.	0.326521	0.3032839	0.3011991	0.2370016	0.2466731
9270.	0.329459	0.3028641	0.3006745	0.2371765	0.2487016
9280.	0.3313126	0.3012552	0.3033676	0.2365469	0.2494361
9290.	0.3351599	0.3023395	0.3008494	0.2351128	0.2514996
9300.	0.3344953	0.3020597	0.2981913	0.2311603	0.252444
9310.	0.3364889	0.3011503	0.2960578	0.2307056	0.2505203
9320.	0.3357545	0.3026193	0.2939593	0.2280124	0.2527238
9330.	0.3337609	0.3084605	0.2956031	0.2278375	0.2520592
9340.	0.3333062	0.304683	0.2941692	0.2290617	0.2512898
9350.	0.3327816	0.3068516	0.2941692	0.2331191	0.2525139
9360.	0.3320471	0.3056274	0.2937145	0.2350428	0.2482819
9370.	0.3358594	0.3054875	0.2920007	0.2322796	0.2470228
9380.	0.3362441	0.3070964	0.290217	0.233224	0.2457288
9390.	0.3365938	0.3043332	0.2910914	0.2344133	0.2454489
9400.	0.3376431	0.3033189	0.2886781	0.2349029	0.2477223
9410.	0.3380628	0.3049628	0.2895525	0.233224	0.2485967
9420.	0.3393219	0.3037386	0.2897973	0.2340984	0.2462884

9430.	0.3397066	0.3033189	0.2917908	0.2349729	0.2461135
9440.	0.3382027	0.3005207	0.292910	0.2348679	0.246813
9450.	0.3369086	0.2994713	0.2897623	0.233189	0.249541
9460.	0.3394968	0.3024094	0.2922105	0.2335388	0.2484568
9470.	0.3369786	0.2985269	0.2960928	0.2373164	0.2485617
9480.	0.3401263	0.2974426	0.2952184	0.2370715	0.2473726
9490.	0.3386923	0.2979323	0.2956381	0.2387504	0.2479672
9500.	0.3367338	0.298422	0.2931199	0.2380509	0.2513947
9510.	0.335020	0.2994364	0.2926303	0.2413038	0.2521292
9520.	0.3356146	0.2964633	0.2933997	0.244102	0.2491213
9530.	0.3351599	0.2971278	0.292980	0.2409191	0.2489465
9540.	0.3348101	0.2988417	0.2912312	0.2382608	0.2506952
9550.	0.3305082	0.2982121	0.2917908	0.234728	0.2516395
9560.	0.3323968	0.2960435	0.2936096	0.2356375	0.2544025
9570.	0.3366638	0.2955189	0.2917908	0.239485	0.2548922
9580.	0.3373633	0.2980023	0.2923504	0.2404643	0.2559065
9590.	0.3326417	0.2996812	0.2951485	0.2406742	0.2548572
9600.	0.3342156	0.2997861	0.2952884	0.2426679	0.2555917
9610.	0.3311727	0.2967081	0.2975267	0.2445217	0.2595089
9620.	0.3312077	0.2952041	0.2971071	0.244137	0.2602434
9630.	0.330753	0.2944696	0.3010243	0.2404993	0.2590193
9640.	0.3287944	0.2958687	0.3043469	0.2385056	0.2588094
9650.	0.3251221	0.2936651	0.3052562	0.2385756	0.2604183
9660.	0.3274654	0.2954839	0.3048715	0.2384357	0.260838
9670.	0.3311727	0.2986319	0.3046617	0.2393101	0.2597537
9680.	0.3346702	0.2980722	0.3071799	0.239415	0.2620271
9690.	0.3360342	0.2981422	0.3034375	0.2400796	0.2652099
9700.	0.3339357	0.2992615	0.3045917	0.2404993	0.263636
9710.	0.3313826	0.298387	0.3049414	0.239415	0.2619921
9720.	0.3318023	0.2991216	0.309803	0.2371415	0.2628665
9730.	0.3314875	0.2987368	0.3123562	0.2366518	0.2611527
9740.	0.3316973	0.3006606	0.314000	0.2343783	0.2592291
9750.	0.3249472	0.2976525	0.3127409	0.2340635	0.2589843
9760.	0.3267309	0.2917063	0.3128108	0.2320698	0.2583198
9770.	0.3268708	0.2925108	0.3121113	0.2288169	0.2562912
9780.	0.3215546	0.2907969	0.3070399	0.2259137	0.2559065
9790.	0.3203654	0.2911817	0.3095931	0.2266133	0.2556267
9800.	0.3173226	0.2917063	0.3053262	0.2273828	0.2555567
9810.	0.3145946	0.2950991	0.3032277	0.2264384	0.2528287
9820.	0.3177773	0.2932453	0.3003248	0.2250743	0.2503455
9830.	0.3173226	0.2908669	0.2983662	0.2216814	0.2491913
9840.	0.3180921	0.2917063	0.2987859	0.2242348	0.250940
9850.	0.3157837	0.2898875	0.2938194	0.2205272	0.2514297
9860.	0.3161335	0.2884184	0.2961278	0.2210519	0.2491563
9870.	0.3159586	0.2865646	0.2940992	0.220842	0.2456238
9880.	0.3192113	0.2881736	0.2936445	0.2212617	0.2424411
9890.	0.3200157	0.2870543	0.2939243	0.2236752	0.241182
9900.	0.316833	0.2858651	0.2969671	0.2247944	0.2409022
9910.	0.3172877	0.2881036	0.2983662	0.2263335	0.2416717
9920.	0.316798	0.2903422	0.2974568	0.2236402	0.2411121
9930.	0.3155739	0.2901673	0.2938544	0.2233953	0.2406224
9940.	0.3146645	0.2939449	0.2907416	0.2233953	0.2408672
9950.	0.3132655	0.2928956	0.2916859	0.2210868	0.2432455
9960.	0.3143847	0.2925108	0.2906716	0.2184985	0.2471278
9970.	0.3120414	0.2967431	0.291546	0.2183936	0.2460435
9980.	0.3117616	0.2944696	0.2928401	0.2190581	0.2462534

9990.	0.3148744	0.2950292	0.2925253	0.2218214	0.2480021

Gillespie simulation: 5 realisations for DW10

Gillespie simulations of the change in size of antigen-specific precursor populations within the long-lived T_{SCM} pool were performed for each individual. Five randomly chosen Gillespie simulations for DW10 are shown. These results are depicted in Fig 6B.

0.	1.000000	1.000000	1.000000	1.000000	1.000000
10.	0.997784	0.9972301	0.9980478	0.9980654	0.9982941
20.	0.995823	0.9954274	0.9960781	0.9954098	0.9963507
30.	0.994249	0.9924113	0.9934664	0.9930003	0.9940819
40.	0.9918219	0.990380	0.992165	0.9900369	0.9922265
50.	0.9900544	0.9887796	0.9904414	0.9874692	0.9903271
60.	0.9869943	0.9867835	0.9881023	0.9849191	0.9879616
70.	0.9850773	0.985359	0.9854906	0.9822106	0.9849806
80.	0.9831779	0.9837674	0.9836264	0.9804519	0.9829845
90.	0.9812346	0.9814196	0.9810587	0.9785701	0.981261
100.	0.9787196	0.9806545	0.9780161	0.9771016	0.9790362
110.	0.9762222	0.9786584	0.9757122	0.9751319	0.9775764
120.	0.9733643	0.9758446	0.9739007	0.9723004	0.9760288
130.	0.9716232	0.9744816	0.9724586	0.9706383	0.974191
140.	0.9697062	0.9711137	0.9701899	0.9686774	0.9721684
150.	0.968352	0.969909	0.9676837	0.9661712	0.9687213
160.	0.9673935	0.9669368	0.9656348	0.963155	0.9662064
170.	0.9647906	0.9657849	0.9639729	0.9619679	0.9637354
180.	0.9628384	0.963525	0.9628825	0.9597871	0.9614667
190.	0.9603763	0.9626545	0.9615018	0.9575184	0.9590749
200.	0.9581866	0.9595416	0.9591979	0.955320	0.9576503
210.	0.955575	0.9580907	0.9575008	0.9539658	0.9566126
220.	0.9524972	0.9559539	0.9553376	0.9520664	0.9550386
230.	0.9504923	0.9546964	0.9529369	0.9498152	0.9531656
240.	0.9497888	0.9531136	0.9511958	0.948206	0.9498592
250.	0.9483818	0.9512582	0.949059	0.9456207	0.9473091
260.	0.9480301	0.9483476	0.9471684	0.9437213	0.9452778
270.	0.9447501	0.9464658	0.9451283	0.9409865	0.9439587
280.	0.9429826	0.9445841	0.9430706	0.9390168	0.9412943
290.	0.9407051	0.9419196	0.9405996	0.9376889	0.9394212
300.	0.9398873	0.9399675	0.9390871	0.9361237	0.9379791
310.	0.9373723	0.9384199	0.9365634	0.934022	0.9352531
320.	0.9359741	0.9358962	0.9338638	0.9320523	0.9333361
330.	0.9352179	0.9334516	0.9313664	0.9301792	0.9324128
340.	0.9341891	0.9312357	0.9293878	0.9287547	0.931296
350.	0.9324127	0.929688	0.9281743	0.9266794	0.9295725
360.	0.9307508	0.9267686	0.9253955	0.924481	0.9277786
370.	0.9280951	0.9248956	0.9235049	0.9225552	0.9254747
380.	0.9261342	0.9228556	0.9218869	0.9210252	0.9237248
390.	0.9246832	0.9213343	0.9210076	0.9195479	0.9212362
400.	0.9232323	0.919136	0.9189147	0.9168922	0.9190818
410.	0.9215351	0.9187579	0.917411	0.915450	0.9173846
420.	0.9208141	0.9167354	0.9156435	0.9133396	0.9149224
430.	0.9186157	0.9158208	0.9140958	0.9112643	0.9136122
440.	0.9168922	0.9138336	0.9112291	0.9087406	0.9121965
450.	0.9146938	0.9122419	0.9104465	0.9059706	0.9099277
460.	0.9131285	0.9108614	0.9092594	0.9044141	0.9082921
470.	0.9114841	0.9091115	0.9078261	0.9031039	0.9058563

480.	0.9093825	0.9069835	0.9068148	0.9014771	0.9039832
490.	0.9079139	0.9050401	0.905188	0.8998942	0.9023564
500.	0.9055045	0.902833	0.9031655	0.897520	0.9017585
510.	0.9034468	0.9019097	0.9013012	0.8958844	0.900202
520.	0.9023564	0.8990343	0.9005098	0.8942049	0.8988391
530.	0.9005801	0.8979263	0.8979685	0.8924989	0.8976079
540.	0.8984169	0.8969854	0.8952864	0.8921824	0.895902
550.	0.897775	0.8945233	0.892578	0.8903357	0.8947764
560.	0.8951017	0.8932834	0.890494	0.8880054	0.8926747
570.	0.893343	0.8908125	0.889641	0.8859037	0.8921735
580.	0.8915316	0.8889219	0.888190	0.8834943	0.8909249
590.	0.8896233	0.8860201	0.8873459	0.8821049	0.8894827
600.	0.8874778	0.8843493	0.8847254	0.8803814	0.8871349
610.	0.8851299	0.8827489	0.8828348	0.878183	0.8853409
620.	0.8836262	0.880234	0.8810233	0.8760549	0.8849892
630.	0.8810672	0.8782379	0.8788689	0.874393	0.882993
640.	0.8798537	0.8765408	0.8765034	0.8729244	0.8822984
650.	0.8783149	0.8746063	0.8741379	0.8721067	0.8802143
660.	0.8764858	0.8735158	0.8723089	0.8705854	0.8787546
670.	0.8753603	0.8713175	0.8701193	0.8689498	0.8767848
680.	0.8738477	0.8700513	0.8679649	0.8658369	0.8755361
690.	0.8712537	0.8673429	0.865573	0.8634626	0.8728541
700.	0.8698115	0.8656809	0.8635857	0.862680	0.8720011
710.	0.8681935	0.8646873	0.8615456	0.8604816	0.8703303
720.	0.8651333	0.8624713	0.8594615	0.8584591	0.8679649
730.	0.8636208	0.8607215	0.8579227	0.8566212	0.8670768
740.	0.8612993	0.8590771	0.8567531	0.8544668	0.865872
750.	0.8591625	0.8578109	0.8550999	0.8530158	0.8643068
760.	0.8574126	0.856826	0.8531917	0.8517232	0.8626184
770.	0.8556979	0.8551201	0.851688	0.8497623	0.8612466
780.	0.8539479	0.8530272	0.8505097	0.848285	0.8589603
790.	0.8511164	0.851163	0.8486543	0.847344	0.8563046
800.	0.8497886	0.8496418	0.8469659	0.844917	0.8554693
810.	0.8488301	0.8484459	0.8445829	0.8425428	0.8535874
820.	0.8478452	0.8473379	0.8441784	0.840107	0.8500437
830.	0.8458754	0.8459662	0.842490	0.8383834	0.8474759
840.	0.8435628	0.8441371	0.8403444	0.8368885	0.8464999
850.	0.8409687	0.8421674	0.838146	0.8352793	0.8447675
860.	0.8391044	0.8394502	0.8363169	0.8333887	0.8435979
870.	0.8380932	0.8375069	0.8348836	0.8313925	0.8422877
880.	0.8368269	0.8355548	0.8333095	0.8296426	0.8393595
890.	0.8365104	0.8328552	0.8318586	0.828605	0.8381284
900.	0.8351913	0.8321869	0.831032	0.8273124	0.8356223
910.	0.8325357	0.8306041	0.8295195	0.8252019	0.8333535
920.	0.8311815	0.8285465	0.8280598	0.823962	0.8323422
930.	0.8290358	0.8258117	0.8264066	0.8220538	0.8301966
940.	0.8269078	0.823455	0.8242522	0.819618	0.8291238
950.	0.8250523	0.822013	0.8217636	0.8183869	0.8275937
960.	0.822511	0.8203158	0.8202687	0.8167776	0.8275234
970.	0.8207435	0.8183109	0.8181055	0.8155114	0.8270661
980.	0.8195564	0.8173437	0.8157664	0.8132163	0.8245336
990.	0.8177185	0.8162621	0.8144913	0.8116246	0.8228012
1000.	0.8161357	0.8149343	0.8121962	0.8097868	0.8205853
1010.	0.8152564	0.8130525	0.8108332	0.8086436	0.818765
1020.	0.8134713	0.8106782	0.8083358	0.8068761	0.8161797
1030.	0.8117301	0.8082249	0.8056275	0.8052318	0.8142012

1040.	0.8101561	0.8055869	0.8034818	0.8041149	0.8123457
1050.	0.8077642	0.8045405	0.801811	0.8024706	0.8101825
1060.	0.8064892	0.8033358	0.8008614	0.800281	0.8088195
1070.	0.8052141	0.800645	0.7987509	0.7983112	0.8069992
1080.	0.8027607	0.7984818	0.7970625	0.7969746	0.8055747
1090.	0.8007118	0.7963275	0.7950928	0.7956468	0.8043612
1100.	0.7991905	0.7946919	0.7929911	0.793079	0.8028575
1110.	0.7974846	0.7931003	0.7913995	0.7911357	0.8003601
1120.	0.794908	0.7912273	0.7902739	0.7886999	0.7980386
1130.	0.7931669	0.7899435	0.7886999	0.7881986	0.7960513
1140.	0.7915313	0.788941	0.7878381	0.7867565	0.7937649
1150.	0.7899925	0.7873494	0.7863608	0.7847691	0.791250
1160.	0.7888493	0.7861095	0.7842416	0.7833094	0.7900717
1170.	0.7888141	0.7835946	0.7839338	0.7824828	0.7880755
1180.	0.787838	0.7820821	0.7829665	0.7806714	0.785244
1190.	0.7866421	0.7804642	0.7816914	0.7790622	0.7837315
1200.	0.7847779	0.7794266	0.7799854	0.7779014	0.7816299
1210.	0.7831511	0.7774656	0.7789127	0.7766791	0.7791765
1220.	0.7812605	0.7760059	0.776767	0.7748588	0.7774969
1230.	0.7801701	0.7741153	0.7752897	0.7727835	0.7762834
1240.	0.7786752	0.7733503	0.7730297	0.7706291	0.7736189
1250.	0.7769956	0.7716268	0.7717811	0.7687913	0.7721152
1260.	0.775061	0.7699473	0.7701895	0.7672964	0.7704269
1270.	0.772933	0.7683381	0.7691342	0.7655992	0.7690902
1280.	0.7716404	0.7659903	0.7680351	0.7636646	0.7671469
1290.	0.769486	0.7649087	0.7660829	0.7612552	0.7658191
1300.	0.7673667	0.7630181	0.7636207	0.758881	0.7641659
1310.	0.7659597	0.7625696	0.7615014	0.7575179	0.7623192
1320.	0.7646847	0.7606878	0.7599801	0.7565683	0.7613519
1330.	0.7623192	0.7591226	0.7593119	0.755091	0.7599801
1340.	0.7602087	0.7576717	0.7580368	0.7523122	0.7578433
1350.	0.7584588	0.7562823	0.7564275	0.7510635	0.7553723
1360.	0.7571398	0.7545852	0.7552844	0.7494015	0.7540885
1370.	0.7555569	0.7526331	0.754150	0.7477043	0.7523385
1380.	0.754361	0.7507073	0.7520132	0.7460864	0.7499555
1390.	0.7519692	0.7492388	0.750316	0.7445651	0.7479242
1400.	0.7507205	0.7480605	0.7487068	0.7424634	0.7460864
1410.	0.7491729	0.7464513	0.7459896	0.7401243	0.7453389
1420.	0.7479241	0.7446839	0.7442749	0.7383128	0.743844
1430.	0.7459984	0.743233	0.7429206	0.7362903	0.741971
1440.	0.7444419	0.7420458	0.7407399	0.7340128	0.7403266
1450.	0.7436417	0.7399091	0.7393768	0.7330807	0.7399748
1460.	0.7422611	0.7390473	0.7370378	0.7317705	0.738093
1470.	0.739966	0.7371743	0.7355956	0.7306009	0.7359122
1480.	0.7389811	0.7358641	0.7339249	0.7292907	0.7351823
1490.	0.7382952	0.7338943	0.7316649	0.7274528	0.7342678
1500.	0.7369762	0.7329711	0.7294402	0.7256941	0.7329839
1510.	0.7351999	0.7326984	0.7277166	0.723865	0.7319199
1520.	0.7340919	0.7307463	0.7264679	0.7227395	0.730768
1530.	0.7318671	0.7288381	0.7247444	0.7209104	0.7289653
1540.	0.7306536	0.7280116	0.7223262	0.7191869	0.7274968
1550.	0.7287454	0.7265782	0.7215699	0.716751	0.7252808
1560.	0.7268021	0.7248899	0.7202421	0.7151594	0.724463
1570.	0.7250697	0.7240722	0.7181668	0.7131809	0.7228186
1580.	0.7234605	0.7222695	0.7159508	0.7110265	0.7224229
1590.	0.7214644	0.720458	0.713770	0.7094612	0.7209808

1600.	0.7200398	0.7192622	0.7121432	0.7078344	0.720119
1610.	0.718668	0.7174683	0.7107099	0.7059878	0.7195298
1620.	0.7168829	0.7157975	0.7093909	0.7042466	0.7182108
1630.	0.7155023	0.7143731	0.7081686	0.7040532	0.7170148
1640.	0.7134095	0.7132826	0.7065857	0.7019515	0.715177
1650.	0.7117739	0.7119197	0.7052755	0.6996916	0.714368
1660.	0.7101207	0.7106798	0.7038069	0.6980824	0.7131545
1670.	0.7085466	0.7085518	0.7025847	0.6966138	0.7117651
1680.	0.706234	0.7061776	0.7018548	0.6953475	0.7107539
1690.	0.7046247	0.7042518	0.7002192	0.6937032	0.709426
1700.	0.7031034	0.7024052	0.6996213	0.692006	0.7071573
1710.	0.7017493	0.700585	0.698355	0.6907925	0.7058119
1720.	0.7008523	0.6987823	0.6965963	0.6897373	0.7057415
1730.	0.6994014	0.6968918	0.6952685	0.689535	0.7040796
1740.	0.6983461	0.6951243	0.6930436	0.6885062	0.7023824
1750.	0.6968073	0.692539	0.6909947	0.6873806	0.7004918
1760.	0.6954267	0.690666	0.6893504	0.6869937	0.699164
1770.	0.6942923	0.6892943	0.6888667	0.684980	0.6971591
1780.	0.6923929	0.688028	0.6873015	0.6829311	0.695497
1790.	0.6919972	0.6873158	0.6861231	0.6820693	0.6935361
1800.	0.6906606	0.6852405	0.6845051	0.6812691	0.6925073
1810.	0.6895438	0.6835961	0.6826497	0.6800556	0.6910563
1820.	0.6882424	0.681477	0.6807767	0.6789652	0.6891569
1830.	0.6866771	0.6801316	0.679484	0.6776462	0.6876884
1840.	0.6855691	0.6784257	0.6782177	0.6761161	0.6872839
1850.	0.6840918	0.6776958	0.6779276	0.6747003	0.686211
1860.	0.682465	0.6756909	0.6766173	0.6736451	0.6842325
1870.	0.6804513	0.6740905	0.6751576	0.6717017	0.6829662
1880.	0.6784199	0.6717427	0.6745157	0.670796	0.6814449
1890.	0.6774614	0.6703181	0.6736891	0.6699518	0.6803106
1900.	0.6768283	0.6692014	0.6711917	0.6693978	0.6787277
1910.	0.6756852	0.6672404	0.6695033	0.6683778	0.6777781
1920.	0.6749026	0.6649981	0.668835	0.666918	0.6761776
1930.	0.6741023	0.6628525	0.6666894	0.6657221	0.6747531
1940.	0.6722117	0.661472	0.6661882	0.6640074	0.6722733
1950.	0.6701452	0.6600651	0.6643591	0.663084	0.6707608
1960.	0.6688174	0.6587988	0.6630225	0.6622663	0.6695385
1970.	0.6672082	0.6583767	0.6612726	0.660657	0.6682459
1980.	0.6659595	0.6569874	0.6596985	0.658740	0.667173
1990.	0.6654407	0.6548418	0.6580629	0.6566823	0.6654055
2000.	0.6643503	0.6541735	0.6563394	0.6544927	0.6635062
2010.	0.6629521	0.6533117	0.654440	0.6537893	0.6627675
2020.	0.6619233	0.6513156	0.6529979	0.6517931	0.6613429
2030.	0.6608592	0.6493107	0.6515733	0.6502279	0.6597601
2040.	0.6607449	0.647939	0.6505796	0.6483988	0.6586785
2050.	0.658995	0.646998	0.6490408	0.6478009	0.6568055
2060.	0.6573858	0.6449932	0.6470094	0.6471326	0.6556007
2070.	0.6564801	0.6423024	0.6454794	0.6458575	0.6543257
2080.	0.6557062	0.6411768	0.6446352	0.6452156	0.6526461
2090.	0.6540091	0.6403327	0.6425511	0.6437294	0.6508874
2100.	0.6520833	0.6392687	0.6409858	0.6424808	0.6487945
2110.	0.6506324	0.6379672	0.6385764	0.6412497	0.6483197
2120.	0.6497442	0.6366482	0.6365275	0.6403703	0.6471238
2130.	0.6489264	0.6345378	0.6349711	0.6383566	0.6455585
2140.	0.6470798	0.6332892	0.6333443	0.6366243	0.6446176
2150.	0.6454266	0.6325945	0.6311547	0.6359648	0.6436151

2160.	0.6441427	0.632146	0.6295719	0.6347513	0.6423489
2170.	0.6427445	0.6307567	0.6284902	0.6338367	0.6411617
2180.	0.641619	0.6294729	0.6275141	0.6319901	0.6393766
2190.	0.6393854	0.6280747	0.6267052	0.6312075	0.6384181
2200.	0.6385061	0.6264479	0.6252366	0.6294135	0.6369233
2210.	0.6362549	0.6250849	0.6249728	0.6273646	0.6353756
2220.	0.6353052	0.6231943	0.6237417	0.6272151	0.6349711
2230.	0.6341181	0.6222622	0.6225458	0.6269074	0.6329573
2240.	0.6339598	0.6206003	0.6219566	0.6252894	0.6320868
2250.	0.6325352	0.6200639	0.6200924	0.6241198	0.6308733
2260.	0.6314272	0.6191142	0.6188525	0.622493	0.6295982
2270.	0.6307238	0.6178743	0.6167597	0.6215258	0.6282528
2280.	0.6292904	0.6163795	0.6146756	0.620277	0.6263358
2290.	0.6278395	0.6162036	0.6131367	0.6188965	0.6248145
2300.	0.6271976	0.6145505	0.6127674	0.617551	0.6227129
2310.	0.6256323	0.6132314	0.6117385	0.6164255	0.6220797
2320.	0.6245683	0.6123785	0.610182	0.6147547	0.6208135
2330.	0.6230822	0.6106462	0.6089774	0.6128817	0.6195296
2340.	0.6213586	0.6095734	0.6078166	0.6116066	0.6174015
2350.	0.6196879	0.6087996	0.6069021	0.6107097	0.6158539
2360.	0.6181051	0.6074806	0.6059787	0.6100062	0.6147899
2370.	0.6168123	0.6059065	0.6044574	0.6084585	0.6142095
2380.	0.6155813	0.604737	0.603156	0.6078166	0.6129784
2390.	0.6138489	0.603673	0.6018721	0.6069724	0.6114044
2400.	0.6132334	0.6025211	0.6000783	0.606014	0.6090565
2410.	0.6120023	0.6008943	0.5983635	0.6045718	0.6077638
2420.	0.6111669	0.5991444	0.5979326	0.6041321	0.6066119
2430.	0.6098039	0.598142	0.5965872	0.6024438	0.6054335
2440.	0.6086256	0.5968406	0.596385	0.6015204	0.6044927
2450.	0.6073769	0.5960491	0.5938612	0.6005532	0.603200
2460.	0.6061458	0.5954248	0.592639	0.5988208	0.6020041
2470.	0.604818	0.5938596	0.591188	0.5976161	0.6008785
2480.	0.6037276	0.5928659	0.5897371	0.5964466	0.6008609
2490.	0.6025141	0.5915205	0.5890072	0.5949429	0.5997881
2500.	0.6017578	0.5899729	0.5876442	0.5934567	0.5982316
2510.	0.6007114	0.5885748	0.5870023	0.591953	0.5967456
2520.	0.5995155	0.5879328	0.5849534	0.590423	0.5957255
2530.	0.5985306	0.5868512	0.584628	0.588717	0.5940195
2540.	0.5967895	0.5857081	0.5835816	0.5878729	0.5925686
2550.	0.5947846	0.5848376	0.5816822	0.5867385	0.591443
2560.	0.5934128	0.5833955	0.5802488	0.5856481	0.5901768
2570.	0.592252	0.5826656	0.5790178	0.5841004	0.5890688
2580.	0.5906692	0.5816984	0.5772854	0.5834761	0.5880751
2590.	0.589016	0.5801243	0.5755443	0.5822538	0.5865538
2600.	0.5882685	0.5793065	0.5744715	0.580838	0.5852612
2610.	0.5867736	0.5786646	0.5739527	0.5793519	0.5840124
2620.	0.5865098	0.5776182	0.5728447	0.578068	0.5825351
2630.	0.5842235	0.5762025	0.5710948	0.5760543	0.5816294
2640.	0.5831067	0.5758156	0.5701978	0.5749991	0.5799586
2650.	0.5808028	0.5738458	0.5688348	0.5738295	0.5780153
2660.	0.5792375	0.5724037	0.5676213	0.5727215	0.5766611
2670.	0.5777866	0.5714101	0.5663902	0.5720444	0.5742604
2680.	0.5769512	0.5696954	0.5657131	0.5712178	0.5737856
2690.	0.5759488	0.5692645	0.5643413	0.5700835	0.5726512
2700.	0.5753772	0.5674267	0.5633301	0.5681841	0.5708045
2710.	0.5737944	0.5657296	0.5631102	0.5668474	0.5699692

2720.	0.5714904	0.5637862	0.5621429	0.5646403	0.568527
2730.	0.5703297	0.5617549	0.5608415	0.5628288	0.5672783
2740.	0.5690986	0.5601193	0.5594609	0.5618967	0.5662847
2750.	0.5689051	0.5587476	0.5586519	0.5610525	0.5644908
2760.	0.5683247	0.5573406	0.5580539	0.5597335	0.5636642
2770.	0.5671113	0.5565053	0.5564007	0.5588278	0.5629695
2780.	0.5664253	0.555283	0.5548091	0.5576758	0.5610525
2790.	0.5648689	0.5543861	0.5533581	0.5566733	0.5601996
2800.	0.5636994	0.5527241	0.5518808	0.5550202	0.5598126
2810.	0.5630134	0.5515634	0.550342	0.5540265	0.5582914
2820.	0.5613779	0.5508863	0.5489702	0.5527778	0.5570691
2830.	0.5606128	0.550174	0.5485744	0.5519336	0.5558292
2840.	0.5594785	0.548899	0.5475017	0.5511774	0.5542111
2850.	0.5577989	0.5487407	0.5466751	0.5504827	0.5529009
2860.	0.5566205	0.5477294	0.5451274	0.5484778	0.552215
2870.	0.5555653	0.5464193	0.5435182	0.5479589	0.5515555
2880.	0.5539297	0.5450299	0.5425773	0.5462442	0.5498056
2890.	0.552171	0.5436845	0.5417947	0.5454615	0.5493835
2900.	0.5510015	0.5424886	0.5403877	0.5442393	0.5485217
2910.	0.549700	0.5411784	0.5391214	0.5433423	0.5472466
2920.	0.5487855	0.5404221	0.5369055	0.5426124	0.5459101
2930.	0.547273	0.5393054	0.5350412	0.5413989	0.5443448
2940.	0.5467806	0.5383733	0.5346015	0.540502	0.5436853
2950.	0.5455143	0.5376347	0.5332737	0.5392533	0.5423399
2960.	0.5434566	0.5366849	0.5319986	0.5385938	0.541100
2970.	0.5422695	0.5358232	0.5308906	0.5373099	0.5393852
2980.	0.5410823	0.5348647	0.531005	0.5360965	0.537820
2990.	0.5397106	0.5336072	0.5291671	0.5353754	0.5373891
3000.	0.5381101	0.5330621	0.528147	0.5343025	0.5357359
3010.	0.5374154	0.5308725	0.5268368	0.532878	0.5357095
3020.	0.5359821	0.5299404	0.5254387	0.5326582	0.5344608
3030.	0.5350324	0.5291402	0.5247616	0.5316205	0.5335727
3040.	0.5343201	0.5276805	0.5239525	0.5304773	0.532623
3050.	0.533344	0.5266429	0.5226863	0.5292902	0.5319195
3060.	0.5319107	0.5256844	0.5221323	0.5287714	0.5305389
3070.	0.5312424	0.5242863	0.521376	0.5269511	0.5295892
3080.	0.5299321	0.5228441	0.5201801	0.525465	0.5278921
3090.	0.5287714	0.5214812	0.5188171	0.5243043	0.5268544
3100.	0.5275139	0.5203117	0.5171551	0.5226863	0.5261509
3110.	0.5260454	0.5195642	0.516522	0.5211914	0.5236272
3120.	0.5251485	0.5186849	0.5158449	0.5207956	0.5227391
3130.	0.5247527	0.5181661	0.5156251	0.5198811	0.5220268
3140.	0.5240492	0.5166273	0.5140246	0.5184126	0.5211562
3150.	0.5228621	0.5162315	0.5127671	0.5167506	0.5204879
3160.	0.5217366	0.5144992	0.5114217	0.5152557	0.519969
3170.	0.5207077	0.5135759	0.5108765	0.5141829	0.5187556
3180.	0.5191424	0.5121514	0.5100588	0.5129342	0.5174013
3190.	0.5181839	0.5109379	0.509197	0.5115976	0.5163022
3200.	0.5172782	0.5091001	0.5087749	0.5099093	0.5150007
3210.	0.5163725	0.5083175	0.5075262	0.5093025	0.5135674
3220.	0.5154316	0.5074469	0.5068403	0.5080978	0.5119054
3230.	0.5135497	0.5069545	0.505363	0.5073416	0.5116064
3240.	0.5119317	0.5055563	0.5043253	0.5063831	0.5109293
3250.	0.5101994	0.5047297	0.5036834	0.5055037	0.5102434
3260.	0.5089507	0.5037977	0.5025402	0.504255	0.5094696
3270.	0.5072008	0.5025314	0.5013883	0.5039648	0.5080186

3280.	0.5064886	0.5017312	0.5008871	0.5031822	0.506216
3290.	0.5052662	0.5007024	0.4996912	0.5017489	0.5053454
3300.	0.5037274	0.4992603	0.4988909	0.5018192	0.5044397
3310.	0.5028216	0.4974664	0.4972993	0.5003771	0.5044661
3320.	0.501995	0.4966135	0.4970795	0.4996912	0.5025402
3330.	0.5007112	0.4955758	0.4964727	0.4991196	0.5017313
3340.	0.4993482	0.493993	0.4953999	0.4982578	0.500632
3350.	0.4979412	0.4929202	0.4944678	0.4982402	0.499357
3360.	0.497141	0.4920233	0.493351	0.4965518	0.4981171
3370.	0.4962968	0.4914693	0.4920847	0.4952944	0.4972641
3380.	0.4951888	0.4906252	0.4918297	0.4939314	0.4964727
3390.	0.4937027	0.4896491	0.4913021	0.4928498	0.4956373
3400.	0.4928234	0.4882949	0.4895786	0.4916011	0.4947668
3410.	0.4916802	0.4879344	0.4882595	0.4905722	0.4937555
3420.	0.4908976	0.4867121	0.4878638	0.4901941	0.4927442
3430.	0.4894291	0.4854898	0.4861755	0.4888663	0.4914868
3440.	0.4888311	0.4843379	0.4848564	0.4875649	0.4906514
3450.	0.4878462	0.4833266	0.4833088	0.4865536	0.4890422
3460.	0.4873538	0.4823066	0.482148	0.4862546	0.4876704
3470.	0.4860348	0.4813833	0.4812159	0.4847245	0.4871516
3480.	0.4843376	0.480161	0.4803453	0.4838012	0.4864129
3490.	0.4828955	0.479141	0.4790439	0.4828339	0.4855335
3500.	0.4817171	0.4781297	0.4783228	0.4817171	0.4844519
3510.	0.4806619	0.4768987	0.4768983	0.4811104	0.4831944
3520.	0.4801607	0.4758259	0.476230	0.4798881	0.4819634
3530.	0.478824	0.4746827	0.4751571	0.4789736	0.4811016
3540.	0.4777161	0.4747179	0.4741283	0.4779447	0.4794572
3550.	0.4762299	0.4737594	0.4729148	0.4773292	0.4788065
3560.	0.4753858	0.4728361	0.4716397	0.476485	0.4779447
3570.	0.4752275	0.4719656	0.4705933	0.4755353	0.4762739
3580.	0.4744185	0.4711214	0.4699514	0.4753155	0.4747878
3590.	0.4735391	0.4705674	0.4683685	0.4746911	0.4744713
3600.	0.4720706	0.4695914	0.4672342	0.4731083	0.4730995
3610.	0.4712352	0.4685977	0.4669879	0.4719651	0.4715518
3620.	0.4701448	0.4680701	0.4661438	0.4708132	0.4698371
3630.	0.4701096	0.466628	0.4652205	0.4705318	0.469371
3640.	0.469626	0.4658014	0.4642619	0.4694501	0.4686675
3650.	0.4681311	0.4646935	0.4625032	0.4686235	0.4680256
3660.	0.4676474	0.4632777	0.4619316	0.466944	0.4667593
3670.	0.4665658	0.4618796	0.4602697	0.4663284	0.4656953
3680.	0.4649742	0.4598659	0.4595222	0.4652996	0.4648159
3690.	0.4640509	0.4590569	0.4591969	0.4640421	0.4637167
3700.	0.4633034	0.4573773	0.4588187	0.4628638	0.4629957
3710.	0.4616239	0.4551966	0.4573766	0.4607445	0.4618701
3720.	0.4603048	0.4544228	0.4566379	0.459786	0.4617118
3730.	0.4589946	0.4541765	0.4564357	0.4588803	0.4612897
3740.	0.4583966	0.4531741	0.4566204	0.4586605	0.4605687
3750.	0.4578954	0.4522948	0.4554508	0.4577723	0.4589506
3760.	0.4572887	0.4516177	0.4539647	0.4565764	0.4576756
3770.	0.4568666	0.4503602	0.4527864	0.4552486	0.4563126
3780.	0.4557586	0.4498062	0.4520653	0.4545802	0.4558729
3790.	0.4542549	0.449815	0.4507111	0.4543076	0.4551255
3800.	0.4533931	0.4484521	0.450034	0.4537888	0.454422
3810.	0.4530326	0.4476343	0.4490667	0.4534635	0.4538416
3820.	0.4517223	0.4470803	0.4473959	0.4528128	0.4534459
3830.	0.4508518	0.4462009	0.4463407	0.4519774	0.452206

3840.	0.4503417	0.4454887	0.4456988	0.4507023	0.4502802
3850.	0.4497966	0.4438531	0.4451624	0.4502714	0.4502626
3860.	0.4488908	0.4424725	0.443984	0.4491898	0.4492426
3870.	0.4481697	0.4422351	0.443430	0.4487326	0.4485567
3880.	0.4473783	0.4412151	0.4422077	0.448117	0.4477037
3890.	0.4460505	0.439984	0.4413987	0.4474487	0.4466661
3900.	0.4454086	0.4386474	0.4406953	0.4463495	0.4460241
3910.	0.4448898	0.4377417	0.4401061	0.4453118	0.4443006
3920.	0.4430343	0.4373812	0.4390333	0.4450041	0.4428672
3930.	0.442366	0.4367481	0.4380308	0.4441599	0.4417065
3940.	0.4414339	0.4353499	0.4371426	0.4427969	0.4406073
3950.	0.4403962	0.4343738	0.4365095	0.4422429	0.4397807
3960.	0.4391564	0.4335824	0.4360698	0.4411261	0.4394993
3970.	0.4381099	0.4329845	0.4347068	0.4402643	0.4387343
3980.	0.4369052	0.4321227	0.433889	0.4398511	0.436703
3990.	0.4357708	0.4303816	0.4326491	0.4389277	0.4362457
4000.	0.4350849	0.4298277	0.4319896	0.437723	0.435296
4010.	0.4344166	0.4288868	0.431251	0.4367206	0.4344342
4020.	0.433889	0.4273303	0.4296681	0.4360698	0.4330624
4030.	0.432825	0.4268467	0.4288415	0.4352169	0.4324821
4040.	0.4319457	0.4266884	0.428437	0.4344079	0.4315851
4050.	0.430908	0.4255365	0.4268982	0.4337132	0.4305475
4060.	0.4301957	0.4255277	0.4258517	0.4332735	0.4296418
4070.	0.4300374	0.4244813	0.4242777	0.4325964	0.4281293
4080.	0.4289294	0.4237426	0.4232313	0.4311718	0.4262299
4090.	0.4277951	0.4228985	0.4220353	0.4309256	0.4247789
4100.	0.4277072	0.4222389	0.4210065	0.4294131	0.4239435
4110.	0.4269597	0.4209815	0.4202327	0.4287624	0.4232137
4120.	0.4257462	0.4196449	0.4194236	0.4281908	0.4225542
4130.	0.4254208	0.4182819	0.4185267	0.4275313	0.4215693
4140.	0.424647	0.4179478	0.4176122	0.4268454	0.4211736
4150.	0.4226069	0.4172004	0.4170406	0.4262738	0.4198985
4160.	0.4218507	0.4165936	0.4162668	0.4252714	0.4188169
4170.	0.4212703	0.4154768	0.4147455	0.4239523	0.4177353
4180.	0.4193269	0.4145887	0.4137254	0.4226421	0.4161085
4190.	0.4185003	0.4134016	0.413277	0.4210153	0.4150005
4200.	0.417533	0.4118803	0.4131363	0.4195555	0.414851
4210.	0.4167328	0.4103591	0.4119579	0.4188257	0.4133561
4220.	0.4156336	0.4095677	0.411272	0.4179639	0.4127757
4230.	0.4148158	0.4086444	0.4103135	0.4168999	0.4117205
4240.	0.4133561	0.4073341	0.4096452	0.4155633	0.4106565
4250.	0.4119228	0.4065779	0.409100	0.4145432	0.4097068
4260.	0.4103751	0.4055843	0.4081767	0.4133825	0.4080712
4270.	0.4090824	0.4047753	0.4075963	0.4133913	0.4076227
4280.	0.4086164	0.4038608	0.406207	0.4128725	0.4064972
4290.	0.4081767	0.4031749	0.4051341	0.4119579	0.4058025
4300.	0.4079832	0.4016536	0.4040701	0.4107972	0.4052748
4310.	0.4073413	0.4006248	0.4037448	0.4095749	0.404202
4320.	0.4062949	0.4003698	0.4025928	0.4090473	0.4031116
4330.	0.4054683	0.399763	0.4011155	0.4080448	0.4020916
4340.	0.4045889	0.3989716	0.3999372	0.4071391	0.4004296
4350.	0.4035601	0.3981802	0.3998668	0.4063213	0.3981696
4360.	0.4030412	0.3976526	0.3991018	0.4056266	0.3974134
4370.	0.4024873	0.3973097	0.3985214	0.4046417	0.3966044
4380.	0.4006582	0.3971954	0.3977388	0.4038679	0.3955668
4390.	0.3989259	0.3965535	0.397176	0.403437	0.3950128

4400.	0.3989611	0.3949267	0.3970353	0.4025224	0.3946962
4410.	0.3983719	0.3938187	0.396112	0.4016431	0.3939487
4420.	0.3975893	0.392702	0.395259	0.4008165	0.3933332
4430.	0.397088	0.392148	0.3940894	0.4002449	0.3928935
4440.	0.3958218	0.3910576	0.3936762	0.3988995	0.3920054
4450.	0.3951095	0.3906003	0.3929287	0.3987588	0.3914426
4460.	0.3944939	0.3899672	0.3920845	0.3979762	0.390915
4470.	0.3933508	0.3896419	0.3910205	0.3975893	0.390273
4480.	0.3930518	0.3886394	0.3901939	0.3967627	0.3889804
4490.	0.3924626	0.3876985	0.3894201	0.3962439	0.3882417
4500.	0.3914074	0.3862388	0.3888309	0.395004	0.3875998
4510.	0.3905368	0.3849814	0.3879516	0.3943972	0.3868611
4520.	0.3896135	0.3844977	0.3867556	0.3937025	0.3860521
4530.	0.3888309	0.3844977	0.3858059	0.392788	0.3853662
4540.	0.3880834	0.3833546	0.3853751	0.3915921	0.3846188
4550.	0.3874855	0.3831171	0.3848034	0.3910293	0.3838186
4560.	0.3871689	0.3820092	0.3843462	0.3906687	0.383247
4570.	0.3866061	0.3814728	0.3836515	0.3907655	0.3821038
4580.	0.3861928	0.3805231	0.3832382	0.3901763	0.3813124
4590.	0.3856564	0.3797493	0.3826315	0.3892442	0.3811102
4600.	0.384865	0.379081	0.3817873	0.3886638	0.3799846
4610.	0.3837834	0.3784567	0.3811014	0.3880659	0.3786744
4620.	0.3831327	0.3775246	0.3812333	0.3864566	0.3776015
4630.	0.3825347	0.3767859	0.3802748	0.3859202	0.3767222
4640.	0.3821654	0.3757659	0.380011	0.3853926	0.3764584
4650.	0.381286	0.3755197	0.3788678	0.3837043	0.375922
4660.	0.3800813	0.3750888	0.3779797	0.3828073	0.3749635
4670.	0.3791932	0.3746843	0.3772234	0.3818136	0.3741896
4680.	0.378604	0.373462	0.3761154	0.3812772	0.3737324
4690.	0.3778301	0.372398	0.3752449	0.3800374	0.3728794
4700.	0.3776807	0.3717297	0.374216	0.3793691	0.371789
4710.	0.3767837	0.3707097	0.3735125	0.3790877	0.3700567
4720.	0.376687	0.3691972	0.3735653	0.3783138	0.3691158
4730.	0.3764848	0.3685729	0.3732048	0.3778038	0.3688432
4740.	0.3760715	0.3679046	0.3723606	0.3774345	0.367744
4750.	0.3749283	0.3674649	0.3724925	0.3767574	0.3670669
4760.	0.3738643	0.365548	0.3713054	0.3765463	0.3662051
4770.	0.3728178	0.3634287	0.3707162	0.3761418	0.3649476
4780.	0.372642	0.362866	0.3701974	0.3747261	0.3637957
4790.	0.3712086	0.3617316	0.3700215	0.3739258	0.3629163
4800.	0.370637	0.3605797	0.3697841	0.3731696	0.3627844
4810.	0.3701446	0.3597267	0.3694235	0.3724397	0.3616149
4820.	0.3696258	0.3587946	0.3688432	0.3715604	0.3610521
4830.	0.3685354	0.3575899	0.3680606	0.3706546	0.3612016
4840.	0.367911	0.3571151	0.3668646	0.3705843	0.3602167
4850.	0.3674626	0.3561039	0.3654928	0.3701182	0.3594781
4860.	0.3669262	0.3556378	0.3657039	0.3696786	0.3586427
4870.	0.3659501	0.3546441	0.3646398	0.3687025	0.3578249
4880.	0.3655807	0.3540286	0.3640859	0.3677792	0.3572445
4890.	0.3648069	0.3533603	0.3631186	0.3675681	0.3568664
4900.	0.3643936	0.3522963	0.3623184	0.3669086	0.3553011
4910.	0.363611	0.3512411	0.3610345	0.3664162	0.3547119
4920.	0.3624502	0.3506783	0.3597507	0.3653433	0.3545097
4930.	0.361351	0.3501332	0.3592055	0.3647805	0.3534281
4940.	0.3602519	0.349456	0.3578161	0.364420	0.3527686
4950.	0.3593725	0.3487878	0.3576138	0.3637605	0.3523729

4960.	0.3582381	0.3482601	0.3564882	0.3631186	0.3520299
4970.	0.3576666	0.3482953	0.3563387	0.3624063	0.351643
4980.	0.3569894	0.3470115	0.3557144	0.3616325	0.3506405
4990.	0.356541	0.3459651	0.3541316	0.3605596	0.3502272
5000.	0.3559958	0.3454463	0.3536655	0.3599441	0.3487499
5010.	0.3547999	0.3445317	0.3532698	0.3592494	0.3481607
5020.	0.3536831	0.3432128	0.3528125	0.3585635	0.3470264
5030.	0.3525751	0.3423949	0.3518452	0.3578161	0.3462174
5040.	0.3519156	0.3412782	0.3508691	0.3568927	0.3451885
5050.	0.3504734	0.3403813	0.3500953	0.3564003	0.3446082
5060.	0.3498843	0.3395635	0.3494534	0.3553539	0.3435265
5070.	0.349128	0.3389216	0.3488906	0.3542195	0.342867
5080.	0.3480113	0.3382972	0.3481607	0.3537534	0.3415568
5090.	0.3472638	0.3378839	0.3470879	0.3533929	0.3413985
5100.	0.3465075	0.3369782	0.3465427	0.3527862	0.3401938
5110.	0.3456634	0.3360461	0.3457953	0.3517573	0.3390067
5120.	0.3448631	0.3352635	0.3450654	0.3515111	0.3378899
5130.	0.3446433	0.334947	0.343931	0.3507724	0.3371249
5140.	0.3436233	0.3340324	0.343122	0.3501569	0.3361224
5150.	0.3421899	0.3332058	0.3424889	0.3495325	0.3357531
5160.	0.3410555	0.3325903	0.3417415	0.3487411	0.335243
5170.	0.3400619	0.3320979	0.3408445	0.348108	0.3350408
5180.	0.3391562	0.3312362	0.3396574	0.3479233	0.3345835
5190.	0.3385494	0.3299787	0.3395431	0.3472374	0.3331766
5200.	0.3376085	0.3295126	0.3389539	0.3467538	0.3320334
5210.	0.3368786	0.3292136	0.3376613	0.3465164	0.3316025
5220.	0.3368434	0.3285893	0.3367291	0.3458041	0.3315058
5230.	0.335885	0.3282376	0.3362015	0.3456898	0.3307231
5240.	0.3352254	0.3275165	0.3354629	0.3447137	0.3304593
5250.	0.333880	0.3272351	0.334689	0.3442212	0.329879
5260.	0.333414	0.326646	0.3335899	0.3432891	0.3296503
5270.	0.3326313	0.3260656	0.3330622	0.3436233	0.3291843
5280.	0.3319454	0.3255292	0.3325258	0.3423834	0.328727
5290.	0.3310749	0.3254061	0.3322268	0.3419085	0.3277949
5300.	0.3304505	0.3254061	0.3314618	0.3407918	0.3272849
5310.	0.3299141	0.3245707	0.3305912	0.3402378	0.3273728
5320.	0.3286303	0.3237969	0.3300988	0.3397629	0.3272761
5330.	0.3275223	0.3233836	0.330090	0.3394815	0.3265638
5340.	0.3260274	0.3230143	0.3300988	0.3386285	0.3256844
5350.	0.3255789	0.3218096	0.3289644	0.3378459	0.3249194
5360.	0.3248754	0.3206929	0.3284017	0.3372304	0.3242159
5370.	0.3244006	0.3200949	0.3279268	0.3362367	0.3237059
5380.	0.3233805	0.3193475	0.3270035	0.3354541	0.3228529
5390.	0.3231079	0.3193211	0.3264143	0.3349792	0.3219296
5400.	0.3220791	0.318855	0.3260186	0.3347682	0.3212437
5410.	0.3216834	0.3185385	0.3256317	0.3343461	0.3209096
5420.	0.3213404	0.3181252	0.3249106	0.3333964	0.3204787
5430.	0.321147	0.3176503	0.324084	0.333115	0.3194674
5440.	0.3205138	0.3169909	0.3233981	0.3326401	0.3190453
5450.	0.319696	0.3161643	0.3229409	0.3314794	0.318931
5460.	0.3187991	0.3154608	0.3217713	0.3308638	0.3184298
5470.	0.3179989	0.3148453	0.3211822	0.3308375	0.3180692
5480.	0.317014	0.3150035	0.3203907	0.3298086	0.3174009
5490.	0.3159939	0.3135966	0.3193003	0.3288589	0.3168206
5500.	0.3156686	0.3129635	0.3182275	0.3273904	0.3161083
5510.	0.3152377	0.3118643	0.3177527	0.3265462	0.3152553

5520.	0.3137868	0.3107124	0.3172514	0.3258603	0.3144815
5530.	0.3130569	0.3104749	0.3160643	0.3253679	0.3139978
5540.	0.312714	0.3102112	0.3143671	0.3245501	0.3132679
5550.	0.3119577	0.3096835	0.3138219	0.3243742	0.3119665
5560.	0.3113861	0.3092263	0.3125908	0.3237499	0.3115532
5570.	0.3111135	0.3087514	0.3122391	0.3232222	0.3107002
5580.	0.3106562	0.3081886	0.3115093	0.3229145	0.3101111
5590.	0.3103924	0.3079161	0.310199	0.3224572	0.3096626
5600.	0.3094428	0.3071686	0.309689	0.3222462	0.3088624
5610.	0.3084667	0.3054715	0.3087305	0.3213844	0.3081413
5620.	0.3072883	0.3051462	0.3076753	0.3203644	0.308027
5630.	0.3061891	0.3045394	0.3070421	0.3190805	0.3077192
5640.	0.3061188	0.3039678	0.3061628	0.317867	0.306963
5650.	0.3061452	0.3037216	0.3054857	0.3173394	0.3061628
5660.	0.3058638	0.3029566	0.3048349	0.3163721	0.3056176
5670.	0.3055824	0.3020245	0.303938	0.3153432	0.3043425
5680.	0.3053801	0.3013474	0.3032521	0.3142264	0.3032609
5690.	0.3042985	0.3010045	0.3024431	0.3139538	0.3025047
5700.	0.3039204	0.3003098	0.302021	0.3136461	0.3021441
5710.	0.3035247	0.2999581	0.3005261	0.3128019	0.301168
5720.	0.3031114	0.2993777	0.3005789	0.311905	0.3005789
5730.	0.3023727	0.2985687	0.2996379	0.3108937	0.2996819
5740.	0.302065	0.298129	0.2992159	0.3100935	0.2995324
5750.	0.3013615	0.2972585	0.2984684	0.309689	0.2989872
5760.	0.3010625	0.2965638	0.2978001	0.3087481	0.2984596
5770.	0.3002799	0.2957108	0.297677	0.3079127	0.2980024
5780.	0.2993917	0.2955965	0.2970702	0.3067959	0.2978177
5790.	0.2990048	0.2951305	0.2961645	0.3063914	0.2975539
5800.	0.2982749	0.2944798	0.2954874	0.3056967	0.2969471
5810.	0.2983101	0.294172	0.2945729	0.3050812	0.2969295
5820.	0.2978353	0.2935828	0.2940365	0.3047646	0.2963755
5830.	0.2974044	0.2930289	0.2935528	0.3042458	0.2960238
5840.	0.2969647	0.2919385	0.2933857	0.3035335	0.2951884
5850.	0.2962348	0.2909976	0.2924888	0.3027861	0.2944586
5860.	0.2957952	0.2901622	0.2915831	0.3025574	0.2928405
5870.	0.2955929	0.2892829	0.291627	0.3024783	0.2921722
5880.	0.2950125	0.2881134	0.2908796	0.3019067	0.290906
5890.	0.2950213	0.2875506	0.2900178	0.3009042	0.2903432
5900.	0.2943618	0.2874538	0.2894374	0.3002799	0.290264
5910.	0.2939573	0.2873571	0.2887164	0.2996643	0.2896045
5920.	0.2934209	0.2868647	0.2885669	0.2992774	0.2885229
5930.	0.2928669	0.286425	0.2875204	0.2984684	0.2877667
5940.	0.2923305	0.2855984	0.2867027	0.2978265	0.2867818
5950.	0.2918469	0.2852291	0.2859376	0.2968416	0.2861926
5960.	0.2914599	0.2844905	0.2857002	0.295760	0.2855859
5970.	0.290818	0.2840068	0.2849351	0.294696	0.2851638
5980.	0.2895342	0.283743	0.2842405	0.2943355	0.2844515
5990.	0.2898771	0.2828285	0.2836337	0.2938694	0.2836073
6000.	0.2895078	0.2819668	0.2827367	0.2934473	0.2831765
6010.	0.2887867	0.2814304	0.2822443	0.2925679	0.2826928
6020.	0.2882239	0.2814655	0.2814177	0.2917501	0.2827455
6030.	0.2874325	0.2806214	0.2802042	0.2910291	0.2821828
6040.	0.2869225	0.2801113	0.2798613	0.290651	0.2812682
6050.	0.2862278	0.2793287	0.2787093	0.2901497	0.2809253
6060.	0.2857793	0.279188	0.2784455	0.2894023	0.2808461
6070.	0.2850055	0.2795662	0.2779091	0.2889186	0.2803977

6080.	0.2841261	0.278678	0.2775046	0.2882415	0.2801075
6090.	0.2833347	0.2776844	0.2768539	0.2880129	0.2794919
6100.	0.2824202	0.2770952	0.2756843	0.2876172	0.2786126
6110.	0.2819277	0.2770337	0.2746291	0.2867554	0.2784543
6120.	0.2817343	0.2761543	0.2741455	0.2859552	0.2773024
6130.	0.2813034	0.2749672	0.2736091	0.2849351	0.2762207
6140.	0.2803361	0.2740088	0.2725187	0.2845306	0.275825
6150.	0.2794568	0.2739912	0.2722461	0.2838008	0.275570
6160.	0.2789379	0.2730415	0.2716129	0.2830445	0.2748753
6170.	0.2783136	0.2722325	0.2708303	0.2825873	0.2747874
6180.	0.2779794	0.271573	0.2702675	0.2824818	0.274550
6190.	0.2773111	0.2705793	0.2698806	0.2820685	0.2731958
6200.	0.2764845	0.2703243	0.2690452	0.2819805	0.2729759
6210.	0.2759041	0.2696912	0.2681483	0.281365	0.2720262
6220.	0.2749457	0.2683546	0.2674272	0.2809077	0.2722549
6230.	0.274251	0.2675544	0.2667677	0.2799844	0.2718679
6240.	0.2737585	0.2669828	0.2662313	0.2795095	0.2712436
6250.	0.2738904	0.266763	0.2653431	0.2790435	0.2705929
6260.	0.2734947	0.2663937	0.2650705	0.2785774	0.2697047
6270.	0.2728088	0.2659012	0.2646748	0.2776541	0.2688254
6280.	0.2726417	0.2652417	0.2642527	0.2771529	0.2684297
6290.	0.2718679	0.2644679	0.2642527	0.2765373	0.2676822
6300.	0.2715162	0.2640898	0.2635844	0.2758426	0.2669348
6310.	0.2707863	0.2635446	0.2631096	0.2753414	0.2663456
6320.	0.2706192	0.2628587	0.2626171	0.2742246	0.2660466
6330.	0.2703027	0.2622344	0.2627578	0.2734156	0.2655278
6340.	0.2696168	0.2616101	0.2617641	0.2734948	0.2650354
6350.	0.2691419	0.2612583	0.2612014	0.2735035	0.2645957
6360.	0.2689221	0.2604669	0.2605682	0.2728528	0.2637339
6370.	0.2680603	0.2598602	0.2596625	0.2721317	0.2630216
6380.	0.2675855	0.2591127	0.258704	0.2716041	0.2622478
6390.	0.2673744	0.2588314	0.2582467	0.2713227	0.2614476
6400.	0.2670491	0.2581631	0.2580445	0.2706896	0.2608848
6410.	0.2663808	0.2570287	0.2577895	0.2705841	0.2604891
6420.	0.2658443	0.256334	0.2571739	0.2697311	0.2601022
6430.	0.2650969	0.2560263	0.256743	0.2690804	0.2594163
6440.	0.2644022	0.2558768	0.2562418	0.2683681	0.258361
6450.	0.2640944	0.2557712	0.2563122	0.2678581	0.2571211
6460.	0.263901	0.2553228	0.2554504	0.267480	0.2568134
6470.	0.2633294	0.2543643	0.2550635	0.2672074	0.2558197
6480.	0.2626347	0.2536081	0.2544215	0.2667853	0.2551074
6490.	0.2620016	0.2534938	0.2537005	0.2658707	0.2544303
6500.	0.2617114	0.2529925	0.2531465	0.2656069	0.2536741
6510.	0.2613333	0.2528958	0.2522935	0.2647716	0.2535246
6520.	0.2609375	0.2525705	0.2520033	0.2644726	0.2529442
6530.	0.2602516	0.2513306	0.2515021	0.2632239	0.2527332
6540.	0.2598559	0.2508557	0.251335	0.2622478	0.2524782
6550.	0.2590733	0.2501259	0.2503413	0.2615004	0.2519418
6560.	0.2583259	0.2494576	0.2501743	0.2612629	0.2513878
6570.	0.2575081	0.2487277	0.2499544	0.2604715	0.2505348
6580.	0.2569365	0.247822	0.2496818	0.2595658	0.2498313
6590.	0.2562242	0.2472065	0.249207	0.2588887	0.2492685
6600.	0.255679	0.2471977	0.2488992	0.2581236	0.2487761
6610.	0.2552041	0.247057	0.2477033	0.2578334	0.2486178
6620.	0.2547117	0.2468723	0.2472284	0.2572091	0.2475889
6630.	0.2543072	0.2454654	0.2469119	0.2569981	0.2468327

6640.	0.2539203	0.244674	0.2467712	0.2566023	0.2464194
6650.	0.2532256	0.244076	0.2461204	0.2562946	0.2462875
6660.	0.2525309	0.2443047	0.246226	0.2560659	0.2457423
6670.	0.2517395	0.2442431	0.2457951	0.254826	0.2455137
6680.	0.2510272	0.2435308	0.2451883	0.2541226	0.2449685
6690.	0.2502446	0.2434517	0.2446431	0.2531113	0.2444672
6700.	0.2494796	0.2431615	0.2443881	0.2526101	0.2435791
6710.	0.2493125	0.2424493	0.2442034	0.2522056	0.2427965
6720.	0.2486354	0.2419305	0.2433505	0.2517659	0.2422513
6730.	0.2483364	0.2413765	0.2435439	0.2513878	0.2414071
6740.	0.248055	0.241183	0.2428492	0.2511328	0.2410641
6750.	0.2479231	0.2402685	0.2426997	0.2507898	0.2402815
6760.	0.2475274	0.2400223	0.2419523	0.2502886	0.2394197
6770.	0.2472724	0.2394771	0.2418204	0.2497961	0.2389537
6780.	0.2465953	0.2390198	0.2410817	0.2494884	0.2390856
6790.	0.2460325	0.2382108	0.2403343	0.248864	0.2386107
6800.	0.2457599	0.2376744	0.2402551	0.2482221	0.2381887
6810.	0.2450476	0.2370237	0.2400089	0.2479319	0.238215
6820.	0.2446519	0.2360653	0.2395956	0.247246	0.2378985
6830.	0.2442562	0.2355992	0.2388746	0.2467624	0.2370982
6840.	0.2439044	0.2353618	0.2384261	0.245927	0.2364739
6850.	0.2426558	0.2350892	0.2381711	0.2451707	0.2362453
6860.	0.2421106	0.2349133	0.2381887	0.2450564	0.2360166
6870.	0.2415566	0.2338757	0.2373708	0.2446695	0.2354011
6880.	0.2408355	0.2338845	0.236386	0.2442122	0.2349878
6890.	0.2407036	0.2334272	0.2361222	0.2441507	0.2346273
6900.	0.2400529	0.2328908	0.2355242	0.2439045	0.2343195
6910.	0.2397627	0.2321434	0.234724	0.2430427	0.2333962
6920.	0.2391383	0.2325567	0.2347416	0.2425151	0.2325872
6930.	0.2389801	0.2319763	0.2344514	0.2424008	0.2315495
6940.	0.2386811	0.2315982	0.2338358	0.2418644	0.2311186
6950.	0.237661	0.2308508	0.2334929	0.241240	0.2307053
6960.	0.2366146	0.2310178	0.2329829	0.2412224	0.2302921
6970.	0.2358671	0.2307453	0.2323673	0.2411521	0.2297556
6980.	0.2349262	0.2300154	0.2318485	0.240941	0.2292984
6990.	0.2346976	0.2299099	0.2312769	0.2401232	0.2289818
7000.	0.2337831	0.2294614	0.2305383	0.239068	0.2283223
7010.	0.2331411	0.2291536	0.2300898	0.2392087	0.2282344
7020.	0.2322354	0.2285821	0.2287356	0.2387866	0.2277947
7030.	0.2313033	0.2282655	0.2278123	0.2382414	0.2270736
7040.	0.231189	0.227351	0.2272055	0.2375731	0.2264317
7050.	0.2311098	0.2267794	0.2268098	0.2366586	0.2261327
7060.	0.2298436	0.2263222	0.2270033	0.2356737	0.2260448
7070.	0.2288939	0.2257594	0.2268274	0.2354714	0.2257458
7080.	0.2286213	0.2251966	0.2263438	0.235067	0.2257722
7090.	0.2279354	0.2248889	0.2259041	0.2345569	0.224972
7100.	0.2276452	0.2244316	0.2254996	0.233871	0.2245587
7110.	0.2273286	0.2237457	0.2253325	0.2338271	0.2239167
7120.	0.226757	0.222585	0.225227	0.2335105	0.2232924
7130.	0.2262382	0.2220574	0.2245587	0.2333258	0.2229758
7140.	0.2255259	0.2215474	0.2241893	0.2326663	0.2226856
7150.	0.2251126	0.2206944	0.2237145	0.2319804	0.2221756
7160.	0.2245587	0.2200965	0.2233803	0.2321211	0.2214633
7170.	0.2241014	0.2196832	0.2233539	0.2313737	0.2207335
7180.	0.2239431	0.2191468	0.2228879	0.230934	0.2199244
7190.	0.2233363	0.2185488	0.2229318	0.2302921	0.2194496

7200.	0.2230374	0.2181091	0.2219382	0.2295974	0.2192913
7210.	0.2226416	0.2181883	0.2219646	0.2294039	0.2189923
7220.	0.2219294	0.2183114	0.2211556	0.2289378	0.2181569
7230.	0.2214018	0.2179421	0.2208654	0.2283575	0.2177085
7240.	0.220540	0.2176695	0.2199596	0.2279618	0.2173743
7250.	0.2198717	0.217177	0.2199508	0.2282256	0.2165565
7260.	0.2196079	0.2163417	0.2192913	0.2278035	0.2162488
7270.	0.219221	0.2159811	0.2190715	0.2275133	0.2152727
7280.	0.2184911	0.2156382	0.2194144	0.2269945	0.2148154
7290.	0.2179547	0.2151282	0.2185351	0.2265636	0.2140767
7300.	0.2170841	0.2146885	0.2184559	0.225948	0.2134964
7310.	0.2166445	0.2140818	0.2181569	0.2257458	0.2126346
7320.	0.2156508	0.2139411	0.2174095	0.224629	0.2118784
7330.	0.2150264	0.2134311	0.2166005	0.2241278	0.2112804
7340.	0.214745	0.2130002	0.2161432	0.2236002	0.2108583
7350.	0.2144373	0.2125781	0.2154398	0.2229231	0.2101284
7360.	0.2143845	0.2124022	0.2151232	0.2218151	0.2097064
7370.	0.2140152	0.2120769	0.2153782	0.2213754	0.2097064
7380.	0.213681	0.2118922	0.2145692	0.2209533	0.2095217
7390.	0.2134436	0.2114525	0.2137602	0.2202938	0.2088798
7400.	0.213127	0.2109601	0.2137514	0.2197925	0.208528
7410.	0.2125642	0.2105644	0.2132502	0.2190803	0.2079476
7420.	0.2121949	0.2097994	0.2125027	0.2184032	0.2067781
7430.	0.2119751	0.2095883	0.2118784	0.2178316	0.2061713
7440.	0.2117904	0.2093685	0.2117728	0.2169259	0.2052656
7450.	0.2108759	0.2089816	0.2108671	0.2166796	0.2052216
7460.	0.2100581	0.2085859	0.210190	0.2159586	0.2048875
7470.	0.2093986	0.2080759	0.2093107	0.2152287	0.2041928
7480.	0.2092315	0.2076186	0.208616	0.2153606	0.2033134
7490.	0.2089765	0.2077593	0.2083785	0.2151232	0.2029441
7500.	0.2086072	0.2075659	0.2074288	0.2145868	0.2022406
7510.	0.2082026	0.2073372	0.2069628	0.2139624	0.2019944
7520.	0.2075607	0.2065722	0.2063472	0.2134524	0.2013965
7530.	0.2076311	0.2065546	0.2053623	0.2125643	0.2009304
7540.	0.207719	0.2058775	0.2046764	0.2121422	0.2009128
7550.	0.2072354	0.2057105	0.2043423	0.211852	0.2001829
7560.	0.2067869	0.2052884	0.2041664	0.2117992	0.1998224
7570.	0.2063384	0.2048399	0.2038323	0.2114651	0.1991981
7580.	0.2064264	0.2039606	0.2029969	0.2109375	0.1986968
7590.	0.2059339	0.2034066	0.2029001	0.2098998	0.1989167
7600.	0.2054678	0.202281	0.2024693	0.2095657	0.1988375
7610.	0.2051073	0.201305	0.202522	0.2087127	0.198266
7620.	0.2049139	0.2010236	0.2016691	0.2082642	0.197879
7630.	0.2045709	0.2008038	0.2015723	0.207675	0.1979406
7640.	0.203885	0.2004081	0.2015284	0.2074288	0.1977823
7650.	0.2038323	0.2004168	0.2015196	0.2070067	0.1976504
7660.	0.2038938	0.2003113	0.2009216	0.2067869	0.1970788
7670.	0.2031112	0.199432	0.2010975	0.2065143	0.1970964
7680.	0.2026363	0.198878	0.200948	0.2067957	0.1963753
7690.	0.2023461	0.1987989	0.2002445	0.2066286	0.1958829
7700.	0.2021175	0.1982009	0.1994531	0.2056701	0.1951618
7710.	0.2017746	0.197216	0.1990925	0.2050458	0.1947134
7720.	0.2011151	0.1967852	0.1991453	0.2037443	0.1940187
7730.	0.2005259	0.195642	0.1988024	0.2033486	0.1931569
7740.	0.2003236	0.1950441	0.1986353	0.2028474	0.1930602
7750.	0.2000334	0.1945868	0.1982396	0.202267	0.1924798

7760.	0.199330	0.1944022	0.1979582	0.2023989	0.1919522
7770.	0.1984242	0.1938042	0.197369	0.202012	0.1915741
7780.	0.197369	0.1934964	0.1972459	0.2014492	0.1911959
7790.	0.1972899	0.193004	0.1964105	0.2007018	0.1907387
7800.	0.1969821	0.1923357	0.196050	0.2003324	0.1904485
7810.	0.1963226	0.1916674	0.1959532	0.2006402	0.1895867
7820.	0.1959181	0.191175	0.1957158	0.2001302	0.1890415
7830.	0.1952761	0.1911398	0.1955487	0.1997345	0.1892086
7840.	0.194898	0.1908496	0.195241	0.1990134	0.1887689
7850.	0.1949244	0.190410	0.1947397	0.1985561	0.1888744
7860.	0.1950387	0.1904012	0.1941857	0.1982132	0.1884348
7870.	0.1946166	0.1898208	0.1939219	0.1975097	0.1880127
7880.	0.1942209	0.1892844	0.1941945	0.1975273	0.1876873
7890.	0.1937988	0.1887744	0.1935966	0.1967622	0.1877401
7900.	0.1935174	0.1880094	0.1938164	0.1965952	0.1875906
7910.	0.193280	0.1876225	0.1935086	0.1955487	0.1874147
7920.	0.1927876	0.1870421	0.1929195	0.1951354	0.1867024
7930.	0.1925501	0.1860748	0.1925326	0.1949947	0.1859286
7940.	0.1921808	0.1856088	0.1918818	0.1945287	0.1856736
7950.	0.191873	0.1849317	0.1915917	0.1944232	0.1851548
7960.	0.1914597	0.1851427	0.1912575	0.1943616	0.1849437
7970.	0.191064	0.1845624	0.1909849	0.1939395	0.184548
7980.	0.190765	0.1841579	0.1908002	0.1936493	0.1839588
7990.	0.1906859	0.1837973	0.1907035	0.1932712	0.1835544
8000.	0.1907914	0.1829883	0.1908178	0.1930778	0.1831586
8010.	0.1910288	0.1825926	0.1906507	0.1927612	0.1832114
8020.	0.1906155	0.1826278	0.1901583	0.1925238	0.1827453
8030.	0.1901407	0.1823552	0.1894372	0.1918906	0.1822793
8040.	0.1897626	0.1823464	0.1887601	0.1910113	0.1822881
8050.	0.1895427	0.1819331	0.1887513	0.1905804	0.1816286
8060.	0.188892	0.1813176	0.1879511	0.1907826	0.1811625
8070.	0.1886722	0.1809307	0.1877753	0.1904573	0.1808283
8080.	0.1889536	0.1802272	0.1877928	0.1902023	0.1805733
8090.	0.1887953	0.1796996	0.1868519	0.1902111	0.1798435
8100.	0.1883908	0.1799282	0.1864738	0.1900879	0.1798523
8110.	0.1875818	0.1794182	0.1861396	0.1896219	0.1792367
8120.	0.1875114	0.179128	0.1855593	0.189446	0.1784277
8130.	0.1871949	0.1784509	0.1848998	0.1888569	0.1778737
8140.	0.1870366	0.1782487	0.184548	0.1882941	0.177434
8150.	0.1866233	0.1777299	0.1838973	0.187784	0.1770383
8160.	0.186166	0.1777914	0.1836687	0.1876873	0.1771263
8170.	0.1857967	0.1775188	0.1833081	0.1872037	0.1764052
8180.	0.1854977	0.1769912	0.183273	0.1868168	0.175860
8190.	0.1849525	0.1766835	0.1831059	0.1867024	0.1756841
8200.	0.1843545	0.1767714	0.1827014	0.1861221	0.1752708
8210.	0.1838797	0.1763054	0.1818572	0.1855769	0.1745937
8220.	0.1836159	0.1760504	0.1814966	0.1853658	0.174198
8230.	0.1834136	0.1757162	0.1809163	0.1850317	0.1735385
8240.	0.1827717	0.1756195	0.1805206	0.1844601	0.1728966
8250.	0.1821737	0.1749424	0.1802919	0.1838709	0.1724657
8260.	0.1814087	0.174063	0.1799402	0.1830971	0.1718765
8270.	0.1805733	0.173553	0.1795005	0.1828772	0.1718062
8280.	0.1799314	0.1733948	0.179140	0.1825255	0.1710939
8290.	0.1799754	0.1729023	0.1789641	0.1824815	0.1709444
8300.	0.1796676	0.1721373	0.1787531	0.1820682	0.1704696
8310.	0.179351	0.1721285	0.1785684	0.1820331	0.1698892

8320.	0.1794741	0.1715921	0.1780584	0.181822	0.169854
8330.	0.179052	0.1710469	0.1779968	0.1810658	0.1696781
8340.	0.1783046	0.1705721	0.1773637	0.1803799	0.169256
8350.	0.1782518	0.1703698	0.1773109	0.1797907	0.1688867
8360.	0.1775308	0.170449	0.1769944	0.1797116	0.1686669
8370.	0.1774692	0.1701764	0.176414	0.1794829	0.1681393
8380.	0.1773549	0.1698158	0.176115	0.1791312	0.1677699
8390.	0.1768009	0.1694729	0.1756489	0.1785596	0.167638
8400.	0.1765723	0.1691036	0.175306	0.1781199	0.1672951
8410.	0.1760798	0.1685408	0.174796	0.1773021	0.1666004
8420.	0.1757984	0.1680308	0.1744706	0.1773813	0.1661519
8430.	0.1754995	0.1679253	0.1741453	0.1772933	0.1657562
8440.	0.1753763	0.1679429	0.1738551	0.1773373	0.1650967
8450.	0.1749982	0.1679868	0.1735209	0.1771526	0.1643229
8460.	0.1744706	0.1672833	0.1730724	0.1768976	0.1639623
8470.	0.1742508	0.1673801	0.1728702	0.1767657	0.1635666
8480.	0.1736792	0.1670723	0.1727031	0.176625	0.1631709
8490.	0.173178	0.1668788	0.1722283	0.1763788	0.162696
8500.	0.1727295	0.1666062	0.1718677	0.1762293	0.1617903
8510.	0.1729581	0.1663776	0.1718853	0.1759655	0.1613594
8520.	0.1729493	0.1658588	0.1718765	0.175860	0.1612891
8530.	0.1724921	0.1655686	0.1713401	0.1752972	0.1611836
8540.	0.1720348	0.1655774	0.1709796	0.1752796	0.1606911
8550.	0.1717358	0.1649795	0.1707158	0.1753148	0.1602426
8560.	0.1713929	0.1643903	0.1704696	0.1752444	0.1593105
8570.	0.1711554	0.1638715	0.1698188	0.1754203	0.1590116
8580.	0.1711466	0.1639243	0.1692121	0.1753675	0.1586071
8590.	0.1711642	0.1637044	0.1686141	0.1749367	0.1583608
8600.	0.1707773	0.163467	0.1680601	0.1743739	0.1583257
8610.	0.1707509	0.1633439	0.1674973	0.1737671	0.1579827
8620.	0.1704432	0.1632296	0.1673566	0.1731076	0.1574815
8630.	0.1701266	0.1628163	0.1666004	0.1723514	0.1569187
8640.	0.169810	0.1624206	0.166064	0.1720524	0.156180
8650.	0.1694671	0.1621304	0.1657122	0.1721403	0.155925
8660.	0.169045	0.1617611	0.165466	0.1720436	0.1558723
8670.	0.1686845	0.1614533	0.1651846	0.1716918	0.155714
8680.	0.1684031	0.1611456	0.164912	0.1716655	0.1555557
8690.	0.1682272	0.1606707	0.1639447	0.1712082	0.1550281
8700.	0.1678754	0.1608993	0.1636897	0.1710059	0.1545181
8710.	0.1672863	0.1604069	0.1630038	0.1705663	0.1539728
8720.	0.1668202	0.1606179	0.1629599	0.1703113	0.1541663
8730.	0.1661519	0.1604948	0.1624938	0.1698628	0.1537794
8740.	0.1655803	0.1603717	0.1622388	0.1691329	0.1540872
8750.	0.165167	0.1600903	0.1620365	0.1687548	0.1538673
8760.	0.1655012	0.1594572	0.1616144	0.1684558	0.1535068
8770.	0.1652374	0.1590351	0.1613594	0.168280	0.1532166
8780.	0.1646746	0.158446	0.161377	0.1678491	0.1528912
8790.	0.1644635	0.1581382	0.1609022	0.167981	0.1524604
8800.	0.1639887	0.1574875	0.159970	0.167427	0.1519679
8810.	0.1640415	0.1567489	0.1595304	0.1672775	0.1517569
8820.	0.1635314	0.1563004	0.159161	0.1666971	0.1513436
8830.	0.1633556	0.1556937	0.1586598	0.166530	0.1511941
8840.	0.1629159	0.1557024	0.1583432	0.1664773	0.1508072
8850.	0.1629862	0.1551836	0.1584488	0.1662574	0.1509391
8860.	0.1625378	0.1543395	0.1578684	0.1658441	0.1505522
8870.	0.162230	0.1539965	0.1575079	0.1656067	0.1503059

8880.	0.1617375	0.153847	0.1569539	0.1655979	0.149664
8890.	0.1614122	0.1535305	0.1565054	0.1652286	0.1491012
8900.	0.1610868	0.1533282	0.1561976	0.1645867	0.1486703
8910.	0.1604273	0.1530732	0.155925	0.1642261	0.1482834
8920.	0.1599876	0.1528622	0.1557931	0.1637425	0.1476591
8930.	0.1601723	0.1525544	0.1554238	0.1635051	0.1477558
8940.	0.159970	0.1526072	0.1546939	0.1629159	0.1471667
8950.	0.1597326	0.1522115	0.154650	0.162740	0.1469468
8960.	0.1588005	0.1521763	0.1544301	0.1621597	0.1464192
8970.	0.1583696	0.1515695	0.1542103	0.161676	0.1459004
8980.	0.1582201	0.1511738	0.1537882	0.1611308	0.1457685
8990.	0.1579475	0.150910	0.1535156	0.1611308	0.1453376
9000.	0.1573584	0.1508485	0.1530583	0.1610341	0.1450386
9010.	0.1572089	0.1510068	0.1525923	0.1607263	0.1442824
9020.	0.1570154	0.1505671	0.1524604	0.1597942	0.1444494
9030.	0.1571473	0.1500219	0.1523724	0.1596447	0.1440098
9040.	0.1570682	0.1497229	0.1524692	0.1594952	0.1435613
9050.	0.156180	0.1497317	0.1523373	0.159293	0.1432095
9060.	0.155714	0.1495734	0.1522053	0.1590819	0.1428842
9070.	0.1555557	0.1490986	0.1519327	0.1583257	0.1426556
9080.	0.1555029	0.148826	0.1513963	0.1581938	0.1419785
9090.	0.1551248	0.148272	0.1510358	0.1580531	0.1416619
9100.	0.1542542	0.1487557	0.1507984	0.1579651	0.1409056
9110.	0.1538058	0.1483424	0.150473	0.1578068	0.1408529
9120.	0.1534628	0.1485446	0.1501125	0.1572528	0.1403517
9130.	0.1531199	0.1478851	0.1499982	0.1567868	0.1400263
9140.	0.1529968	0.1475246	0.1494618	0.1568044	0.1399032
9150.	0.1528737	0.1474191	0.1492947	0.1564263	0.139657
9160.	0.1524252	0.1468387	0.1488814	0.1561273	0.1391381
9170.	0.1521614	0.1470497	0.1485209	0.1558195	0.1388567
9180.	0.1518096	0.146399	0.1484769	0.155371	0.1384786
9190.	0.1516514	0.1464342	0.1485736	0.1548786	0.1381445
9200.	0.1513963	0.146443	0.1481603	0.1542103	0.1377312
9210.	0.1512029	0.1464166	0.1479932	0.1542982	0.1374586
9220.	0.1513612	0.1457923	0.1473689	0.1537618	0.1374674
9230.	0.1511589	0.1452383	0.1466654	0.1533133	0.1373706
9240.	0.1507192	0.1453175	0.1464368	0.1528561	0.137186
9250.	0.1505346	0.1451416	0.1459531	0.1525747	0.1370629
9260.	0.1504466	0.1447811	0.1458828	0.1522053	0.1368342
9270.	0.1503323	0.1445172	0.1458476	0.1517393	0.1366671
9280.	0.1498926	0.1446052	0.1456454	0.1515722	0.136289
9290.	0.1498487	0.1441479	0.1454607	0.1510974	0.1362363
9300.	0.1494969	0.1437874	0.1453464	0.1506929	0.1361132
9310.	0.1495409	0.143251	0.1449419	0.1501828	0.1360692
9320.	0.1492771	0.1427762	0.1445286	0.1499278	0.1357438
9330.	0.1490485	0.1423453	0.144511	0.1497344	0.1354361
9340.	0.1484505	0.1422134	0.1440713	0.1492947	0.1352338
9350.	0.1484329	0.1417122	0.1437196	0.1492243	0.1347062
9360.	0.1481779	0.1412549	0.1436053	0.1488462	0.1341786
9370.	0.1481075	0.1409823	0.143192	0.1487231	0.1340379
9380.	0.1480812	0.140912	0.1427611	0.1486264	0.1336422
9390.	0.1475975	0.1408064	0.1424269	0.1485648	0.1332992
9400.	0.147281	0.140525	0.1421455	0.1485209	0.1331058
9410.	0.1468149	0.1410263	0.1417234	0.1482658	0.1326309
9420.	0.146384	0.1405954	0.1413805	0.1479053	0.1323847
9430.	0.1463049	0.1403844	0.1410639	0.1472634	0.1321912

9440.	0.1460147	0.1401206	0.1407034	0.1469556	0.1320418
9450.	0.1455047	0.139804	0.1401934	0.1470084	0.1317252
9460.	0.1448803	0.1393379	0.1396658	0.146472	0.1314614
9470.	0.1443263	0.1391093	0.1396306	0.1460938	0.1315581
9480.	0.1437987	0.1386081	0.1394283	0.1457685	0.1312943
9490.	0.1434206	0.1385817	0.139270	0.1454431	0.1311536
9500.	0.1433415	0.1380277	0.1390238	0.1451793	0.1306172
9510.	0.1432359	0.1377112	0.1386457	0.1449155	0.1298258
9520.	0.1429369	0.1373067	0.1382676	0.1447836	0.1299137
9530.	0.1424445	0.137078	0.1385578	0.1446077	0.1299313
9540.	0.1424797	0.136401	0.1381972	0.1442912	0.1297027
9550.	0.1422159	0.1362339	0.1374498	0.1437899	0.1294125
9560.	0.1417586	0.1360228	0.137353	0.1434206	0.1292718
9570.	0.1418905	0.1356887	0.136931	0.143359	0.1291751
9580.	0.1417146	0.1355832	0.1367727	0.1432008	0.1292718
9590.	0.1412486	0.1350556	0.1365792	0.1429633	0.1288761
9600.	0.1413629	0.1352138	0.1367551	0.1425764	0.1282165
9610.	0.141187	0.134695	0.1365265	0.1426819	0.1280055
9620.	0.1407386	0.1344488	0.1361659	0.1422862	0.1275307
9630.	0.1405099	0.1343697	0.1354976	0.1420224	0.1274955
9640.	0.1404572	0.1341498	0.1357702	0.1417059	0.1270558
9650.	0.1400966	0.1341762	0.1355592	0.1416355	0.1268008
9660.	0.1401142	0.1340707	0.1351722	0.1416091	0.1266073
9670.	0.1397537	0.1338069	0.1350755	0.1415124	0.1266865
9680.	0.139569	0.1336662	0.1349964	0.1408968	0.1262292
9690.	0.1394547	0.1332353	0.1345919	0.1405451	0.125728
9700.	0.1389886	0.1328045	0.1343984	0.140677	0.125473
9710.	0.1386017	0.1326286	0.134372	0.1401142	0.1252355
9720.	0.1382324	0.132400	0.1338532	0.1400351	0.1246815
9730.	0.1381269	0.1320658	0.1336158	0.1398856	0.1241012
9740.	0.1382236	0.1318372	0.1330618	0.1394283	0.1238462
9750.	0.1381269	0.1315118	0.1333432	0.1392437	0.1236703
9760.	0.1378719	0.1313008	0.1330706	0.1389007	0.1232394
9770.	0.1375113	0.1311073	0.1326221	0.1382676	0.1230284
9780.	0.137142	0.1311689	0.1320769	0.1378455	0.1224568
9790.	0.1369046	0.1307556	0.1318659	0.1375465	0.1221138
9800.	0.1364913	0.1300257	0.1317955	0.1372387	0.1218324
9810.	0.1366056	0.1295421	0.1314438	0.1370277	0.1214455
9820.	0.1364913	0.1290497	0.1313559	0.1368518	0.1210938
9830.	0.1360164	0.1285484	0.1312503	0.1363682	0.1209179
9840.	0.1357878	0.1281352	0.1308019	0.1363066	0.120531
9850.	0.1356999	0.1276163	0.1305293	0.1358757	0.1206277
9860.	0.1355943	0.1272206	0.1301687	0.1358669	0.1204255
9870.	0.1355328	0.1267458	0.1301247	0.1357262	0.1202232
9880.	0.1352778	0.1266842	0.1298785	0.1351635	0.1202672
9890.	0.1353042	0.1266227	0.1295356	0.1348733	0.1202056
9900.	0.1352338	0.1266051	0.1290431	0.1344072	0.1198979
9910.	0.1346534	0.1265699	0.1286386	0.1340555	0.1193439
9920.	0.1346358	0.1266842	0.1286386	0.1335103	0.1190185
9930.	0.1341962	0.1263589	0.1280671	0.1333784	0.1187723
9940.	0.1336334	0.1256202	0.1278736	0.1329563	0.1186316
9950.	0.1335454	0.1252949	0.1276362	0.1326661	0.1184733
9960.	0.1334839	0.1248376	0.1272756	0.1319274	0.1182183
9970.	0.133264	0.1248376	0.1268975	0.131646	0.1177346
9980.	0.1330618	0.1245563	0.1263347	0.1312064	0.1175676
9990.	0.132798	0.1247497	0.126238	0.1311536	0.1172158
